# Theranostics with photodynamic therapy for personalized medicine: to see and to treat

**DOI:** 10.7150/thno.87363

**Published:** 2023-10-09

**Authors:** Youchao Wang, Johannes Nikodemus Staudinger, Thomas L. Mindt, Gilles Gasser

**Affiliations:** 1 Chimie ParisTech, PSL University, CNRS, Institute of Chemistry for Life and Health Sciences, Laboratory for Inorganic Chemical Biology, 75005 Paris, France.; 2 Institute of Inorganic Chemistry, Faculty of Chemistry, University of Vienna, Währingerstraße 42, 1090 Vienna, Austria.; 3 Vienna Doctoral School in Chemistry, University of Vienna, Währingerstraße 42, 1090 Vienna, Austria.; 4 Division of Nuclear Medicine, Department of Biomedical Imaging and Image Guided Therapy, Medical University of Vienna, Währinger Gürtel 18-20, 1090 Vienna, Austria.; 5 Joint Applied Medicinal Radiochemistry Facility, University of Vienna, Währingerstraße 42, and Medical University of Vienna, Währinger Gürtel 18-20, 1090 Vienna, Austria.

**Keywords:** Magnetic Resonance Imaging (MRI), Nuclear Imaging (PET, SPECT), Optical Imaging, Photodynamic Therapy (PDT), Theranostics

## Abstract

Photodynamic Therapy (PDT) is an approved treatment modality, which is presently receiving great attention due to its limited invasiveness, high selectivity and limited susceptibility to drug resistance. Another related research area currently expanding rapidly is the development of novel theranostic agents based on the combination of PDT with different imaging technologies, which allows for both therapy and diagnosis. This combination can help to address issues of suboptimal biodistribution and selectivity through regional imaging, while therapeutic agents enable an effective and personalized therapy. In this review, we describe compounds, whose structures combine PDT photosensitizers with different imaging probes - including examples for near-infrared optical imaging, magnetic resonance imaging (MRI) and nuclear imaging (PET or SPECT), generating novel theranostic drug candidates. We have intentionally focused our attention on novel compounds, which have already been investigated preclinically *in vivo* in order to demonstrate the potential of such theranostic agents for clinical applications.

## 1. Introduction

Theranostics is an emerging clinical approach, which combines diagnosis and specific targeted therapy in dual-functional molecules to achieve a personalized treatment 1-3. Compared with commonly used treatment strategies, where separate methods are used for diagnostics and therapy, theranostic compounds integrate various features into a single structure to overcome issues in biodistribution and pharmacokinetic performance that can occur during conventional treatments. Furthermore, it allows monitoring drug delivery in real time, gives important information of treatment effects, and accomplishes the goal of personalized precision medicine. The therapeutic part of theranostics mainly includes agents for chemotherapy, photodynamic therapy (PDT), photothermal therapy (PTT), or radiation therapy (RT). In comparison, various imaging modalities such as magnetic resonance imaging (MRI), positron emission tomography (PET), single photon emission computed tomography (SPECT) and near infrared (NIR) luminescence/fluorescence are used in the diagnostic part.

PDT has gained recognition as an efficient tumor treatment modality due to its high selectivity, minimal invasiveness and limited susceptibility to drug resistance, as well as a considerable potential to be used in conjunction with other treatment or imaging modalities 4, 5. This type of therapy is based on the use of a photosensitizer (PS), which produces reactive oxygen species (ROS) upon light irradiation. After accumulation in the target tissue, the PS is activated by light irradiation of a certain wavelength, and subsequently produces ROS *via* energy and/or electron transfer, which in turn induces cell death. Nowadays, PDT has already successfully reached the clinic for the treatment of various tumors, as well as in antibacterial and antiviral applications^6^, ^7^. Currently, the most common therapeutic modality is topical PDT for the treatment of skin cancer, which by now has likely been successfully applied millions of times worldwide. The therapeutic outcomes are hereby often equal to surgery, but come with the advantage of being non-invasive, which leads to shorter healing times and superior cosmetic results. Besides this skin cancer, PDT is also investigated and used for the treatment of several other cancer types (e.g. lung, breast, esophagus and bladder cancer), as well as an adjuvant therapy (e.g. for age related macular degeneration), but has not reached the level of a first line treatment in most cases 5. Furthermore, it was recently reported that PDT and PTT can induce immunogenic cell death, which results in the release of various damage-related molecular signals. This can synergize well with immunotherapy, which can otherwise be limited in its scope, since tumors often create their own immunosuppressive microenvironment 5, 8, 9. In recent years, the photosensitizer-precursor 5-aminolevulinic acid (5-ALA; Gleolan^®^), which is approved for the PDT treatment of actinic keratosis, also got approval as an imaging agent during glioma surgery, which underlines the potential of photosensitizers, and their derivatives, as theranostic agents 10, 11.

**Figure 1:**
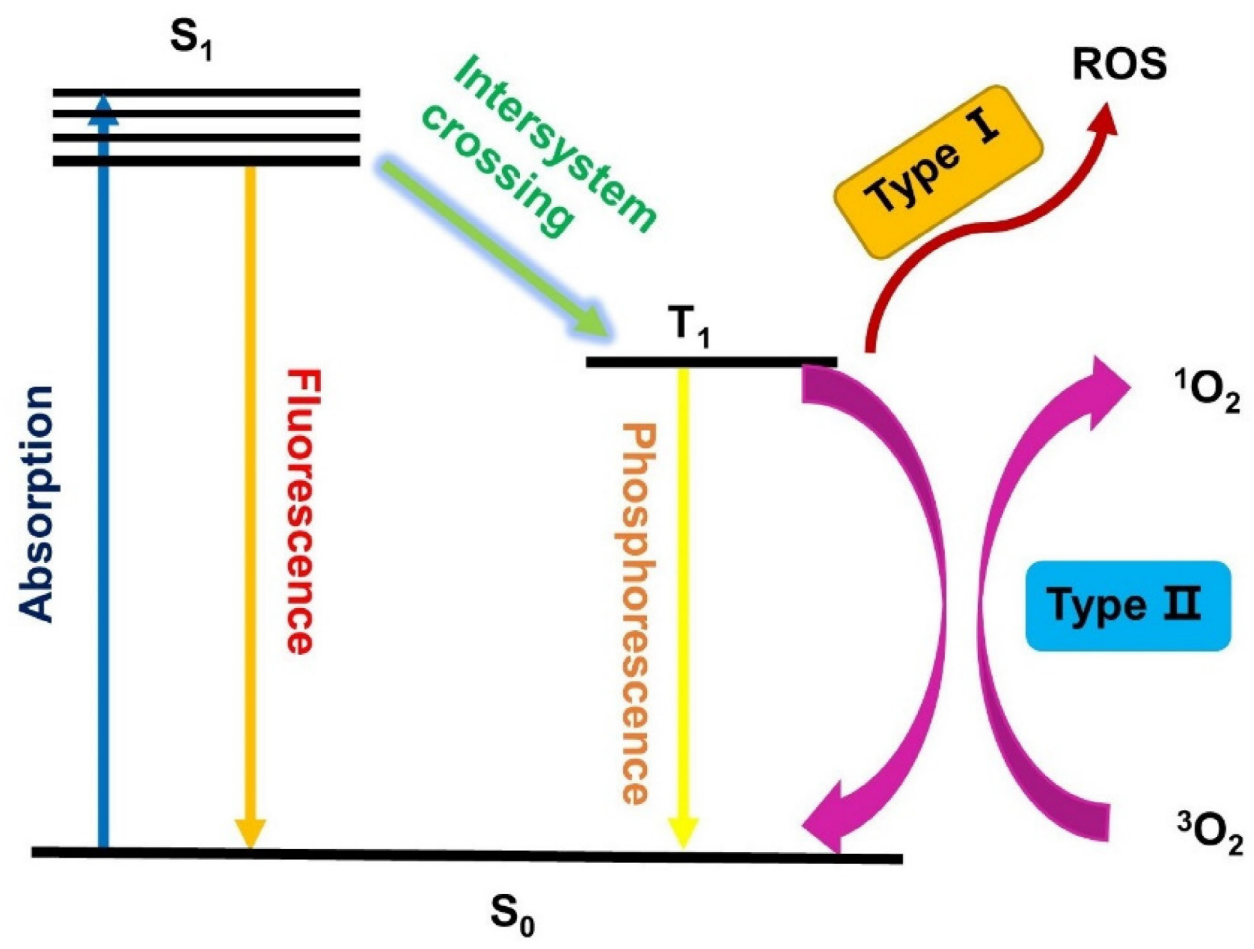
Schematic illustration of a typical photodynamic reaction.

As shown in Figure 1, PDT is a multistage process, in which a PS is first activated from the ground state (S_0_) to a short-lived excited singlet state (S_1_) through absorption of a photon. However, the excited singlet PS is not very stable and may return to the S_0_ state by emitting fluorescence, which facilitates the use of sensitive assays to measure the pharmacokinetics and distribution of PS in living animals or patients 12, 13. Alternatively, the excited PSs (S_1_) can also undergo an intersystem crossing (ISC) to a long-lived excited triplet state (T_1_), which is relatively stable and can influence the biological environment *via* two different procedures. In Type I reactions, the excited PS (T_1_) directly reacts with substrates in the cellular microenvironment *via* the transfer of an electron or proton, and the generated radicals can further interact with oxygen to produce ROS, such as superoxide anion radicals (O_2_^•-^) 14. Alternatively, in the Type II pathway, the excited triplet PS transfers its energy to molecular oxygen directly, which results in the formation of highly reactive singlet oxygen (^1^O_2_). It has been reported that both pathways can occur simultaneously, depending on the used PSs, the oxygen concentration and possible interactions with the substrate.

As a result, oxygen (^3^O_2_), light and PSs are the three indispensable elements of PDT. ^3^O_2_ is critical since it is necessary for the generation of ^1^O_2_ and other reactive oxygen species as a way to directly damage tumors. However, since the proliferation and growth of tumor cells consume a massive quantity of oxygen, many tumor sites have a hypoxic microenvironment, which reduces the effect of PSs. As a result, PDT is often less effective *in vivo* than what would been expected according to results of *in vitro* experiments 15, 16.

The ability of light to pass through biological tissue depends on its wavelength, as well as the properties of the respective tissue, where the light is partially scattered at heterogeneous components, such as cell membranes and water. Furthermore, the presence of endogenous dyes with higher light absorption capacity, such as melanin and hemoglobin, also seriously affects the penetration depth. However, the concentration of such endogenous dyes greatly varies between different tissues, and therefore the penetration depth of light is also influenced by the type of tissue. Generally speaking, longer wavelengths lead to a deeper tissue penetration and most tissues have limited scattering and absorption of light in certain parts of the NIR-range (e.g. 700-900 nm and 1000-1800 nm). Those are therefore referred to as "phototherapeutic windows" and used in PDT whenever possible 17-20.

At the heart of PDT are the PSs, which transfer energy to surrounding oxygen or other components, thereby producing ROS. Therefore, the study of PSs is crucial for the development of PDT. Ideally, PSs should have the following characteristics: (1) high selectivity, which means they preferentially accumulate in tumor tissues and are rapidly cleared from normal tissues; (2) no dark toxicity; (3) high photostability (i,e., they are not photobleaching), and no reaction with ^1^O_2_ and other reactive oxygen species, so that the PS does not fade during PDT and can be repeatedly excited in a catalytic manner; (4) strong absorption in the phototherapeutic window, although this depends on the type of applications required 13, 21.

Hereby, it must be noted that photosensitizers often also induce photothermal effects upon irradiation. Mild hypothermal effects are known to be synergistic with PDT, while a significant temperature increase can lead to apoptotic or necrotic cell death and is used for photothermal therapy (PTT). To induce a PTT effect, which is inherently less selective than PDT and has a greater risk of damaging adjacent tissues, higher light fluences are required (e.g. 0.5-10 W/cm² vs. ≤ 0.2 W/cm²). However, PTT also induces a different cell death mechanism and causes other cellular changes than PDT. As a result, some therapeutic approaches use combinations of those two treatment modalities, while in others the light fluency is strictly controlled, to prevent tissue heating. To differentiate between the effects of PDT and PTT, several methods have been developed so far. Among the possibilities, the use of specific ROS scavengers to suppress the PDT effect and external influences like cooling, as well as the use of multiple short irradiation cycles, that do not heat the tissue significantly, to mimic a PDT-only treatment, can be employed. However, designing adequate controls for either of those pathways, as well as quantifying their interaction, still remains a challenge 22, 23.

**Figure 2:**
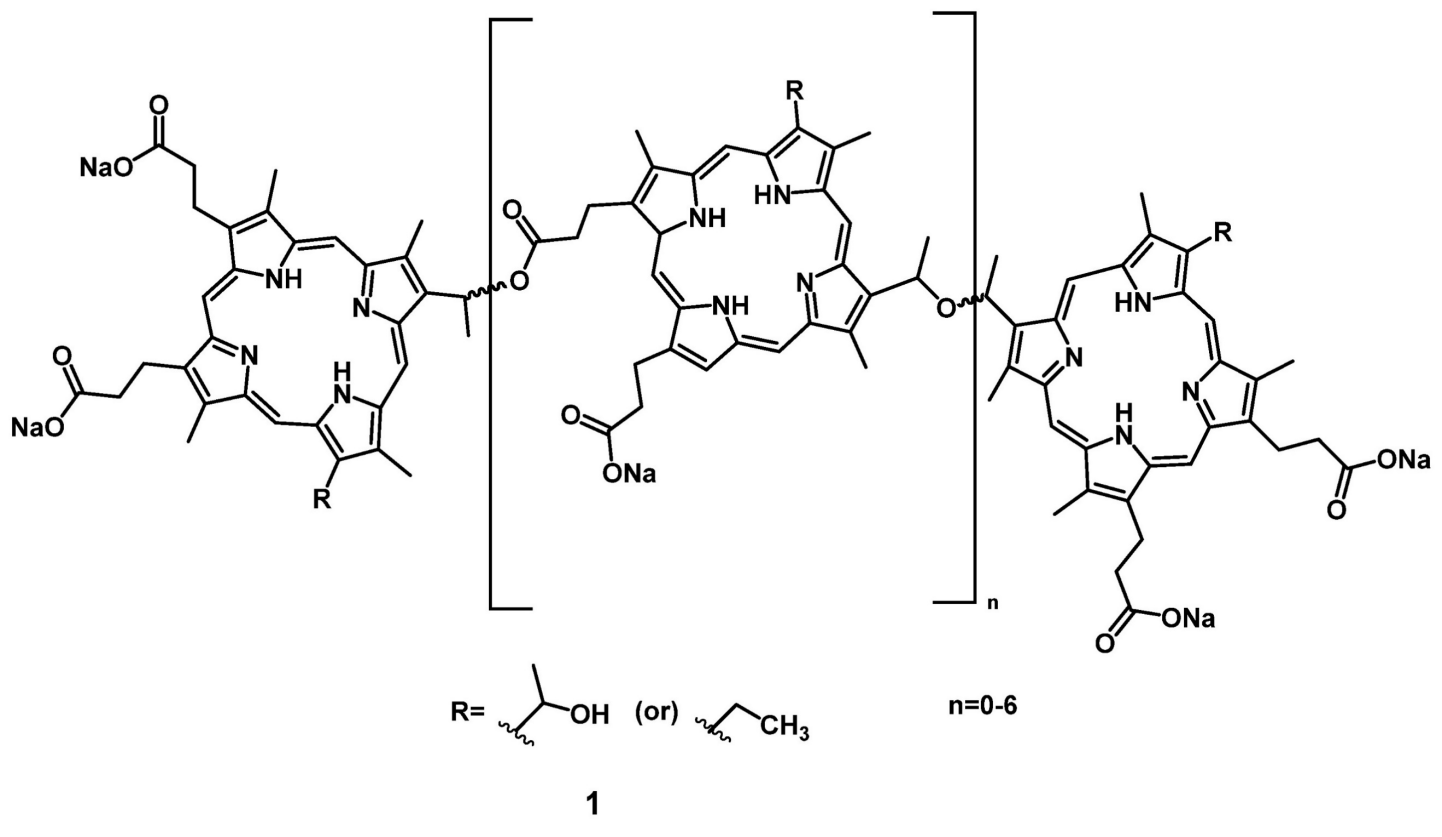
First generation photosensitizer, Photofrin^®^.

The most common PSs for PDT are porphyrins, which account for about half of all approved PSs. The first-generation of PSs were derivatives of hematoporphyrin, and often mixtures of various compounds, with the prime example being Photofrin (**1**, Figure 2), which was first approved for cancer treatment in 1993 24. However, a lack of tumor selectivity, poor tissue-penetration of the excitation light, long-term photosensitizing side effects, aggregation induced quenching effects, as well as a low bioavailability and poor biodistribution are major limitations of these first-generation photosensitizers, and restrict their clinical application 25, 26.

**Figure 3:**
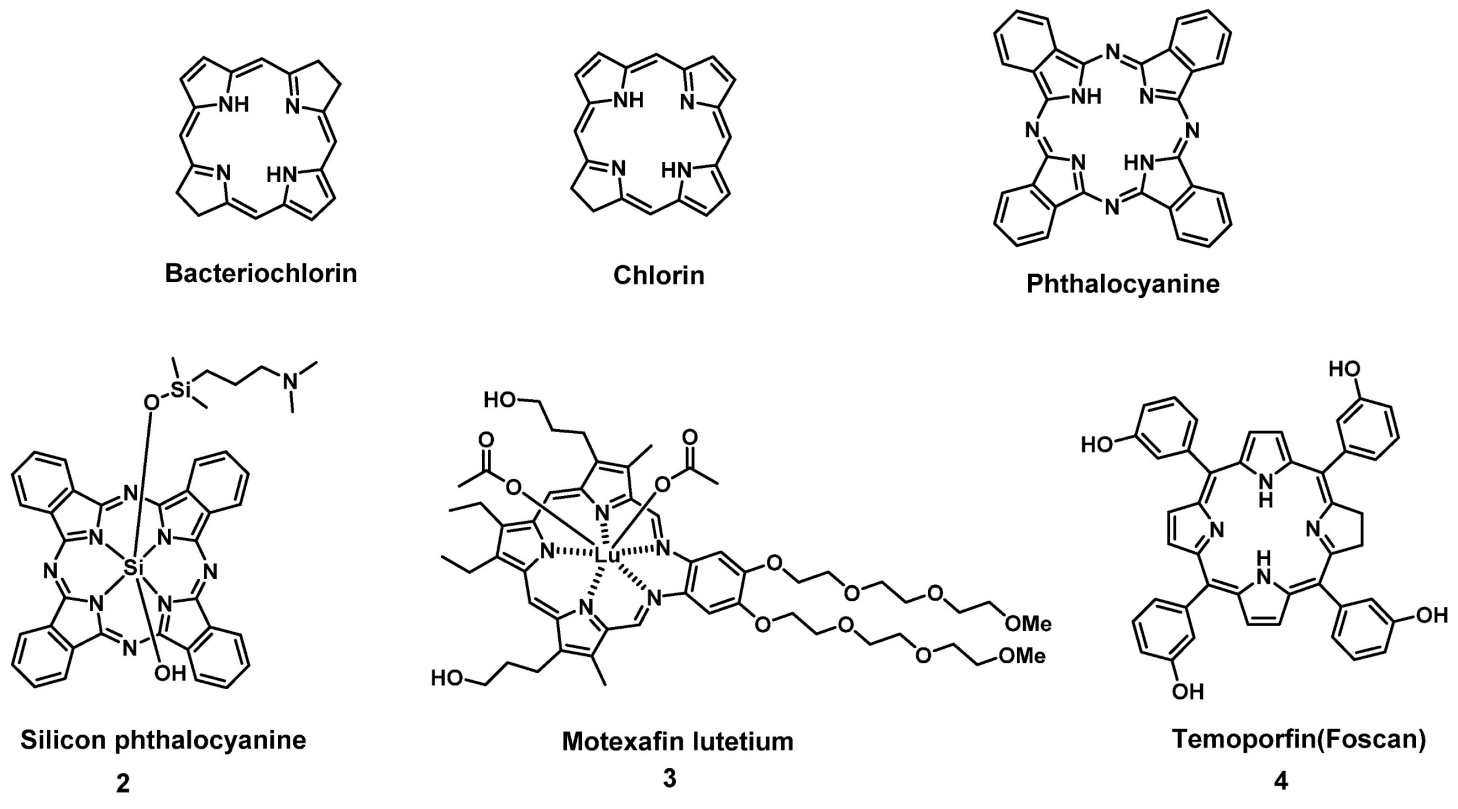
Examples of porphyrin-derived PDT second generation of PSs in clinical trials or clinical use.

**Figure 4:**
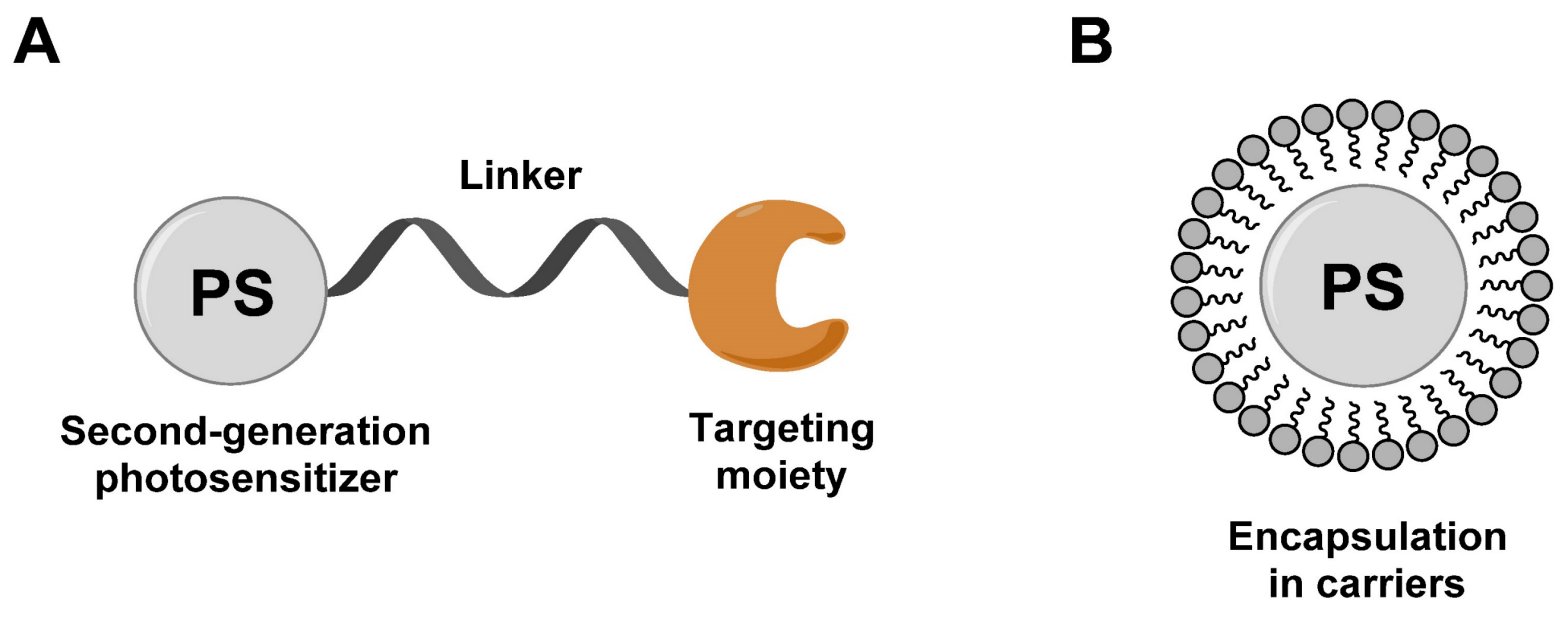
General design of third-generation photosensitizers: (A) conjugation of second-generation photosensitizer with targeting moiety; (B) encapsulation of a second-generation photosensitizer into carriers (liposomes, micelles, and nanoparticles). Redrawn from reference 29.

To circumvent the limited efficacy and selectivity, a second generation of photosensitizers was developed. These compounds were based on phthalocyanines, chlorins and bacteriochlorins, modified with additional side chains (Figure 3). The structural modifications greatly extended the absorption wavelengths towards the NIR range, where the light penetration is enhanced, increased their solubility, improved the ^1^O_2_ quantum efficiency and tumor uptake, which are all beneficial for clinical applications 26. At present, multiple second-generation photosensitizers are undergoing clinical trials or have already been approved. For example, silicon phthalocyanine (**2**) has entered a phase I clinical trial in the United States 27, Motexafin lutetium (**3**) is currently in Phase II clinical trials for prostate cancer 28, while Temoporfin (**4**) is already an approved drug. Unfortunately, these photosensitizers usually have a limited specificity for tumors, due to their passive cellular uptake (e.g. because of the higher metabolism and proliferation of tumor cells), as well as a reduced light absorbance in tissues. As a result, a third generation of photosensitizers is currently being investigated, with the focus on better tumor targeting procedures and the improvement of the optical properties in tissues (e.g., deeper tissue penetration and higher extinction coefficients) (Figure 4). To achieve this, the properties of the PDT agents can be further modified for excitation at longer wavelengths (NIR range). Furthermore, by designing compounds which are capable of aggregation-induced emission (AIE), an enhanced ROS-production and stronger luminescence upon accumulation in the targeted tissue can be induced. To improve their specificity, the compounds can also be conjugated to antibodies, targeting-proteins or targeting-ligands, as well as joined with cleavable linkers which could be activated by either chemical triggers in the target tissue (e.g. through enzymes or hypoxic conditions) or light, thus allowing their specific cellular uptake. Furthermore, by incorporating the PSs into nanocarriers (e.g. liposomes and micelles) or nanoparticles (e.g. carbon-based nanostructures), their therapeutic efficacy and targeting properties can be improved as well (e.g. through the enhanced permeability and retention (EPR) effect) 26, 29, 30. However, it must also be noted that the conjugation of PSs with additional functional moieties, to achieve an increased tumor uptake, better solubility or confer further properties, can negatively impact their therapeutic efficacy.

**Figure 5:**
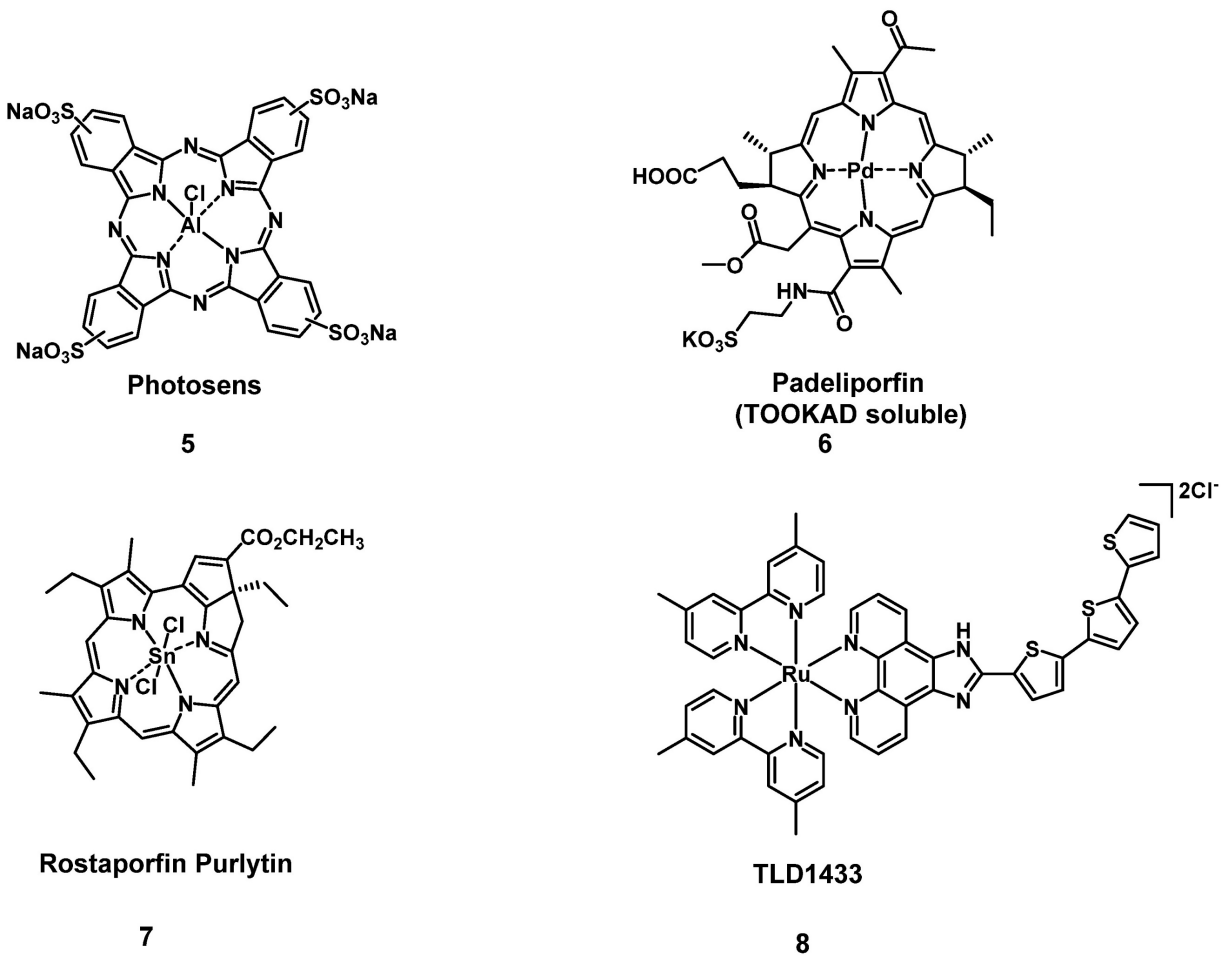
Selected therapeutically important metal-based PDT photosensitizers, which have been approved for clinical use or are currently in clinical trials.

One sub-category of PSs are transition metal-based compounds, which have been studied due to their favorable properties for PDT such as low photobleaching rates and strong PDT effects with high irradiances. By now, a variety of metal-based PSs for PDT, with different metal-centers have been investigated^31^. However, Photosens (**5**) and TOOKAD Soluble (**6**) remain the only metal-based PSs currently approved for clinical use, while compounds like Purlytin (**7**), LuTex (Motexafin lutetium **3**) and TLD1433 (**8**) have entered clinical trials (Figure 5) 32.

Since ROS, which are generated during the PDT process, have only a short lifespan and a limited mobility, the primary position of ROS generation significantly affects the therapeutic effect. As a result, methodologies to transport the PSs to vulnerable organelles in the target tissues (e.g., cancer), where they can induce their effect, have the potential to enhance the treatment outcome. There exist multiple potential cellular targets, which would be suitable for this purpose, and accessible *via* the conjugation of PSs with organelle-specific targeting molecules like certain peptides or through chemical modifications. One of the most promising targets for PDT are the mitochondria, which are essential for regulating various pathways and provide adenosine triphosphate (ATP) for the cell. Mitochondria are sensitive to ROS, which is highly compatible with PSs, that induce apoptosis when damaged and due to their strong negative membrane potential, lipophilic cations tend to accumulate there. The endoplasmic reticulum (ER) is responsible for the synthesis of proteins and cellular lipids, as well as the storage and homeostasis of Ca^2+^, and induces ER stress when damaged. The ER is rich in ATP-sensitive K^+^ channels, as well as in sulfonamide receptors, which make the targeting with glibenclamides and sulfonamides possible. The nucleus forms the control center of eukaryotic cells, which regulates metabolism, proliferation and the cell cycle, making it a prime target for various drugs. However, since only a few small water-soluble molecules can freely enter the nucleus, the modification with peptide chains or aptamers, that are actively transported there, is usually necessary for targeting this organelle. Another potential target are lysosomes, of which cancer cells possess an increased number in comparison to normal cells. These organelles are responsible for the degradation and recycling of molecules, and upon their destruction release hydrolases and protons, which can lead to cellular dysfunction such as apoptosis and necrosis. By connecting the PSs with one of several receptor binding molecules like folates and transferrin to access the endocytic pathway, or through modification with lipophilic amines, it is possible to target this cellular constituent 33, 34.

This review summarizes the development of theranostic compounds, which combine PDT and various imaging technologies (i.e., NIR luminescence/fluorescence, MRI, PET, SPECT) by joining two molecules with distinct functionalities into a single agent and thus, enabling a personalized medical treatment. In this review, we focus only on novel compounds which have not yet entered the clinic but are being investigated preclinically *in vivo* in order to demonstrate the potential of theranostic agents for medical applications. Additionally, this review discusses each type of theranostic drugs in chronological order, or for the sake of clarity, in a certain continuity of work for specific compound families. It should be noted that besides small theranostic molecules or proteins, a wide range of approaches implement nanoparticles and materials based on aggregation-induced emission (AIE) to combine PDT with various imaging modalities, which have recently shown great promise for clinical applications. There have been numerous examples that result in little recurrence and successful tumor ablation *in vivo*, which are highly valuable for clinical translation. For example, PSs can be incorporated into lipid-bubbles for photoacoustic imaging, be encapsulated in micelles, or integrated into macromolecular cages. However, inclusion of these strategies is beyond the scope of this review and instead, it is referred to other articles and reviews on the respective topics 22, 35-43.

## 2. PDT and Optical Imaging

The most prevalent imaging methodology used in combination with PDT is optical imaging, since many approved PSs have inherent luminescent properties and are already used as early diagnostics, intraoperative markers or imaging probes. However, this imaging modality has significant limitations because both activation and emission usually happen *via* UV/Vis light, which has a very limited penetration depth (defined as the depth where optical energy drops to 1/e, ~37%) in biological tissues (< 3 mm) due to light scattering and auto-fluorescence 26, 44-46. As a result we will hereby focus on recent developments in imaging methodologies, which allow to circumvent the inherent limitations of optical imaging.

One way to enhance the applicability of luminescence imaging is the use of near-infrared (NIR) light, since endogenous, biological fluorophores like hemoglobin show no, or only limited absorbance in a large part of this range (~700-2500 nm). As a result, light within the range of this wavelength has a deeper tissue penetration depth (NIR ~1 cm) and better contrast, which helps with activating compounds for PDT or luminescence emission, as well as in tissue imaging 20, 26, 46, 47. For this purpose, several optical ranges in the NIR spectrum are available, but their exact ranges are still discussed controversially in literature and depend on the referenced publication 19, 20, 48-50: 1) the NIR-I window up to 900 nm, which is more easily achieved, but still has some background signals, and 2) the NIR-II range from 1000-1800 nm, where the light attenuation in tissue is significantly reduced and biological auto-fluorescence is negligible, resulting in a deeper penetration and better contrast. Furthermore, there is 3) the NIR-III (2100-2300 nm) window, which also allows deep tissue penetration. However, compounds that emit at longer wavelengths (e.g. NIR-II vs. NIR-I) have lower quantum yields and a significantly reduced ROS generation 19, 20, 47, 51. To overcome those limitations, a variety of different methodologies are currently investigated. Among them are compounds that can be excited *via* two-photon absorption or up-conversion nanoparticles, which combine the higher penetration depth of longer wavelengths, with increased ROS production and quantum yields in the Vis/NIR-I range 52, 53. Similarly, donor-π-acceptor structures, the encapsulation into vehicles, as well as the integration of intracellular-targeting properties (e.g. mitochondria), can increase the PDT efficiency 52, 54.

To combine NIR luminescence imaging with PDT, it is necessary that the PS shows both strong luminescence signals, as well as an efficient generation of singlet oxygen (^1^O_2_) or ROS. However, for the PSs themselves, it is common that with an increasing production of reactive species (ROS, ^1^O_2_), the luminescence yields are reduced. Due to this, various methods to increase ^1^O_2_ production, or generate suitable imaging signals have been developed. Examples include the tethering of additional fluorophores (e.g., squaraines, rhodamines or BODIPYs) to the photosensitizer, the use of two-photon excitation techniques, red-shifting the absorption and emission towards the NIR-range *via* the extension of π-systems in its structure, as well as the use of luminescent transition metal complexes 44, 55-57. Until now, a variety of activatable PSs for NIR-I imaging have been developed, but those suitable for the NIR-II range are limited in number. Due to the wide range of structurally diverse compounds currently investigated for optical imaging in combination with PDT, this review will only focus on luminescent small molecules, with luminescence above 700 nm, which have already been investigated *in vivo*. Therefore, nanoparticles and compounds that induce their effect *via* aggregation induced emission (AIE) will be excluded. Such compounds have been discussed recently in various articles and reviews, to which we refer here 22, 35-43.

### 2.1. Nonmetallic photosensitizers

#### 2.1.1. Pyropheophorbide- and cyanine-based photosensitizers

One of the first *in vivo* applications of NIR-imaging for PDT was implemented by Stefflova et al., who designed a caspase-cleavable probe **9** with a peptide-linker connecting a fluorescence quencher with a pyropheophorbide PS and a folate receptor targeted therapy agent with a built-in apoptosis sensor (TaBIAS), since the folate receptor is overexpressed in several cancer types. *In vitro*, it was shown that the compound preferably accumulates in KB cells overexpressing the folate receptor, and apoptosis could be triggered through light irradiation, which in turn induced caspase cleavage and thus restored the fluorescence signal of pyropheophorbide. During *in vivo* studies on tumor-bearing nude mice, a significantly stronger fluorescence (695-770 nm) in folate receptor positive KB tumors could be observed after irradiation (615-665 nm), than in the folate-receptor lacking HT 1080 control tumor, indicating a preferential tumor accumulation on folate receptor-overexpressing cells. This allowed real-time *in vivo* NIR-imaging of the PS during PDT-treatment (670 nm, 90 J/cm²), with the caspase-3 cleavable sequence serving as an apoptosis-sensitive switch for detecting the fluorescence signal in apoptotic tissue 58.

**Figure 6:**
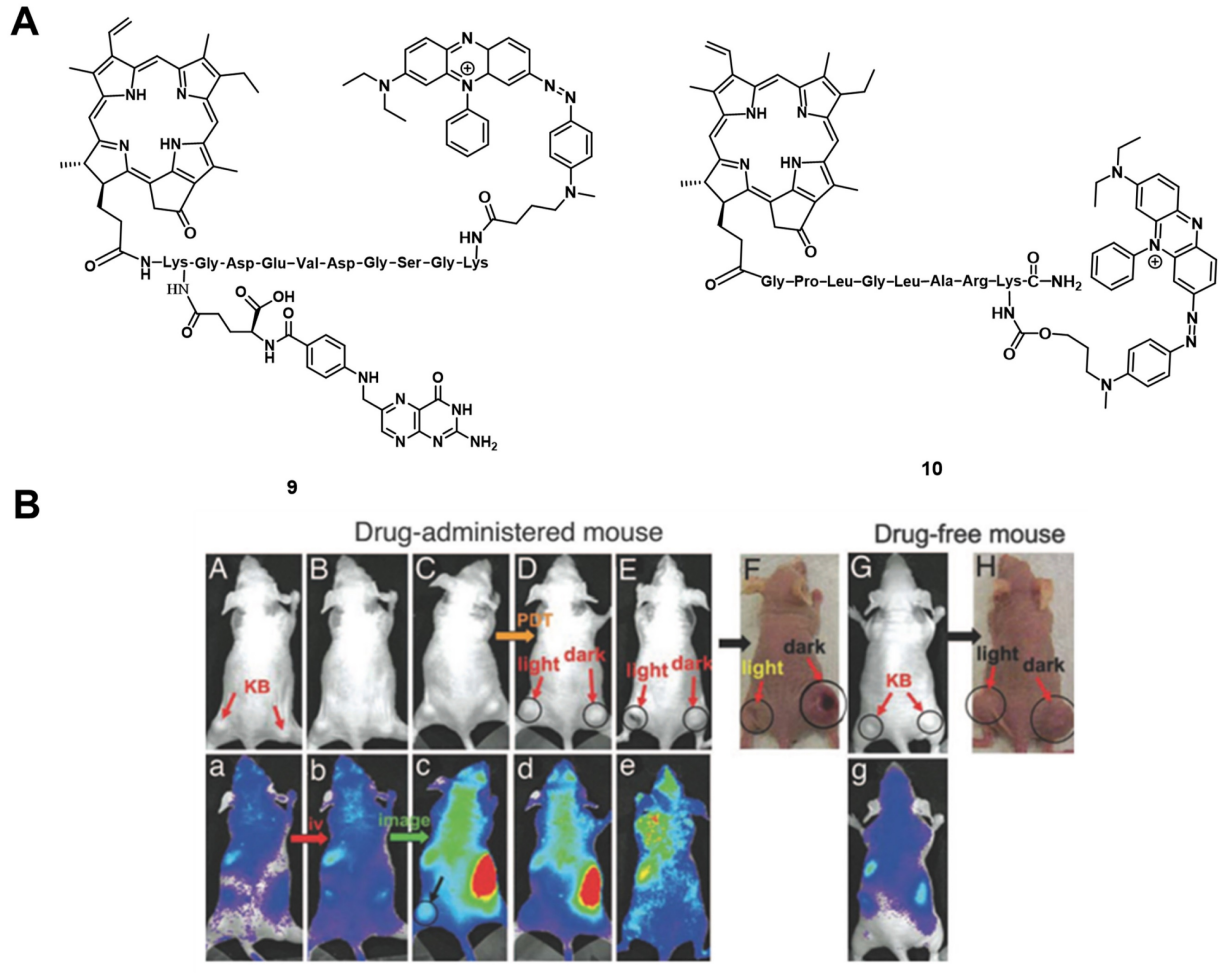
(A) Theranostic agents containing a cleavable probe combining targeting structure, fluorescence-quencher and NIR-fluorescent photosensitizer for *in vivo* imaging 58, 59; (B) NIR-images of tumor-bearing mice, showing that the compound initially has no fluorescence signal (a, b), accumulates in the tumor (c) and upon light irradiation the tumor tissue becomes edematous (d), while also inducing tumor shrinkage over time (e) 59. Reproduced with permission from ref. 59. Copyright (2007) The National Academy of Sciences, U.S.A.

Zheng et al. developed a photodynamic molecular beacon **10**, which combined a PS with a ^1^O_2_ and fluorescence quencher (Figure 6A). Initial *in vitro* experiments on the matrix metalloproteinase 7 (MMP7) positive KB cells, with BT20 as control cells, were conducted to demonstrate that this enzyme, which is often overexpressed in tumors, can cleave the linker and thus allow the induction of PDT. In the following *in vivo* study, KB-xenograft-bearing nude mice were treated with this molecular beacon. Directly after injection in the tail vein of 80 nmol of compound **10**, no region with increased luminescence signals could be observed, which proved its optically silent native state. Over the following hours (h), an increase in luminescence activity - likely due to MMP7 - could be detected and, upon light irradiation (670 nm, 135 J/cm² over 30 min), the tumor tissue turned edematous, and started to shrink (Figure 6 B). This study demonstrated the potential of such molecular beacons for PDT 59.

In 2013, James et al. reported the synthesis of a series of NIR-fluorescent cyanine-based organic dyes with indole or benzindole moieties for image-guided PDT (Figure 8). The properties of the compounds were investigated *in vitro*, and their tumor targeting and biodistribution was studied in BALB/c mice bearing colon-26 tumors. Hereby, the indole-based compounds showed a good tumor affinity but could not be coupled to the pyropheophorbide-based PS “Photochlor” (HPPH, **11**), without any additional functionalization. The 4-aminothiophenol modified structure showed a fast clearance from most organs, and was identified as the best substitute for *in vivo* tumor uptake, but also caused fluorescence quenching 60. In a follow-up study, the functionalized dyes were conjugated with the PS **11** in mono- and dimeric forms and investigated for their PDT response and imaging capabilities *in vivo* in the same mouse model. All compounds could be used for tumor imaging, even at low doses (0.03 µg/kg). Conjugate **12** (Figure 8), one of the benzindole-containing structures with two PSs **11** proved to be superior to all other conjugates since it showed an enhanced tumor uptake and contrast, while also increasing the PDT anti-tumor efficacy (665 nm, 128 J/cm² with 14mW/cm²) in BALB/c mice bearing colon-26 tumors. In contrast, the cypate-based conjugates (e.g., compound **13**) showed no PDT response *in vivo*, which might be due to photobleaching of the unstable polymethine linker upon light irradiation 61. The rate of photobleaching of HPPH-conjugates *in vitro* and *in vivo* was further studied. For this purpose, BALB/c mice bearing CT-26 tumors were treated with one of the different conjugates, and their NIR fluorescence (λ_ex_ = 710-740 nm, λ_em_ = 800 nm) monitored during the PDT treatment. However, none of the complexes was capable to completely remove the tumor, with the cypate-based compounds not yielding any long-term cure, while lower light fluence rates generally resulted in better treatment responses. Additionally, a direct correlation between the rate of photobleaching (both for cyanine and HPPH) and the tumor response was observed. Those results suggested that measuring the rate of photobleaching of HPPH conjugates in the NIR-range might be useful for light dosimetry during PDT and determining the amount of generated singlet oxygen 62.

**Figure 7:**
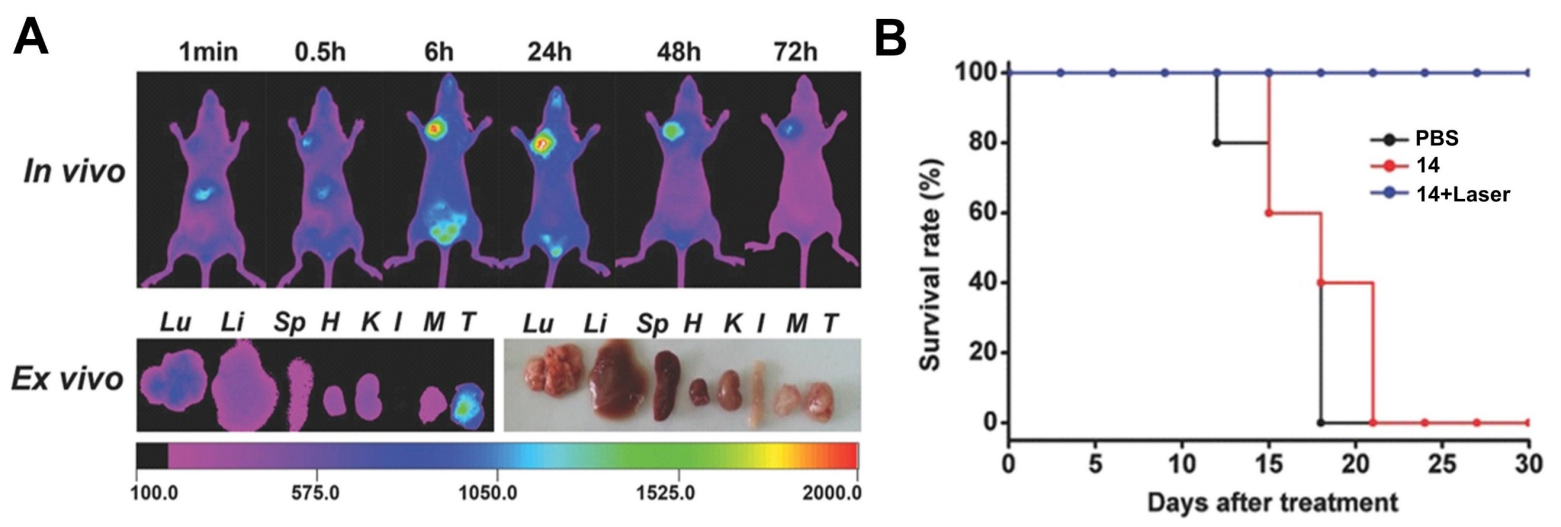
(A) Tumor preferential accumulation of compound **14** in the 4T1-tumor tissue (orthotopic tumor, mammary fat pad inferior to the nipple, close to the left shoulder) of BALB/c mice *in vivo* and* ex vivo* (T: tumor). (B) Survival Rate of xenograft mice bearing 4T1 tumors upon treatment with compound **14**. Figures reproduced with permission from reference 63. Copyright 2016 WILEY-VCH Verlag GmbH & Co. KGaA, Weinheim.

**Figure 8:**
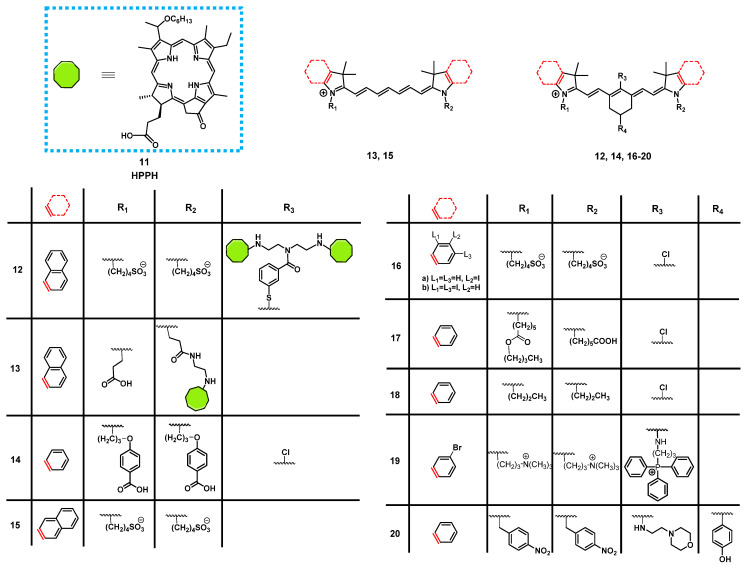
Structures of cyanine-based PSs for PDT combined with NIR-imaging 60-68.

Luo et al. investigated another series of cyanine-based PSs with various N-alkyl chains, which showed good phototherapeutic and photothermal properties as well as an increased mitochondrial uptake and retention *in vitro* due to their lipophilic, cationic characters. Compound **14** (Figure 8), which showed the best ROS generation and temperature increase for PDT and PTT, respectively, was chosen for further *in vivo* experiments (BALB/c mice, A549 and 4T1 xenografts). In the animal model, no significant toxic effects were observed, while photothermal and fluorescence imaging of compound **14** (λ_ex_ = 770 nm / λ_em_ = 830 nm) allowed the online monitoring of the tumor uptake. Hereby, compound **14** preferentially accumulated in the tumor tissue and showed a prolonged tumour retention (Figure 7A). Upon light irradiation (808 nm, 1.5 W/cm², 5 min), tumor-growth was completely inhibited, the temperature in the tumor tissue was sharply increased, and no tumor recurrence could be observed indicating a successful combined PDT and PTT treatment (Figure 7B) 63.

Another approach was investigated by Atchison et al., who designed iodinated derivatives of the NIR-cyanine dye IR-783 as a potential treatment modality for pancreatic cancer. The most active candidate from *in vitro* studies, compound **16a** (Figure 8), was investigated on SCID mice bearing ectopic BxPC3 tumors. Upon intratumoral injection (2.5 mg/kg) and light irradiation (780 nm, 100 mW, 9 min), compound **16a** caused initial tumor shrinkage, followed by a reduced growth rate. Additionally, after intravenous injection in the tail vein, the compound showed specific accumulation in the tumor tissue, which could be monitored *via* NIR-fluorescence imaging (λ_em_ = 814 nm) 64.

Tan et al. developed a unique multifunctional agent **17**, combining NIR-imaging, PDT, PTT and chemotherapeutic properties, while working on a different cyanine-based PS (Figure 8). Starting with the structure of the fluorescent dye indocyanine green (**15**, ICG) and modifying it with a cyclohexenyl ring in the middle of the polymethine-linker and two asymmetrical sidechains (Figure 8), the scientists attempted to enhance the biological activity of ICG. The asymmetrical and amphipathic structure allowed binding to protein site II of albumin to form a drug-protein complex, which primarily facilitates its preferential accumulation at tumor sites *via* the enhanced permeability and retention (EPR) effect. The released compound **17** from albumin protein was further actively taken up by cancer cells through organic-anion-transporting polypeptide transporters and accumulated in the mitochondria due to its lipophilic cationic structure. The compound showed strong emission in the NIR-range (λ_ex_ = 770 nm, λ_em_ = 830 nm), which made it possible to verify that the PS was preferentially accumulating in tumor cells *in vitro* and *in vivo* (0.25 mg/kg). Furthermore, a synergistic effect between PDT, PTT and chemotoxic properties could be observed *in vitro*, compared to the separate treatment options. During *in vivo* experiments in tumor bearing BALB/c mice (xenografts of A549, QBC-939, HeLa, and 4T1 cells), the compound (up to 20 mg/kg) induced an inhibitory effect on tumor growth. Upon NIR irradiation (808 nm, 0.5-1.5 W/cm² for 5 min), this effect was enhanced, which eventually led to tumor shrinkage. At the same time, only negligible damage was observed in other tissues 65.

In 2018, another NIR-fluorescent, cyanine-based PS **18** suitable for combined PDT and PTT was designed by Chen et al. *In vitro*, it could be shown that the compound preferentially accumulated in the mitochondria and possessed promising phototherapeutic properties. In follow-up animal studies (C57BL/6 mice, LLC xenografts), the accumulation of compound **18** after intravenous injection (0.4 mg/kg and 5 mg/kg) could be visualized in the tumors after 24 h *via* NIR luminescence (800 nm) and thermal imaging with low background signals. Upon irradiation (808 nm, 0.8 W/cm², 5 min, 24 h post injection (p.i.), an increased temperature in the tumor area (59°C) and a reduced tumor-growth rate, in combination with apoptotic and necrotic tumor tissue, could be observed 66.

Noh et al. designed a series of brominated cyanine dyes, conjugated with triphenyl phosphonium, as mitochondria targeting PSs. During *in vitro* experiments, the compounds showed promising targeting capabilities, ^1^O_2_ production upon light irradiation, as well as NIR-luminescent properties, and the most active PS **19** was chosen for follow-up animal studies. *In vivo* (BALB/c mice, NCl-H460 xenografts), compound **19** (20 nmol, intravenously) showed an amplified tumor uptake and retention, as well as a faster clearance from other organs (tumor-to-muscle-ratio = 3.94±0.44), which could be monitored *via* NIR-luminescence imaging (λ_em_ = 746 nm). After PDT treatment (662 nm, 100 mW/cm², 5 min), the tumor growth was significantly inhibited and apoptosis identified as the mechanism behind the tumor-suppression 67.

In order to target the hypoxic tumor microenvironment, Ding et al. also developed a novel cyanine-based PS **20**, with luminescence emission in the low NIR region (λ_ex_ = 640 nm / λ_em_ = 820 nm). *In vitro*, the inactive state of the compound showed no fluorescence and poor ^1^O_2_ quantum yields, while under hypoxic conditions, the nitro groups were reduced by nitro-reductase, leading to the emission of strong luminescence and increased ^1^O_2_ production upon light irradiation. *In vivo* experiments (4T1 tumor-bearing BALB/c mice) showed that compound **20** (2.5 µmol/kg) selectively induced luminescence after accumulation in the tumor tissue in the presence of nitro-reductase under hypoxic conditions. Upon irradiation (660 nm, 0.2 W/cm² for 15 min), a PDT effect was induced, which inhibited further tumor growth. The probe itself was metabolized in the liver, spleen and kidney, resulting in significant signals in those areas, but was also retained in the tumor tissue for prolonged periods of time 68.

#### 2.1.2. Hemi-cyanine photosensitizers

Using a similar approach as discussed above for **20**, Xu et al. reported the development of a novel hemi-cyanine dye **21**, as a hypoxia-activated NIR-luminescent PS. *In vivo*, the photosensitizer remained in its off-state in tumor-free control mice, while in 4T1 tumor-bearing BALB/c mice, a gradual increase in luminescence in tumor tissue demonstrated its specific accumulation in mitochondria. Furthermore, upon light irradiation at 660 nm (100 mW/cm², 20 min), the PS induced nearly a total suppression of tumor growth. This indicates that the compound is a promising NIR-luminescent PS for the detection of hypoxia, as well as for PDT 69.

In 2020, another photoactive hemi-cyanine dye **22** targeting aminopeptidase N (APN), a highly expressed enzyme in most tumor types, was developed by Zhou et al. The compound is hydrolyzed by APN in tumor cells and enters its luminescent state. *In vitro* (HepG-2, LO2 and 4T1), good solubility and stability, low dark toxicity and high ^1^O_2_ yield upon irradiation could be observed. During *in vivo* studies (BALB/c mice, bearing 4T1 tumors), it was possible to monitor the accumulation of the compound *via* its luminescence signal (700-740 nm) and upon irradiation (660 nm, 100 mW/cm², 20 min) of the tumor tissue, a strong suppression effect on tumor growth could be induced 70.

Shortly after, Chen et al. designed another hemi-cyanine photosensitizer **23** with a specific recognition sequence for γ-glutamyl-transpeptidase (GGT) and NIR-luminescence capabilities (λ_em_ = 760 nm). *In vitro*, the compound showed a negligible luminescence signal intensity and ^1^O_2_ production, unless the recognition group was specifically cleaved off in presence of GGT. *In vivo* (BALB/c mice, 4T1 tumors, 15 nmol or 30 nmol), the luminescence intensity in the tumor tissue increased over time, which allowed for tumor detection. It also showed a great inhibitory effect on the growth of transplanted tumors upon light irradiation (700 nm, 100 mW/cm^2^, 5 min), while only a weak inhibitory effect was observed in the dark 71.

**Figure 9:**
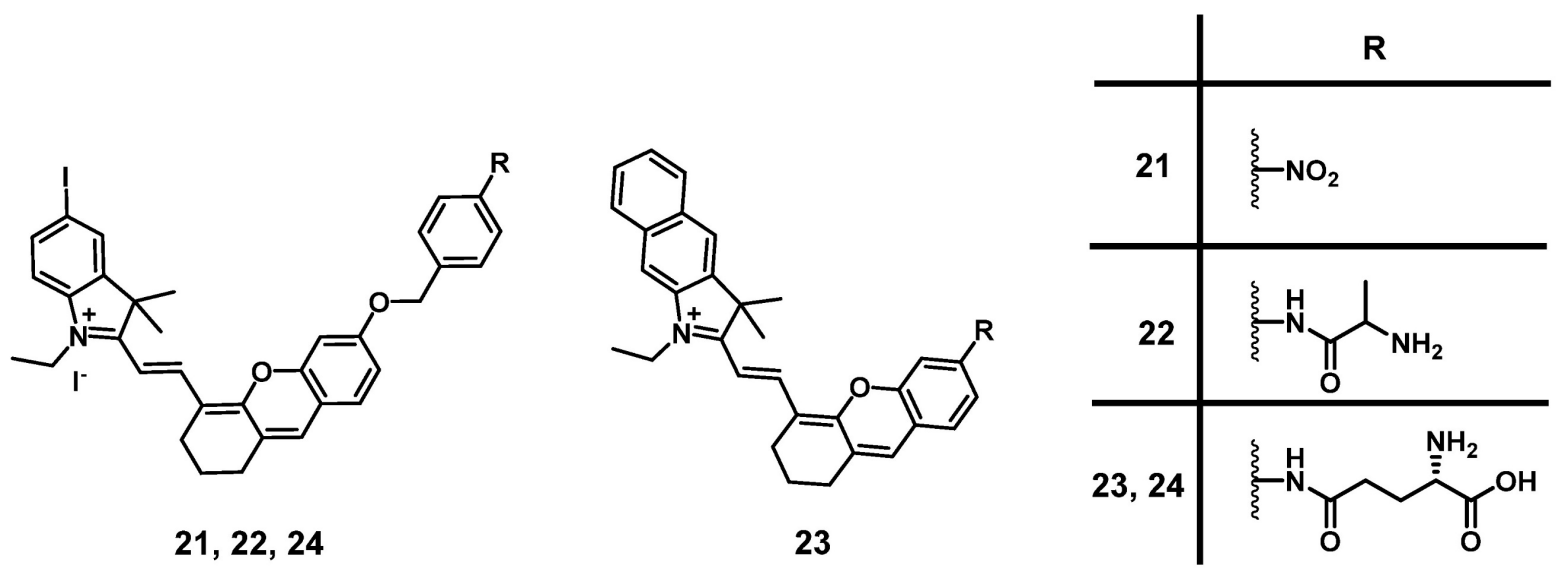
Different hemi-cyanine-based PSs for a combined theranostic approach of NIR-imaging and PDT 69-72.

A similar compound **24** (Figure 9) was developed by Liu et al. in 2022, which showed significant NIR luminescence (λ_em_ = 718 nm) and good ROS production in the presence of GGT *in vitro*, as well as a high selectivity for GGT positive tumor cells. In the following animal study (BALB/c with HepG2 xenografts), **24** (10 nmol) showed strong NIR luminescence signals upon intra-tumoral injection. Furthermore, it was possible to arrest tumor growth almost completely (20 days post PDT) *via* light irradiation (690 nm, 0.1 W/cm², 30 min) 72.

#### 2.1.3. BODIPY photosensitizers

**Figure 10:**
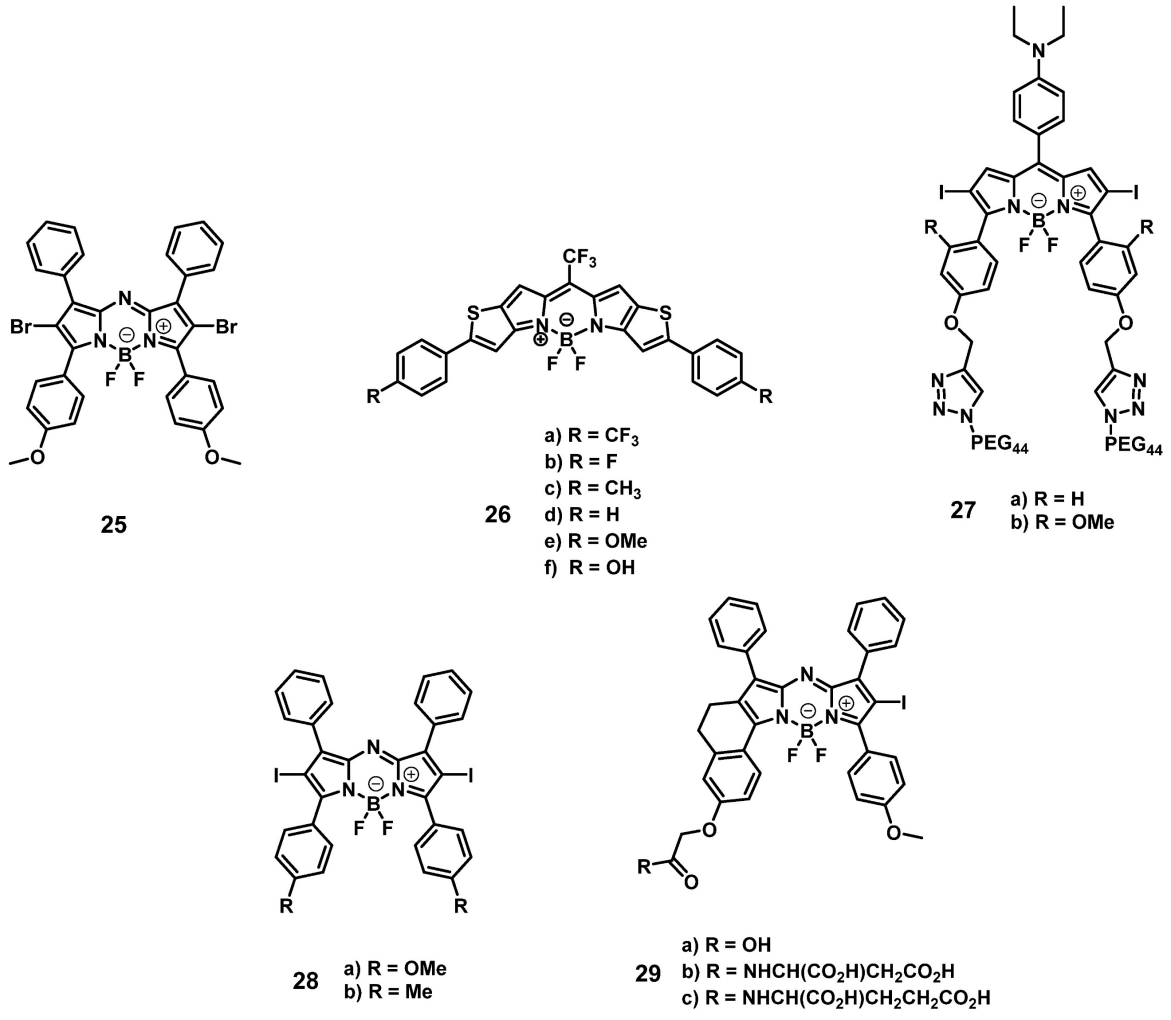
Structures of different BODIPY dyes for a combination of NIR-luminescence imaging and PDT 73-78.

Another prominent group of photoactive compounds for PDT are thieno-pyrrole fused boron-dipyrromethene dyes (BODIPYs), which have good photostability and show fast elimination from healthy tissue. The first of this class of compounds, BODIPY **25** (Figure 10), exhibiting luminescence emission in the NIR-range was developed by O'Shea and coworkers, who then investigated its tolerance and activity in several *in vivo* models (C57/BI and BALB/c nu/nu mice, LLC/1, MDA-MB-231-GFP, or MDA-MB-231-LUC xenografts). In the dark, no acute or chronic toxicity of the compound (2 mg/kg) could be observed, while luminescence imaging (λ_em_=720 nm) allowed the monitoring of its localization. Accumulation of compound **25** was also observed in the lungs, liver, kidneys, heart, and spleen, but was better retained in the tumor tissue. Upon light irradiation (670 nm or 690 nm, 50-300 J/cm², 0.7 W/cm²), no temperature increase could be observed, and a tumor cure rate of up to 71% could be induced 73.

In a follow-up study, the group investigated the mode of action of compound **25**. *In vitro*, irradiation-induced ER-stress and caspase activity, as well as ROS-triggered apoptosis could be observed in MDA-MB-231 cells. Furthermore, upon irradiation (670 nm, 150 J/cm², 0.7 W/cm²) of luciferase-expressing tumors in BALB/c mice (MDA-MB-231 xenografts, 2 mg/kg of compound **25**), a strong reduction in the luciferase-induced bioluminescence intensity, as well as a reduced cell proliferation and increased ER-stress could be observed in the tumor tissue, which indicates a prompt initiation of cell death 74.

In 2015, Watley et al. synthesized a series of thieno-pyrrole-fused BODIPY analogues, which could generate both ^1^O_2_ and luminescence in the visible-to-NIR range (688-763 nm). *In vitro*, their lead compound **26d** (Figure 10) induced no toxicity towards CT26 cells in the dark, while a significant phototoxicity could be observed upon light irradiation (690 nm, 5.6 mW/cm^2^ for 30 min). In an *in vivo* model (BALB/c mice, CT26 tumors), whole body NIR-luminescence imaging (λ_em_ = 720 nm) with the PS (5 µmol/kg) could be conducted and its accumulation in tumor tissue and skin was observed. Furthermore, after irradiation with a 690 nm diode laser (180 J/cm², 0.1 W/cm² for 30 min), the tumor volume was drastically reduced and 60 days' post-PDT, the mice were declared cured 75.

In 2018, Xiong et. al. developed two iodinated BODIPYs **27** (Figure 10) with improved solubility by coupling to PEG-chains and pH-responsive activation. The compounds showed negligible dark toxicity *in vitro*. It was observed that the PDT activity was dependent on the introduction of iodine substituents. *In vivo* (ATHYM-Foxn1^nu^ mice, MDA-MB-231 xenografts, injection of 1 mg/kg), the tumor growth could be suppressed upon irradiation (600-700 nm, 0.2 W/cm² for 30 min), while the luminescent properties made concurrent NIR-imaging possible (692 and 742 nm). Both compounds primarily accumulated in the tumor tissue followed by uptake in the liver and kidneys 76.

More recently, Yu et. al. investigated the properties of aza-BODIPY PSs. In the first study, a series of iodinated and brominated aza-BODIPYs were designed and investigated *in vitro* (Hela, MCF-7 and SW480 cells). The mono-iodinated compounds showed improved stabilities and photocytotoxicities, and as a result the mode of action and *in vivo* efficacy, of the most active BODIPY-derivative **28** (Figure 10) was further investigated. It was determined that the compound mainly accumulated in the mitochondria of Hela cells and induced apoptotic cell death upon light irradiation. *In vivo* (BALB/c mice, HeLa xenografts), the accumulation of PS **28** (2 mg/kg) could be monitored by NIR luminescence (λ_ex_ = 680 nm, λ_em_ = 720 nm), and a prolonged retention in the tumor tissue in comparison to liver, lungs, spleen and kidneys was observed. Furthermore, upon light irradiation (>590 nm, 54 J/cm², 90 mW/cm²) the tumor growth could be delayed significantly 77.

In a follow-up study, the mono-iodinated aza-BODIPYs were further conjugated with amino acids to improve their biological properties such as reduced dark toxicity and better solubility. BODIPYs combined with aspartic acid (compound **29b**, Figure 10) showed the best performance *in vitro* (A375, B16-F10, and HeLa cells), which was superior to the previously investigated analogues. In animal studies (C57BL/6 mice, B16-F10 xenografts), the accumulation of compound **29b** (2 mg/kg) could be monitored *via* NIR luminescence imaging (720 nm), which reached its maximum intensity in the tumor tissue after 12 h. The compound was retained in tumors up to 48 h and showed a slower clearance than from other organs. Upon irradiation (660 nm, 60 J/cm², 0.2 W/cm²), an improved antitumoral activity could be induced, which led to 40% total tumor shrinkage 78.

#### 2.1.4. Bacteriochlorine and N-hydroxysuccinimide photosensitizers

**Figure 11:**
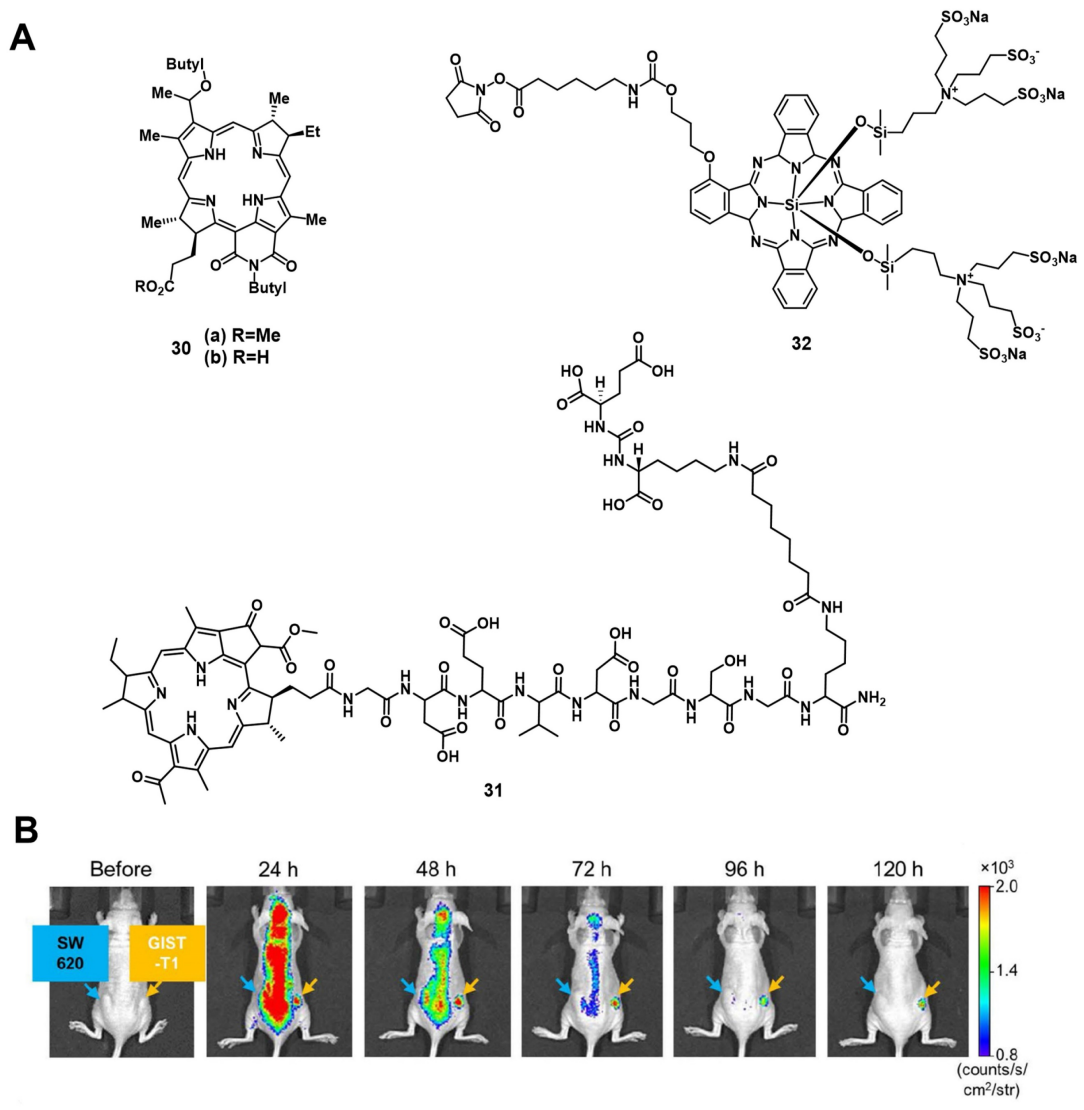
(A) Theranostic agents containing bacteriochlorin or phthalocyanine analogues for a combined approach of NIR and PDT 79-81; (B) IR image of the tumor accumulation, of the photosensitizer-antibody conjugate in GIST vs. SW tumors. Reproduced with permission from ref. 82 Copyright 2018 Ivyspring International Publisher.

Patel et al. synthesized two NIR-emissive bacteriochlorin analogues **30a** and **30b** (Figure 11) to combine luminescence imaging and PDT. All investigated compounds showed strong luminescence suitable for *in vitro* and *in vivo* imaging (λ_ex_ = 671-705 nm, λ_em_ = 750 nm). *In vitro*, the best uptake and PDT effect could be observed for the compound **30b** with a carboxylic acid group. However, in the *in vivo* tumor model (BALB/cAnNCr mice, CT26 tumors, intravenous injection of 0.25 µmol/kg), the results were inversed. Compound **30a** bearing a methylester showed a better uptake and tumor volume reduction upon light irradiation (787 nm, 135 J/cm², 75 mW/cm²), as well as a negligible skin phototoxicity 79.

In 2020, Overchuk et al. developed a targeted, bacteriochlorophyll-based NIR-emissive PS (BPP, **31**, Figure 11). The designed compound consisted of a PSMA ligand, a peptide linker and the PS itself, and was based on previous work on pyropheophorbide-peptide-PSMA PSs with long blood circulation time (see chapter nuclear imaging). *In vivo* (athymic nude mice, PC3-pip and PC3-flu xenograft, iv. injection of 50 nmol), the compound showed a long plasma-circulation time and specific accumulation in PSMA-positive tumors (PC3-pip), which could already be visualized 1 h p.i. by NIR-luminescence (λ_ex_ = 704 nm / λ_em_ = 745 nm). Upon PDT treatment (750 nm, 125 J/cm², 70 mW/cm²), a reduced tumor growth and inflammation at the tumor site could be observed. Further *ex-vivo* biodistribution analysis showed an excellent tumor targeting and organ sparing effect on the surrounding tissue (tumor-to-muscle = 3.8±1.7 / prostate-to-muscle = 0.73±0.54) 80.

In 2011, Mitsunaga et al. conjugated an N-hydroxysuccinimide (NHS) ester of the phthalocyanine dye IR700 **32** to monoclonal antibodies (Trastuzumab & Panitumumab) for targeting epidermal growth factor receptors *via* immune-targeted, image-guided PDT. *In vitro* (3T3-HER2 cells), no significant toxicity in the dark could be observed, while irradiation with light (fluorescence microscope, 2.2 mW/cm^2^) induced a strong PDT effect. During *in vivo* studies (mice with A431 or 3T-HER2 xenografts), the specific accumulation of the antibody conjugate (50 or 300 µg iv. injection) in the tumor tissue could be monitored *via* visible-to-NIR luminescence imaging (λ_ex_ = 590-650 nm, λ_em_ = 665-740 nm), and upon light irradiation (50 J/cm²), the tumor volume was significantly reduced 81.

A few years later, Fujimoto et al. reported the conjugation of the PS IR700 (**32**) to the anti-c-KIT antibody, which targets the transmembrane tyrosine kinase CD117 in gastrointestinal stromal tumors (GISTs). *In vitro*, *ex vivo* and *in vivo* (BALB/c nude mice bearing GIST-T1 tumors and SW620 tumors as a negative control), after administration of the compound (*in vivo* 100 µg per iv.), the specific accumulation in gastrointestinal stromal tumors could be observed *via* NIR luminescence imaging (λ_ex_ = 675 nm, λ_em_ = 720 nm) as shown in Figure 11. Upon NIR light irradiation (685 nm, 100 J/cm^2^, 55.5 mW/cm², 3x per week), all treated tumors showed regression and more than half disappeared completely within 30 days post PDT 82.

#### 2.1.5. Rhodamine and protoporphyrin IX photosensitizers

**Figure 12:**
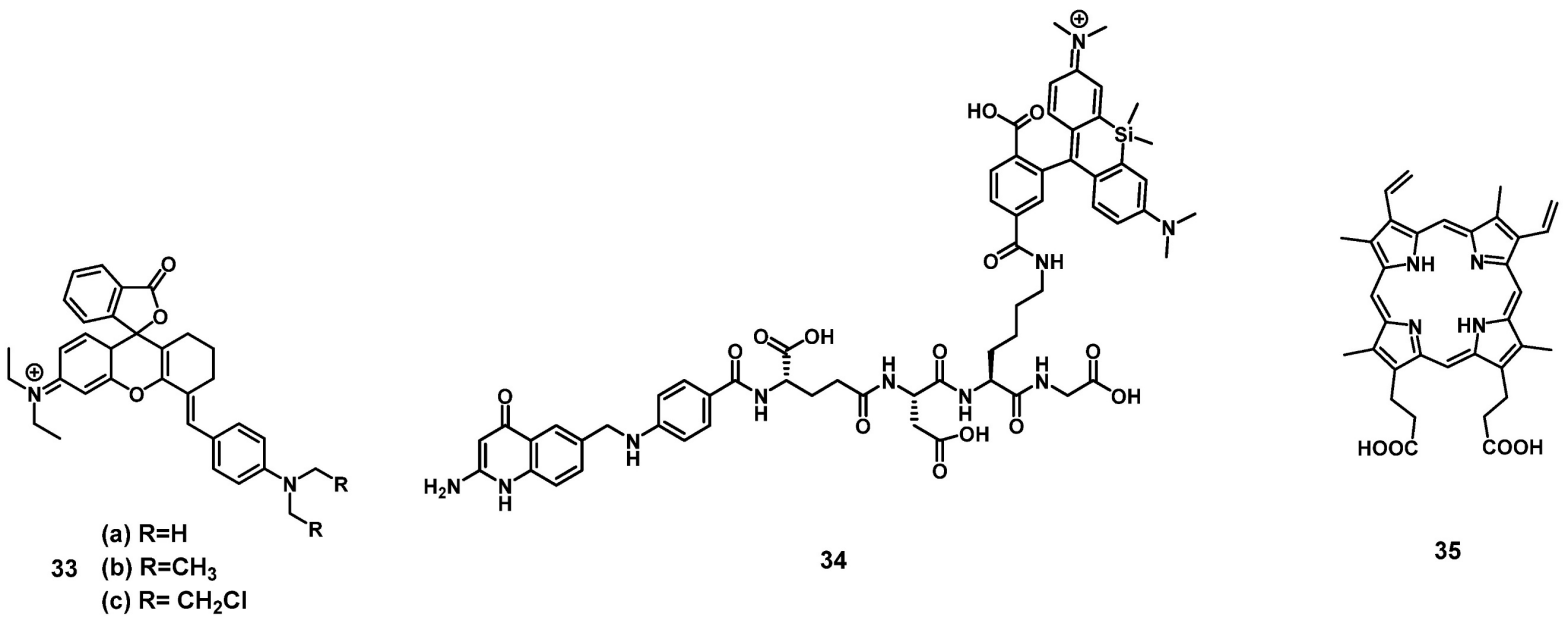
Theranostic agents containing rhodamine, and the photosensitizer protoporphyrin IX for a combined approach of NIR-imaging and PDT 83-85.

In 2019, a series of three rhodamine-based multimodality agents **33** (Figure 12) were designed by Qu et al, which combined NIR-luminescence imaging with both PDT and chemotherapy. The compounds showed strong luminescence induction at 716 nm, together with limited toxicity in the dark and promising mitochondrial targeting *in vitro* (HepG2 and B16F10). However, compound **33a** showed only negligible phototoxicity. The two other compounds **33b** and **33c** were further investigated *in vivo* (C57 mice, bearing B16F10 allografts) using both intra-tumoral (2 µmol/kg) and intravenous (1 µmol/kg) injections, where they enabled NIR-imaging (λ_em_=716 and 750 nm) of the tumor tissue. The compounds showed low systemic toxicity, in combination with a good bioavailability and significant suppression of tumor growth (intra-tumoral: up to 74.6%; intravenous: up to 48.5%). Upon irradiation of the tumor tissue (660 nm, 200 mW/cm², 15 min) this suppression effect on tumor growth could be increased significantly (intra-tumoral: up to 93.9%; intravenous: up to 90.2%) 83.

Another rhodamine-based PS was developed by Aung et al., who coupled Si-rhodamine through a peptide linker to folic acid to enable specific targeting of folate receptor positive tumors. *In vivo* (BALB/cAJcI-nu/nu mice, OVCAR-3, KB and A-4 xenografts), compound **34** (10 nmol, iv. injection) showed increased accumulation as well as prolonged retention (λ_em_=690-740 nm) in folate-receptor positive tumors (KB)in comparison to folate receptor negative organs and tissue. Upon irradiation (635 nm, 50 J/cm², 0.25 W/cm²), tumor growth could be significantly delayed, and a reduction of proliferation markers was observed, but for a complete removal of tumor mass, further irradiations would have been necessary 84.

In 2020, Garcia et al. developed a system to monitor PDT treatment of skin cancer, using luminescence imaging in the red-to-near infrared range (600-750 nm) with protoporphyrin IX (**35**, Figure 12). It was reported that the red excitation light for PDT (407 nm, 120 J/cm², 50 mW/cm²) could hereby be used directly for imaging *in vivo* (nude mice, A431 xenograft), which generated a substantial fluorescence signal throughout the lesions and allowed for a deeper tissue penetration, as well as continuous monitoring of the skin lesions 85.

#### 2.1.6. Triphenylamine photosensitizers

**Figure 13:**
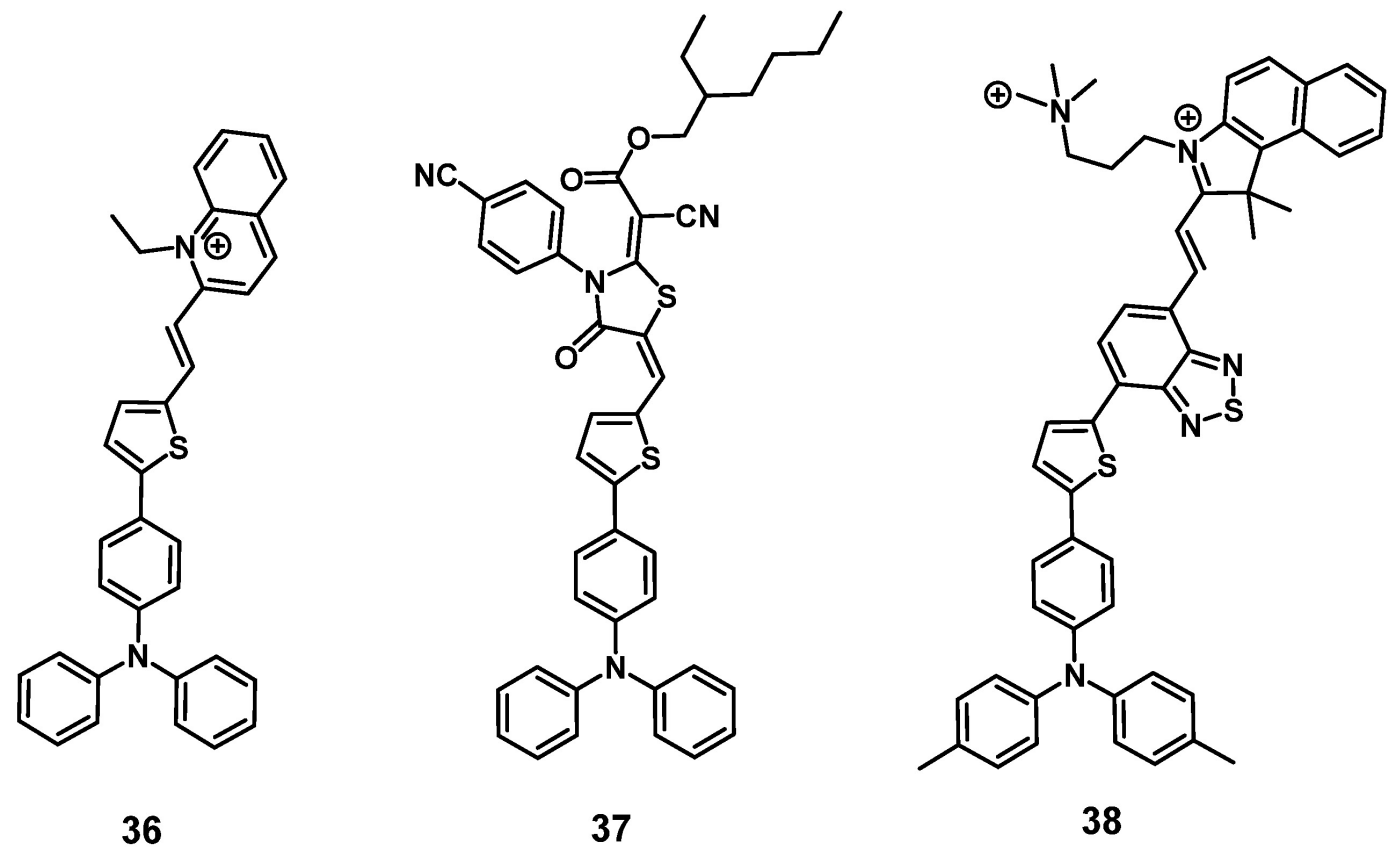
Structures of the triphenylamine based NIR-luminescent PSs 86-88.

In 2021, Qi et al. designed a cationic luminescent probe **36** (Figure 13) containing a triphenylamin- and a quinolinium moiety for imaging dynamic changes in mitochondrial proteins, which fulfill essential roles in cellular mechanisms. The compound showed luminescence in the visible-to-NIR range and was capable of effectively detecting dynamic changes of the mitochondrial proteins *in vitro* (HeLa) that were induced by the drugs 10-hydroxycamptothecin (HCPT), epirubicin (Epi) and cyclophosphamide (CPA), which are used or investigated for the treatment of myelodysplastic syndrome, metastases and lymphomas respectively. Furthermore, compound **36** also showed an excellent capability to produce ROS *in vitro* and was successfully used to monitor its tumor accumulation in Hela tumor-bearing mice *via* luminescence imaging (λ_ex_ = 543 nm, λ_em_ = 600-750 nm) 86.

In the same year, the development of a similar NIR-activatable and NIR-emissive PS **37** with a donor-π-acceptor structure based on triphenylamine, thiophenyl and rhodanic for imaging-guided PDT was reported by Chen et al. During *in vivo* imaging of 4T1 tumor-bearing mice, a bright visible-to-NIR signal (λ_ex_ = 1100 nm, λ_em_ = 600~700 nm) could be detected at the tumor site for 48 h after intra-tumoral injection (200 µg). In addition, tumor growth inhibition could be observed upon light irradiation (white light, 100 mW/cm^2^, 8 min), and no significant damage to other organs was detected 87.

Gu et al. also reported the synthesis of a series of compounds including a donor-π-acceptor structure based on trimethyl-benzindoles and triphenylamines, as PSs for NIR-II luminescence and photoacoustic imaging guided PDT/PTT. *In vitro* (MCF-7 and 3T3 cells), the compounds displayed significant NIR luminescence, a good PDT effect, as well as satisfactory mitochondria targeting properties in the tumor cells. Through the addition of benzothiadiazole, the push-pull effect was enhanced in **38** (Figure 13), caused a stronger red-shift of its luminescence into the NIR-II range, and also induced excellent PDT, as well as PTT properties. Thus, this compound was further investigated *in vivo* (BALB/c mice, 4T1 allografts) through iv. injection into the tail vein (100 µg). Hereby, the accumulation of the compound in tumor and liver was determined *via* luminescence (λ_ex_ = 800 nm, λ_em_ = 1050 nm) and photoacoustic imaging. Upon light irradiation (671 nm, 0.4 W/cm², 10 min), an almost complete elimination of the tumors could be achieved, while no major damage to other organs and tissue was observed 88.

#### 2.1.7. NIR/PDT utilizing viscosity sensitive probes

**Figure 14:**
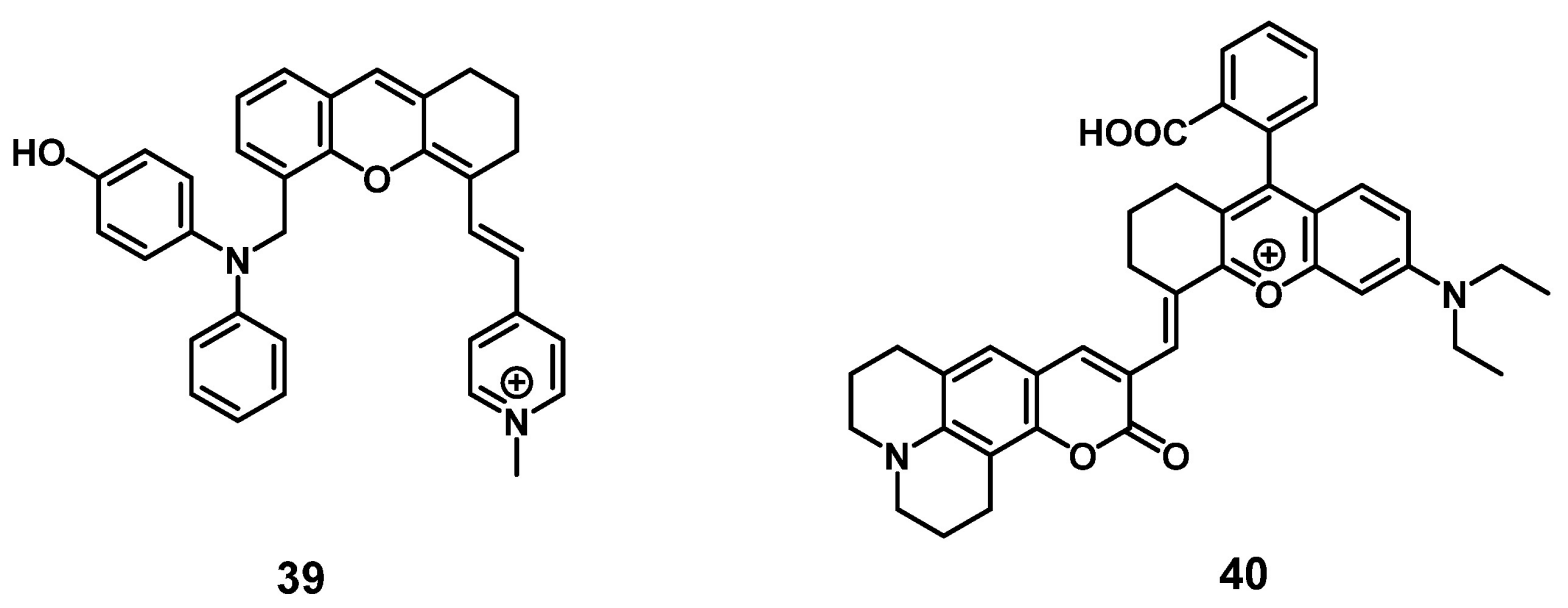
Theranostic viscosity probes with NIR-luminescence and PDT activity 89, 90.

Very recently, a mitochondria-targeted, viscosity-sensitive, NIR-luminescent PS based on enhanced luminescence in highly viscous environment, where the rotation of the present phenyl groups is limited, was reported by Fan et al. Since abnormal viscosities in the mitochondria are indicators of various diseases such as inflammation or cancer, compound **39** (Figure 14) would be a promising theranostic agent. *In vitro* (HeLa cells), the compound exhibited a high efficiency for PDT and ROS generation upon light irradiation, but also demonstrated good mitochondria targeting capabilities as well as ability for real-time monitoring of viscosity changes *via* luminescence in the visible-to-NIR range. In the xenograft HeLa tumor-bearing mice, the probe enabled the visualization of inflammation, tumors, as well as fatty liver tissue through luminescence imaging (λ_ex_ = 560 nm, λ_em_ = 720 nm) and also induced tumor shrinkage (up to 25%) upon light irradiation (635 nm, 100 mW/cm², 30 min) 89.

Similarly, Li et al. designed a NIR-luminescent, coumarin-based viscosity imaging probe **40** (Figure 14) responsive for changes in the wound microenvironment and therefore suitable for antimicrobial PDT. Bacteria often increase the viscosity of their surroundings due to a higher protein content and through this, they can develop tolerance against drugs. *In vitro*, the compound showed luminescence signals upon incubation on the gram-positive bacterium *S. aureus*, and significant phototoxicity upon irradiation, but no luminescence could be observed on gram negative bacteria (*E. coli*). In a mouse model (mice with *S. aureus* infection, 0.5 mM of **40** iv.), the compound showed accumulation and strong luminescence (λ_ex_ = 633 nm, λ_em_ = 750-800 nm) in the area infected with the bacteria and triggered PDT under laser irradiation (690 nm) 90.

#### 2.1.8. NIR/PDT utilizing FRET or BRET

**Figure 15:**
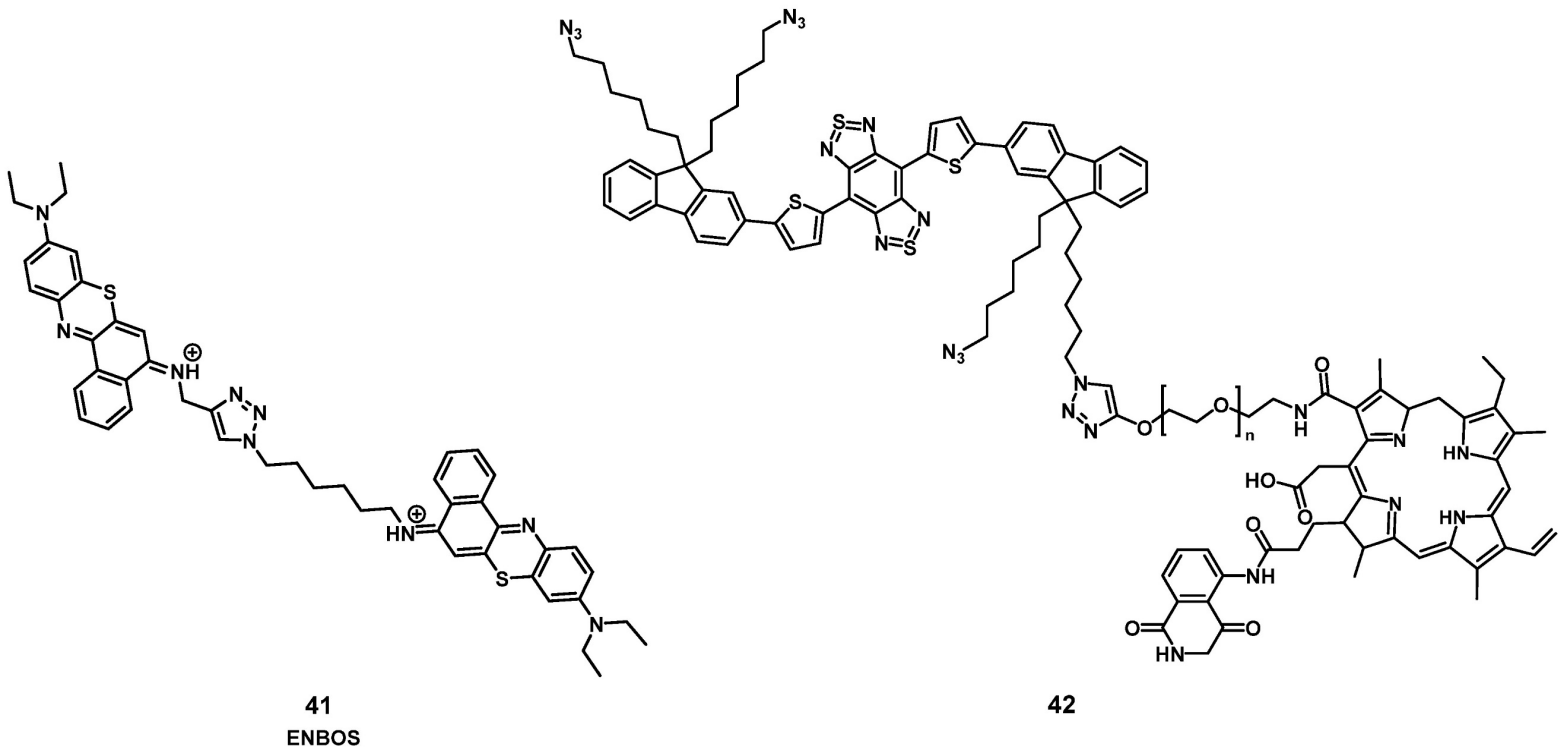
PDT photosensitizers with NIR-imaging capabilities, which utilize BRET or FRET 91, 92.

In 2019, Li et al. designed a photon-initiated dyad cationic superoxide radical generator **41** (ENBOS, Figure 15) to produce ^1^O_2_ by combining a NIR-active fluorophore with benzophenothiazines. Upon light irradiation, this compound induces a Förster resonance energy transfer (FRET), successfully endowed significantly enhanced NIR absorbance and photon utility, which in turn lead to it more easily activated and generating more O_2_^-.^ in deep tissues, thus dramatically intensifies the Type I PDT against hypoxic deep tumors. *In vitro* (4T1 cells) compound **41** showed negligible dark toxicity as well as amplified ROS generation. *In vivo* (BALB/c mice, 4T1 xenografts), an improved intra-tumoral accumulation was observed, which could be monitored by NIR luminescence (655-755 nm) up to 120 h p.i. into the tail vein (16 nmol). Furthermore, upon irradiation (660 nm, 0.1 W/cm², 15 min) significant tumor growth inhibition could be achieved even in deeper seated tumors (68% inhibition through 5 mm tissue) 91.

Recently, Yuan et al. reported the development of a self-luminous molecule based on bioluminescence resonance energy transfer (BRET). Compound **42** (Figure 15) contained a bioluminescent molecule (luminol) in addition to the PS chlorin e6, a PEG chain, and a NIR-II luminescent dye. Under normal conditions, **42** should show luminescence emission, while in the presence of increased amounts of ROS, the bioluminescent molecule initiates BRET, which activates the PS and leads to ^1^O_2_ production. ^1^O_2_ in turn oxidizes the thiophene structure, which results in a NIR-II bioluminescence signal. After the BRET-activation, as well as the subsequent imaging and treatment capabilities had been verified *in vitro* (A549 and L02 cells), additional studies in *in vivo* models were conducted. Upon injection (1 mg) into inflamed joints (SD rats, inflammation induced through papain), a strong NIR-II bioluminescence signal (1100 nm) could be detected while no signal was observed in healthy animals. When compound **42** was administered intravenously (0.5 mg) to tumor-bearing mice (BALB/c mice, MC38 xenografts) and irradiated with light (660 nm, 1 W, 1 min), a self-luminescent signal could be observed in the tumor area. Afterwards, the luminescence gradually decreased. Application of BRET represents an alternative way for monitoring the PDT efficacy and imaging the localization of inflammations *in vivo* without the use of external light irradiation 92.

### 2.2. Metal-based photosensitizers

Besides organic compounds, the use of transition metal complexes as PSs has significantly increased over the last decade. Transition metal complexes are usually relatively photo-stable compared to purely organic compounds, which would reduce the necessary dose applied for treatments. Photoactive metal-based compounds often also show a bathochromic shift compared to the non-metallated photosensitizers due to favourable metal-ligand interactions and heavy metal effect. Metal-based systems can also be modified more easily for tuning their absorption/emission properties towards the phototherapeutic window. Furthermore, the addition of metals promotes a more efficient intersystem crossing towards the excited triplet state and increases its life-time, which results in a higher production of ROS (Figure 1) 57, 93-95. The most prevalent transition-metal based PSs are complexes based on ruthenium (Ru) or iridium (Ir), since both metals can often be excited *via* two-photon irradiation in the phototherapeutic window (650-900 nm) and thus, allowing for deeper tissue penetration leading to increased generation of singlet oxygen. Ruthenium complexes often also have a pronounced photo-stability, while octahedral iridium complexes are intrinsically luminescent and often have a preference for Type-I PDT 93, 94. Besides Ru and Ir, several other metals like osmium (Os), palladium (Pd), rhenium (Re), zinc (Zn) and various lanthanides have also been investigated as components of PSs due to their high luminescence quantum yields or efficient singlet oxygen production. However, most of these metals are currently still undergoing *in vitro* evaluation for PDT applications and have not reached the stage of animal studies 95, 96.

#### 2.2.1. Zn-based NIR Photosensitizers

**Figure 16:**
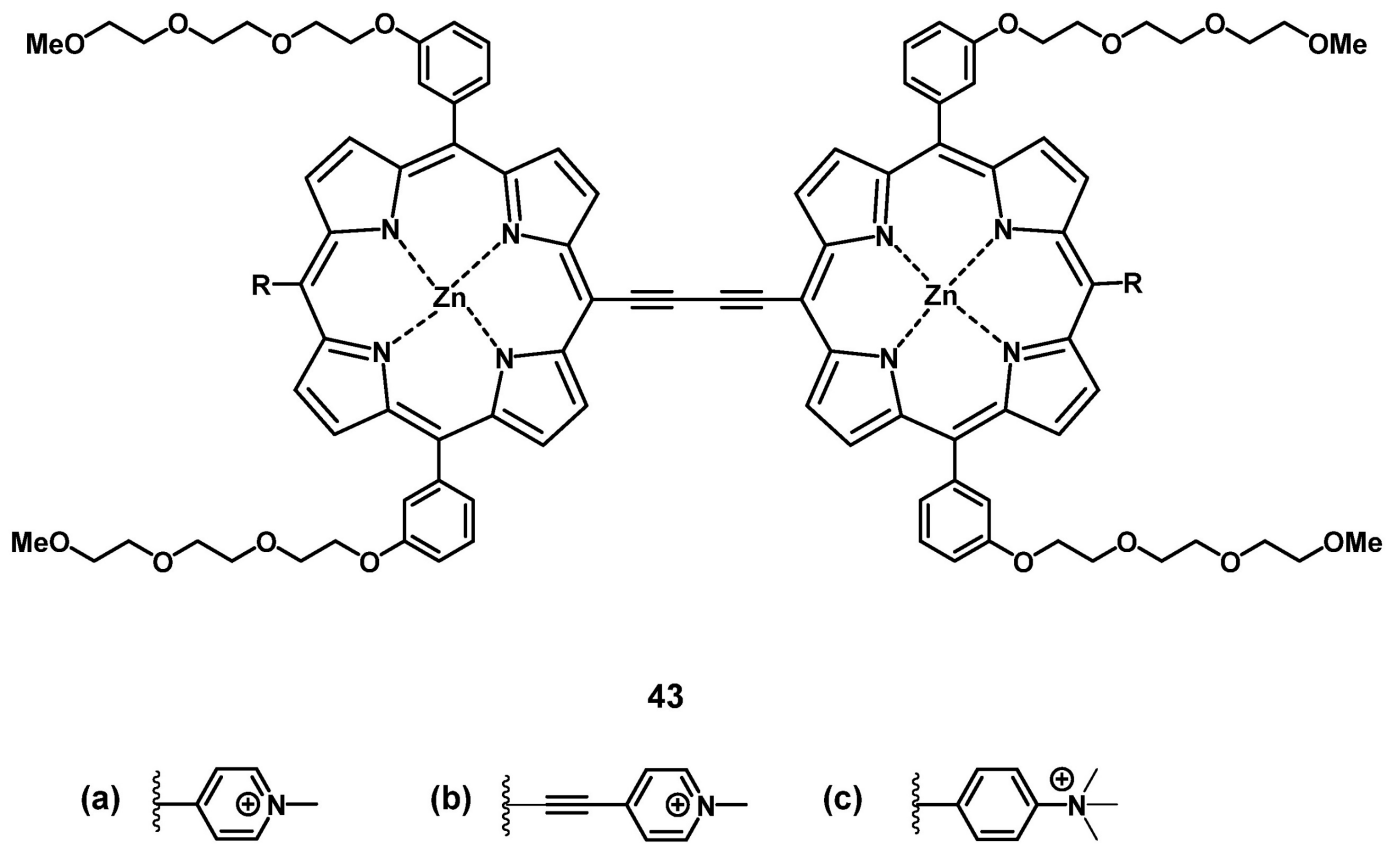
Structure of oxdime, a dimeric Zn-porphyrin PS, with luminescence in the NIR range 99.

The group of Anderson investigated the photo-physical and photo-biological properties of different dimeric porphyrin-based PSs with zinc (e.g. compound **43**, Figure 16). *In vitro* (SKOV-3 cells), the compounds showed negligible dark toxicity, but significant cytotoxicity upon irradiation *via* either one-photon (657 nm) or two-photon (920 nm) excitation 97. In a complementary study, Collins et al. developed a series of anionic- and cationic Zn-porphyrin-dimers, and investigated their capability to induce selective blood vessel closure upon two-photon excitation for PDT. This would allow for an indirect treatment of tumors, by targeting their increased blood vessel proliferation. *In vitro*, all compounds showed luminescence in the NIR range, and the cationic complexes also exhibited significant PDT activity. The ability of the most active compound **43b** to close blood vessels upon two-photon irradiation (920 nm, 39 mW, 15 min) was demonstrated *in vivo* (NCRNU-M mice, B16-F10 allograft) using a dorsal skin-fold window chamber model while the PDT-progress was monitored using (NIR) fluorescence microscopy (λ_em_ = 690-940 nm).^98^ Dimer **43b** was later investigated for its pharmacokinetics by biodistribution studies and NIR-luminescence imaging *in vivo*. The selectivity of **43b** for melanoma tumors in SCID mice bearing B16-F10 allografts was comparable to the approved PS Photofrin (Figure 2), but showed generally a faster uptake and clearance rate in all tissues. Accumulation in the tumor tissue peaked 3-12 h post-injection (tumor-to-skin ratios up to 3.8) which was significantly faster than Photofrin (24 h p.i.). In the course of the study, it was observed that the main luminescence signal of the compound shifted from 800 nm to 740 nm over a duration of 24 h, which is likely due to its metabolization 99.

#### 2.2.2. Gd-based NIR Photosensitizers

**Figure 17:**
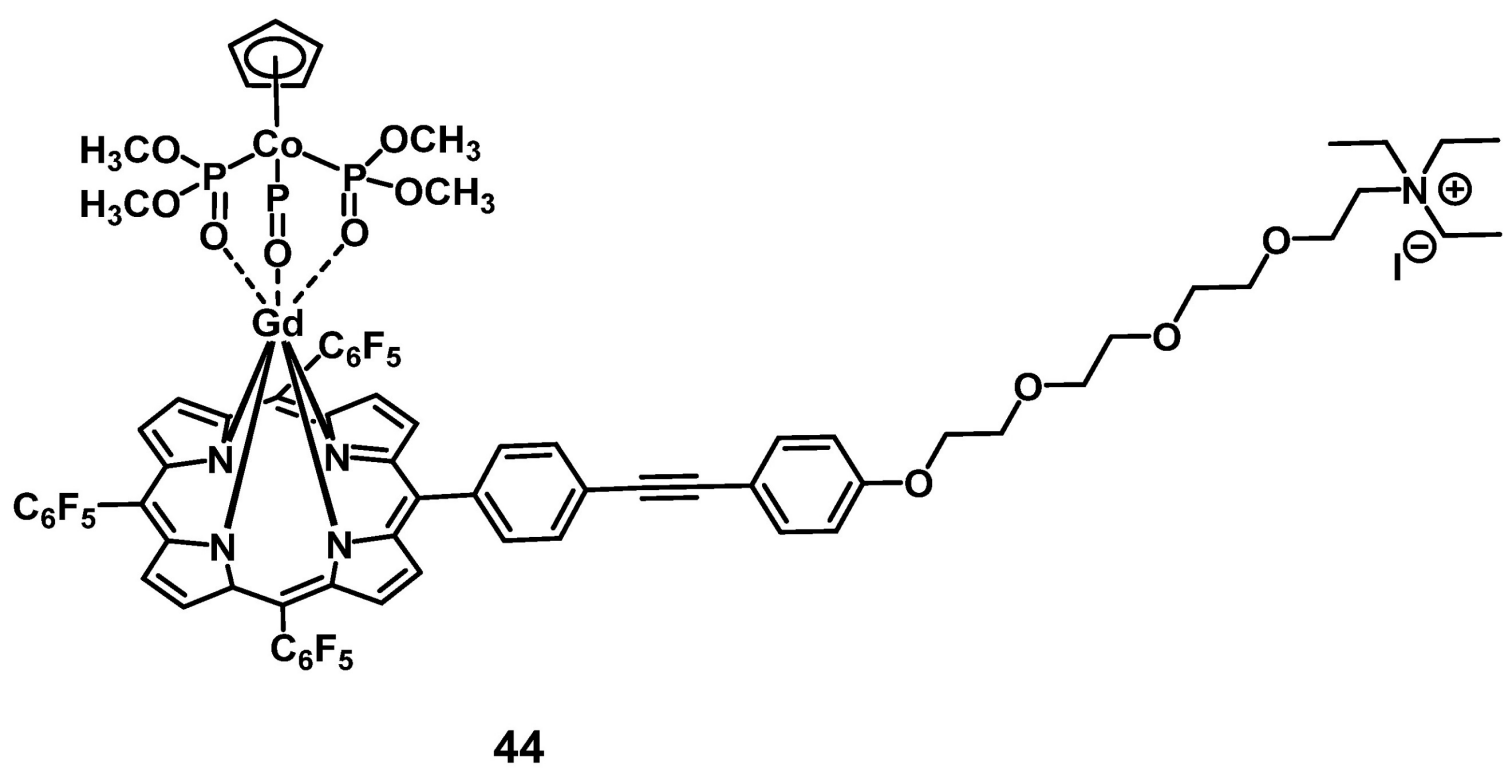
Cancer cell membrane targeting Gd-porphyrin PS 100.

In 2014, Zhang et al. developed a gadolinium-porphyrin complex **44** (Figure 17) as a tumor selective PS by means of targeting the membrane of cancer cells *via* an affinity towards certain anionic membrane-phospholipids, like phosphatidylserine, which are more common in cancer than in healthy tissue. Compound **44** was found to exhibit two-photon-induced visible-to-NIR luminescence emissions (~650 and ~750 nm). *In vitro* (HeLa, MRC5 cells), the complex showed low dark toxicity, high cancer cell specificity and membrane binding, due to its affinity for those phospholipid species, as well as promising phototoxic effects. Meanwhile its ytterbium (Yb) analogue and the Gd-complex modified with an additional rhodamine-moiety, a mitochondria-targeting vector, were both shown to also accumulate in the cancer cells, but had either no PDT effect (Yb-complex) or no cancer specificity (Gd-rhodamine). Further *in vivo* studies in a mouse model (BALB/c mice, HeLa xenografts) showed that complex **44** (1 µmol/kg, intravenous injection) showed detectable fluorescence signals and induced a significant reduction of the tumor volume (by 50%) upon light irradiation (860 nm, 50 J/cm²) 100.

#### 2.2.3. Ir-based NIR Photosensitizers

Wang et al. developed a series of cyclometalated iridium complexes with benzimidazole derivatives as ligands. The compounds showed red phosphorescence and upon extension of the aromatic ring structure a significant red-shift into the NIR range could be observed. During *in vitro* studies (HeLa, A549, A549R, HUVEC cells), the compounds were capable of inhibiting migration and invasion upon irradiation with short wavelength light and showed no significant dark toxicity. The effect of the complexes increased in acidic conditions, which indicates potential application for cancer imaging based on the acidic tumor environment. In addition, three kinases were identified as possible molecular targets by a kinase-inhibition-screening assay. In a zebrafish model, the localization of all compounds could be found in the notochord, intestine and spinal cords determined *via* visible-to-NIR luminescence imaging, with compound **45** showing the farthest red-shift (λ_em_ >700 nm), and complexes **45** and** 46** being capable of inhibiting angiogenesis. The two compounds were then further investigated for PDT application in nude mice (BALB/c) with HeLa-xenografts. Both compounds (5 mg/kg, administered intratumorally at day 0 and day 7) effectively inhibited tumor growth upon irradiation (430 nm, 360 mW/cm², 300 s, 1 h p.i.) and were capable of reducing tumor volume. The PS with the more extended aromatic structure showed a stronger PDT effect in comparison to the compound without an extended aromatic system (reduction of tumor volume by 74.5 ± 8.3%). This study showed the potential of dual-functional iridium complexes as possible theranostic agents 101.

#### 2.2.4. Ru(Pt)-based NIR Photosensitizers

**Figure 19:**
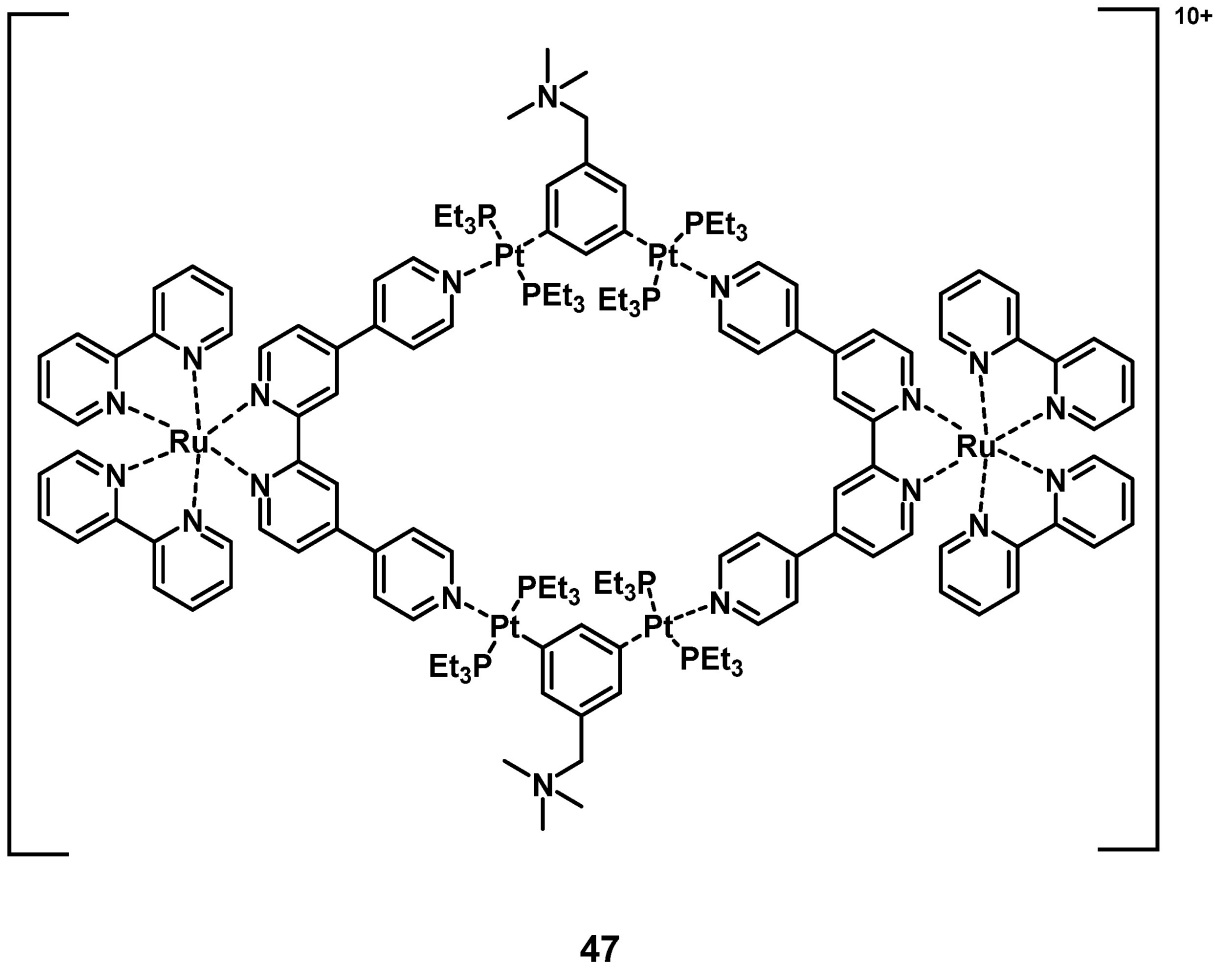
Structure of a Ru-Pt metallacycle with NIR luminescence emissions and PDT activity 102.

In 2018, a heterometallic ruthenium-platinum polypyridyl metallacycle (**47**, Figure 19) was synthesized *via* coordination-driven self-assembly. The compound showed two-photon absorption at 780 nm and 880 nm, which allowed for deeper tissue penetration. The interaction between the metal centers induced a red-shift of its luminescence towards the NIR-region (λ_max_ = 696 nm) and induced a higher efficiency for the generation of singlet oxygen. During *in vitro* studies (A549 cells), the complex showed only weak dark toxicity and its large, positively charged macrocycle-structure facilitated cell internalization after which the compound accumulated in the mitochondria and nucleus. In the following *in vivo* study (BALB/c mice, A549 xenografts), the complex showed minimal systemic toxicity as well as an efficient tumor treatment (tumor size reduction of 78% after 14 days) upon light irradiation (800 nm, 50 mW, 20 s/mm, conducted on day 0 and day 7) 102.

**Figure 20:**
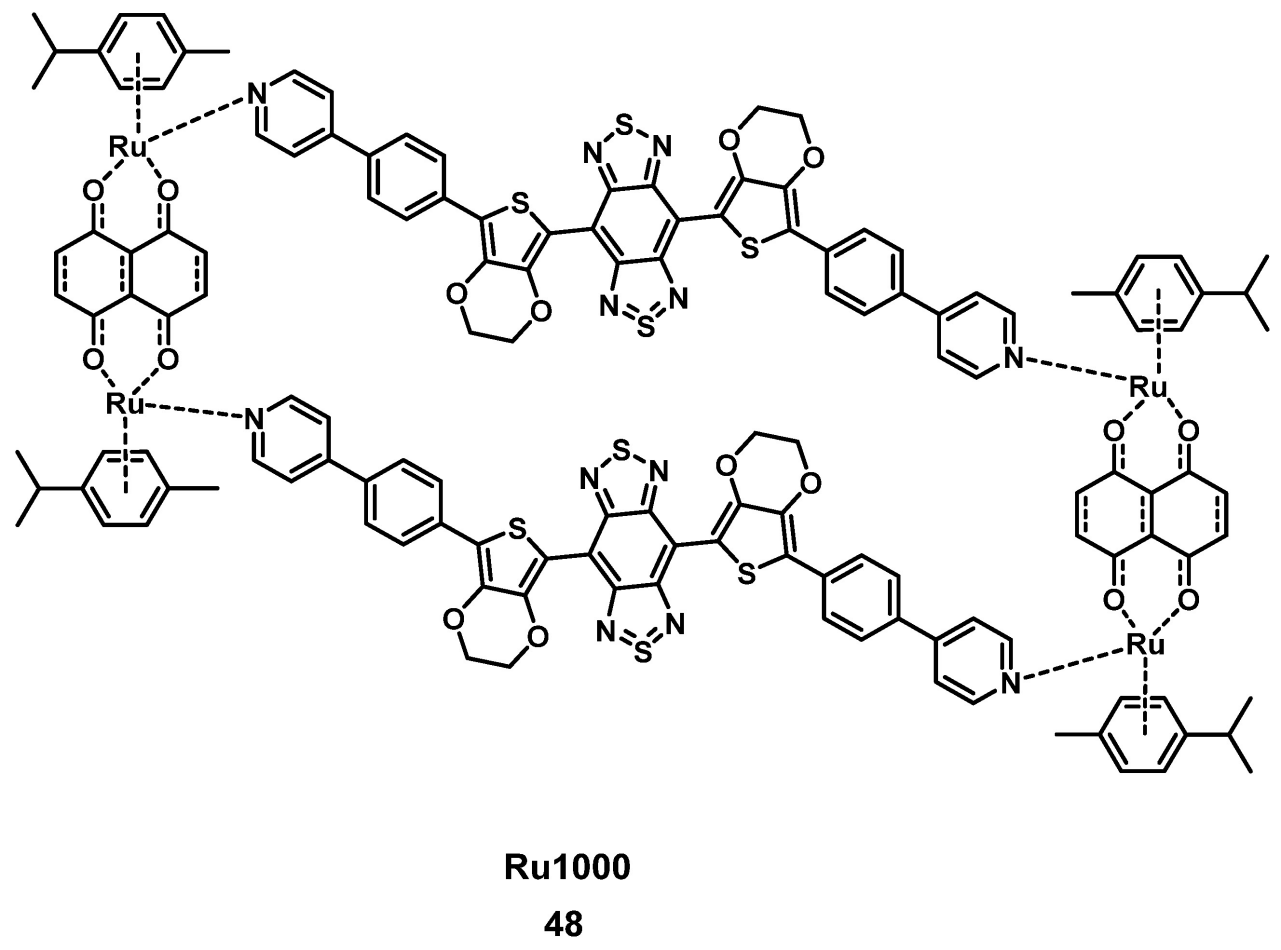
Structure of the NIR-II emissive, NIR-I-activatable PS Ru1000 103.

More recently, Fan et al. developed another ruthenium complex **48**, termed Ru1000 (Figure 20). The complex had an optimized donor-acceptor-donor scaffold (3,4-ethylenedioxythiophene in combination with benzo-bisthiadiazole), contained multiple phenylpyridine coordination units and could be formed *via* coordination-driven self-assembly. Compound **48** showed a high luminescence yield in the NIR-II range (~1000 nm), which is suitable for the imaging of deeper-seated tumors upon irradiation with NIR light. *In vitro*, the complex showed good cellular uptake, anti-metastatic properties and cancer selectivity in combination with an enhanced cytotoxic effect in comparison to cisplatin against various cancer cell lines (A549, HeLa, HepG2, 16HBE) after photo-activation. During *in vivo* studies (BALB/c mice with A549 tumors), monitoring of the uptake of Ru1000 in the tumor tissue could be achieved by NIR-II imaging and a slight inhibition of tumor growth in the dark could be observed. Upon irradiation (808 nm, 1 W/cm², 5 min), the complex induced ROS-production, a significant temperature increase (up to 52.6°C), and shrinkage of the tumor volume after intratumoral injection (1 mg Ru/kg), which finally resulted in their complete elimination, while no relapse was observed for 16 days 103.

Those results show that by combining NIR optical imaging with PDT, either with metal-based or metal-free compounds, has great potential for clinical applications since it makes the therapy and imaging of deeper-seated tissues possible. The combination of the two modalities improves the monitoring of PS treatment by imaging, which underlines its potential for a theranostic approach useful in a clinical setting.

## 3. MRI and PDT

Magnetic resonance imaging (MRI) has been acknowledged as a remarkable non-invasive diagnostic technology, which comes with the advantage of high spatial and time resolution 104. Hereby, the signal contrast in MRI is a result of distinct magnetic properties of protons in different tissues, as well as the varying densities of water molecules therein. This technology already plays a crucial role in the detection of tumors, the development of new drugs, as well as during therapy monitoring by providing information about location and size of lesions non-invasively. However, the differences of contradistinction between healthy and diseased tissues can be quite limited in some cases, which then necessitates the use of contrast agents 105, 106. These contrast agents (CAs) used in MRI enhance the diagnostic accuracy by modifying the relaxation characteristics of water protons in local tissues. It operates by reducing the longitudinal relaxation time (T1) or transverse relaxation time (T2) of protons, which increases the signal intensity in the corresponding weighted phase resulting in a significant MR signal enhancement. As a result, contrast agents can be separated into T1-based and T2-based agents 107, 108. Gadolinium (Gd) complexes, as a highly paramagnetic lanthanide-series heavy metal, belongs to an especially auspicious family in this pool of CAs for MRI. Though Gd(III) is inherently toxic, complexes formed by chelation with different ligands can provide Gd-complexes of high thermodynamic and kinetic stability, thereby reducing the cytotoxicity of the metal 109, 110. To date, eight different Gd(III)-based CAs have been clinically approved for MRI 111. However, a common issue of such CAs is that they can potentially induce nephrogenic systemic fibrosis due to their Gd(III) cores. Because of this, for individuals with varied degrees of acute or chronic renal insufficiency, the use of CAs is avoided or limited during MRI 112. Therefore, a number of strategies to improve the stability of the CAs, while maintaining or enhancing their relaxivity have been investigated.

The combination of PDT and MRI offers a promising approach to improve the efficiency of both technologies *via* theranostics, while also overcoming their individual shortcomings. Since MRI is a purely diagnostic tool, the combination with a different entity is needed for therapeutic applications. In contrast, PDT by itself can only be utilized for the treatment of localized cancers and infections. By combining the two modalities, a situation of “1+1>2” can be achieved, since it allows PDT to be guided by MRI, and makes it easier to evaluate the results of PDT by imaging after each treatment cycle. Through this, it is possible to identify the relations between PS dose, light intensity, and tumor responsiveness all together allowing to modify the treatment scheme accordingly. Furthermore, theranostic compounds that combine MRI and PDT in a single molecule should allow the monitoring of drug distribution, pharmacokinetics and tumor treatment in real time 113. To achieve this goal, PSs and CAs can be linked covalently to generate novel multifunctional conjugate.

**Figure 21:**
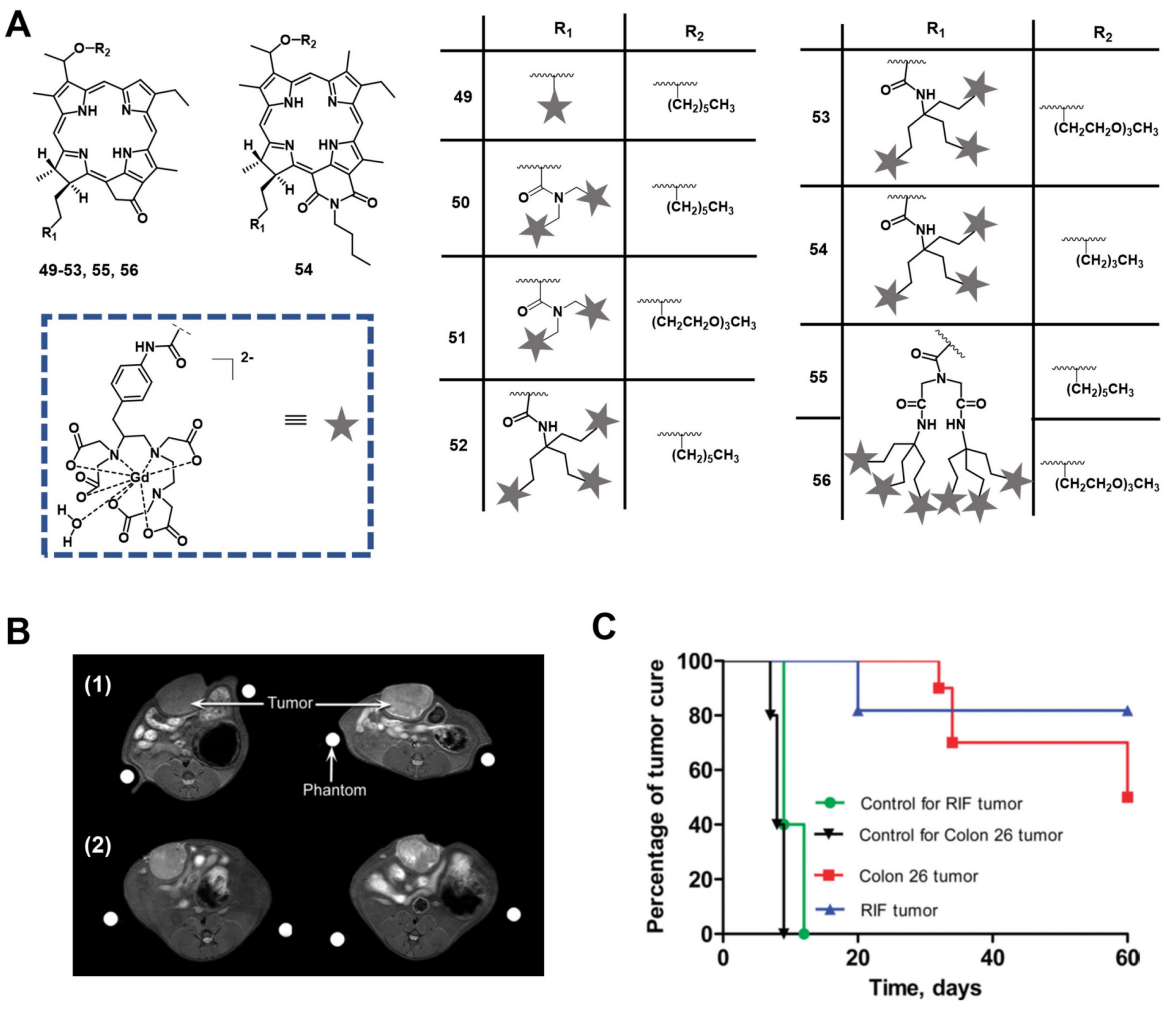
(A) Theranostic agents containing HPPH and Gadolinium(III) aminophenyl DTPA for a combined approach of MRI and PDT 114-116. (B) Increase of MR signal intensity was seen in rat Ward Colon tumors (arrow). Left: pre-injection; Right: 24 h post injection of complex **52** at 10 μmol/kg (1) and at 5 μmol/kg (2) 116. (C) *In vivo* PDT efficacy of complex **52** in (1) C3H mice bearing RIF tumors and (2) BALB/c mice bearing Colon 26 tumors at an imaging dose of 10 μmol/kg. Mice were irradiated with a laser light (70 J/cm^2^, 70 mW/cm^2^) and the tumor size was measured daily 116. Adapted and reproduced with permission from ref. 116. Copyright 2010 American Chemical Society.

In 2005, Pandey et al. developed a series of bifunctional theranostic agents, combining the PS (HPPH) and a well-known contrast agent (Gd(III) aminophenyl DTPA) to accomplish a theranostic scheme by using PDT and MRI 114-116. The biological activity of the conjugates was evaluated *in vivo* (C3H mice with subcutaneously implanted RIF-1 allografts). Compared with HPPH alone, the MR signal of the new compounds was greatly improved and the complex with two CA-motifs outperformed the complex with only a single CA motif in terms of in tumor imaging. However, both complexes (**49, 50**, Figure 21**)** exhibited significant skin phototoxicity. By replacing the alkyl sidechain of pyropheophytin with polyethylene glycol units, the resulting complex **51** retained a significant MR signal and showed anticancer PDT activity 8 h after intravenous injection, while its skin phototoxicity was greatly reduced.

To study further the effect of the number of CA-motifs in the theranostic agent on tumor MR imaging and PDT efficacy, 5 more complexes (**52-56**, Figure 21) were investigated. To determine the efficiency of PDT and MRI, *in vivo* experiments with compounds **52-56** in two mouse models were performed (C3H mice bearing RIF tumors and BALB/c mice bearing Colon 26 tumors). 24 h after the injection of the compounds, the mice were exposed to laser light (135 J/cm^2^, 75 mW/cm^2^) and afterwards observed daily for tumor regrowth or cure. All investigated compounds showed excellent MRI properties, and an increased number of Gd(III) aminophenyl DTPA units enhanced the tumor imaging (T1/T2 relaxation). However, both complexes, which carried six CA-motifs (**54, 56**), showed no, or only negligible PDT effects. In contrast to that, compounds with three CA-moieties (**52-54**) showed good properties for MRI and retained their PDT efficacy to varying degrees, but also induced high mouse mortality. However, if the light dose was reduced (665 nm, 70 J/cm^2^, 70 mW/cm^2^), but the injected dose (10 μmol/kg) remained the same, compound **52** was effective for the treatment of both tumor types, while exhibiting moderate skin toxicity (Figure 21).

In a more recent follow-up study, the group around Pandey conjugated HPPH with Gd(III)-DTPA and Gd(III)-DOTA in two different positions, and investigated the relaxivity and PDT efficacy of the compounds, as well as their suitability for fluorescence and MR imaging. However, due to their limited solubility, all four compounds were formulated into liposomes, and only compound **57**, which had Gd(III)-DOTA bound in position 17, was also used directly through dissolution in phosphate buffered saline (PBS). The results with liposomes showed that the Gd-DOTA conjugates had a better tumor accumulation, specificity and relaxivity, while conjugation in position 17 resulted in the optimal tumor accumulation being reached after a shorter period of time. This indicates that the position of conjugation and nature of chelator significantly influence the properties of the final compound, and thus its suitability for PDT and MRI. However, compound **57**, which was formulated in PBS, showed the overall most promising properties as a theranostic agent. It had in PBS a similar cytotoxicity upon light irradiation (up to 4 J/cm² with 3.2 mW/cm²) *in vitro* (Colon-26) to its liposomal formulation, while showing higher tumor accumulation, specificity and retention than the other compounds *in vivo* (BALB/c mice with Colon-26 allografts). Furthermore, it retained its PDT efficacy (20 J/cm² and 40 J/cm²) and showed an enhanced contrast comparable to previously investigated compounds with three Gd-DTPA moieties (52-54), indicating the significant difference in MR relaxivity and uptake different types of chelators can induce 117.

**Figure 23:**
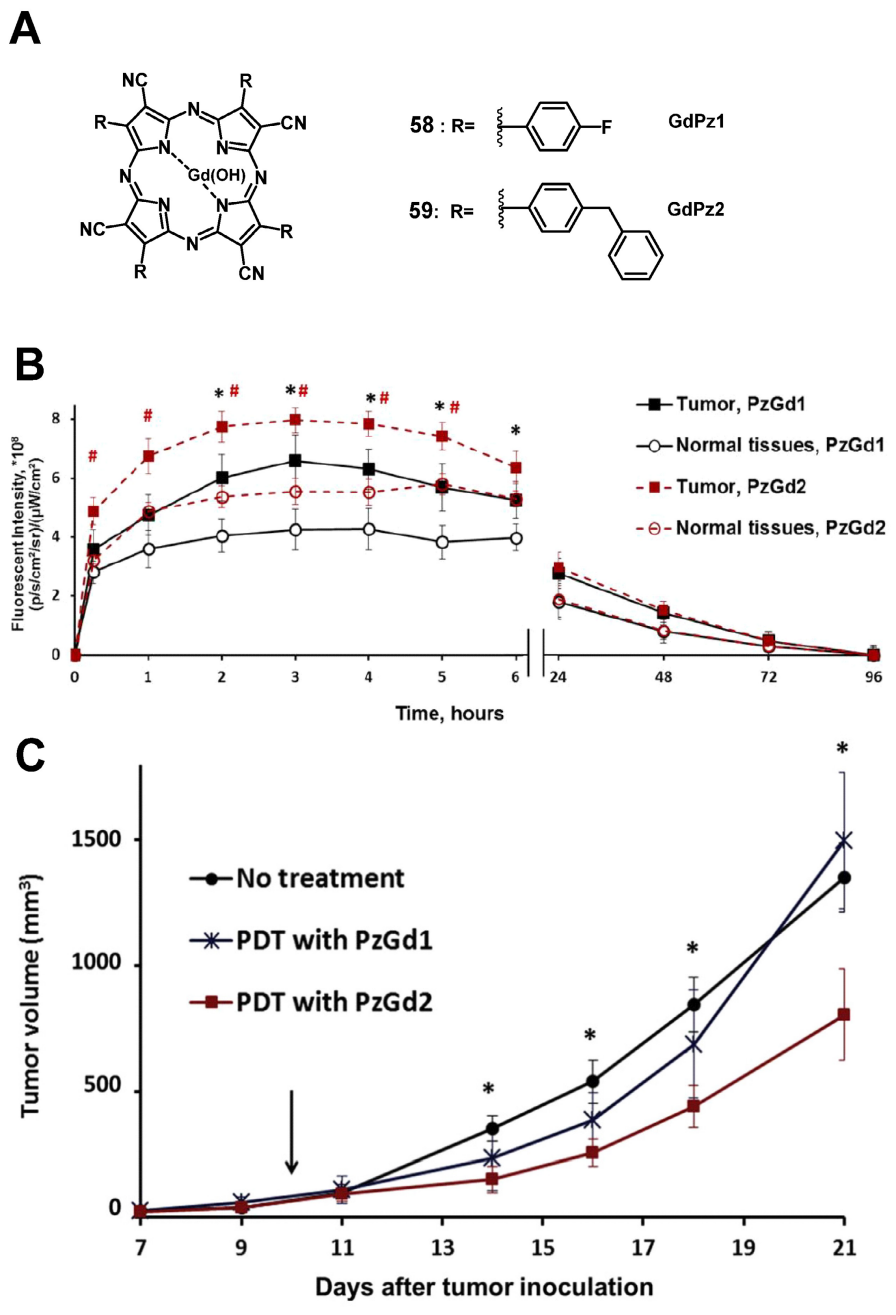
(A) Theranostic agents containing porphyrazine and a gadolinium (III) cation for a combined approach of MRI and PDT;^118^ (B) Quantification of the fluorescence signal in the tumor and normal tissues after i.v. administration of **58**-GdPz1 or **59**-GdPz2. Mean ± SD (n=7); (C) Effect of PDT with GdPz1 or GdPz2 on the growth of CT26 tumors in Balb/c mice. Mean ±SD (n=7) 118. Reproduced with permission from ref. 118. Copyright 2017 Elsevier B.V.

In 2017, Yuzhakova et al. designed two novel multifunctional agents (**58-59**, named GdPz1 and GdPz2, Figure 23 A) for *in vivo* MR/fluorescence imaging and PDT treatment of tumors 118. The results indicated that there is a good relaxivity value for compound **58** once dissolved in polymer polyimide brushes (4.67 mM^-1^s^-1^ at 9.6 T). *In vitro*, both compounds displayed a fast kinetic of cellular uptake and a pronounced photodynamic activity in the murine colon carcinoma (CT26 cells, the IC_50_ values of **58**-**59** are 3.5 μM and 1.7 μM, respectively upon irradiation at 615-635 nm, 10 J/cm^2^). **58-59** demonstrated the selective accumulation in tumor compared to the normal tissues by fluorescence imaging in BALB/c mice bearing CT26 tumors at a dose of 12 mg/kg (0.01 mmol/kg, Figure 23 B), while moderate tumor destruction was observed upon irradiation at 593 nm (30 min, 120 J/cm^2^) in the same animal model for PDT treatment (Figure 23 C).

Recently, Jin et al. reported the synthesis of a series of water-soluble Gd(III)-porphyrinoids (**60a-c**), which showed suitability as new phototheranostics based on PDT and MRI. The designed compounds contained *meso*-glycosylation and *β*-lactonization features, to improve their photophysical properties, solubility and biocompatibility 119. Hereby, compound **60c** showed the best NIR light-harvesting properties, as well as a similar ROS generation and higher photostability than the clinical photosensitizer methylene blue, and was thus chosen for an in-depth investigation. This indicated the importance of β-modifications for both the photophysical properties, as well as the relaxivity of such compounds. *In vitro* (HeLa), compound** 60c** showed promising relaxivity, as well as cytotoxicity upon light irradiation, and significant apoptosis induction could be observed, with the respective pathway being confirmed through the detection of Caspase 3 and PARP cleavage. *In vivo* (SCID mice, HeLa xenografts) the compound allowed real-time MRI upon intra-tumoral injection (50 mg/kg), with the highest intensity observed after 30 min, indicating a quick permeation and metabolism from the tumor site. Administration of compound **60c** alone (4 mg/kg) induced 30% tumor inhibition, while administration of 60c in combination with irradiation (660 nm, 0.3 W/cm² for 5 min) resulted in tumor growth suppression up to 90%.

Combining MRI with two-photon excited PDT is a promising trend for the development of therapeutic agents, but most investigations are still at the stage of *in vitro* evaluations and due to the lack of *in vivo* data, these examples are not covered in this review 120-122.

## 4. Nuclear Imaging and PDT

Nuclear imaging is a powerful non-invasive and highly sensitive imaging technique, which uses the decay emissions of radioactive isotopes to provide comprehensive images and information at the molecular or cellular level 123, 124. This imaging technology is characterized by the near unlimited penetration depth of ionizing radiation in biological tissues, which enables whole body scanning in contrast to optical imaging 125. Unlike MR and ultrasound imaging, which only deliver anatomical pictures, the high sensitivity of nuclear imaging allows for the measurement of extremely low concentrations of the respective radiotracer (pico- to nanomolar) and thus, enables the real-time imaging of tracer uptake without interfering with biological processes. Positron Emission Tomography (PET) and Single Photon Emission Computed Tomography (SPECT) are the two imaging modalities of nuclear medicine. The next sections will focus on theranostic compounds that effectively combine either of the two nuclear imaging modalities with PDT for *in vivo* applications. Hereby, we have arranged the described radionuclides according to their ascending atomic numbers.

### 4.1. Combination of PDT with PET

PET imaging is based on the use of radiopharmaceuticals incorporating a positron (β^+^)-emitting radionuclide 110, 126, 127. The positrons emitted by the radionuclides travel a short range through the surrounding tissue with a path length depending on their energy, which determines the spatial resolution of the PET images. When the β^+^ collides with an electron, the masses of both particles convert to energy. This annihilation results in the generation of two photons (511 keV) that are emitted in opposite directions, which in turn can be detected by coincidence measurement using circular detectors 125. Since PET is the most sensitive technique for quantitative *in vivo* measurements, it also allows for the detection of biological changes such as the overexpression of biomarkers, before the disease induces macroscopic lesions 128, 129. PET makes it possible to gather information about metabolic activity as well as biochemical processes for diagnosis and to monitor treatment response in real-time 128. Radionuclides used for PET imaging include metals as well as non-metals with a wide range of physical half-lives (t_½_) suited for different applications (Table 1). Radiometals come with a wide range of nuclear and chemical properties, as well as a convenient availability, but require the use of chelating agents to couple them to (bio)molecules, while non-metallic radionuclides often allow the direct radiolabeling of molecules without altering their structure significantly 130, 131.

Through the combination of PDT with a nuclear imaging modality like PET, it becomes possible not only to treat cancers through light irradiation but also track the PDT/PET conjugate in real time and monitor continuously tumors and their environment. At the same time, the targeted therapeutic effect of PDT complements the purely diagnostic properties of PET and *vice versa* 132. To date, several PET radionuclides have been combined with PSs to develop new theranostic compounds, however, this scientific field still remains in its early stage.

A prominent group of PSs are porphyrins and derivatives thereof due to their negligible dark toxicity, good metabolic stability and biocompatibility, as well as significant photoactivity. This makes them promising compounds for radiolabeling and resulted in their early investigation as nuclear imaging agents. Due to their tumor targeting properties, porphyrins and related structures kept being investigated over the years, but their potential as theranostic agents combining PDT and nuclear imaging has received considerable attention only during the last two decades 132, 135.

#### 4.1.1. PET/PDT with small organic radionuclides (^18^F and ^11^C)

**Figure 25:**
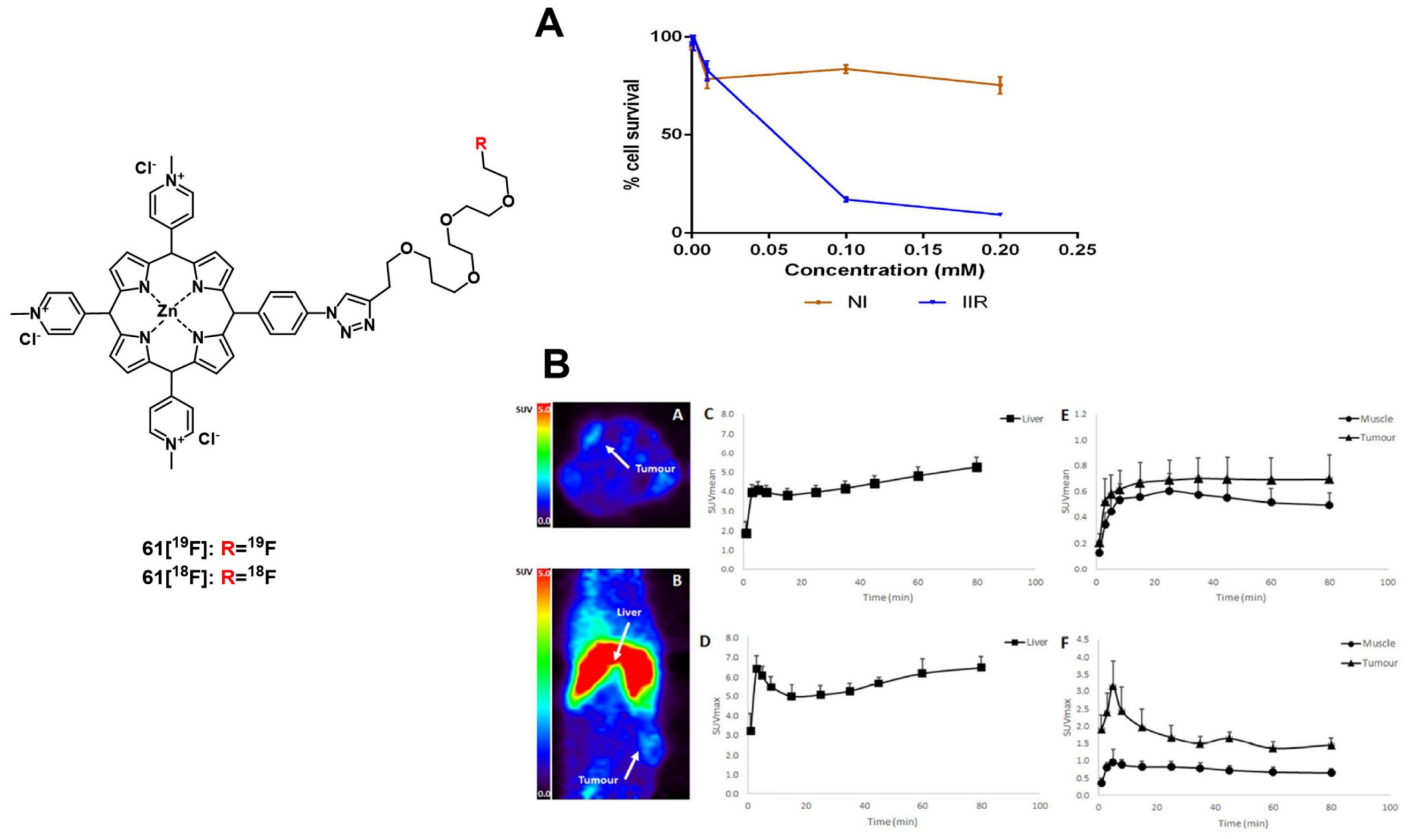
Chemical structures of the ^18^F-labelled porphyrin^2^ for the combination of PDT and PET. (A) Cytotoxicity experiment of [^19^F]F-**61** incubated with HT-29 cell lines following irradiation (IRR) and non-irradiated control (NI); (B) Dynamic image of complex [^18^F]F-**61** in tumor bearing mice 2. Reproduced with permission from ref. 2. Copyright 2015 American Chemical Society.

In an attempt to achieve the combination of PDT with PET, ^18^F was inserted into the structure of different PSs. In 2015, Entract et al. developed the first water-soluble porphyrin labeled with ^18^F by efficient click chemistry ([^18^F]F-**61**, Figure 25). *In vitro* (HT29 cells), the control compound ([^19^F]F-**61**) retained its phototoxicity with an LD_90_ of approx. 100 μM (irradiation light wavelength > 550 nm) and showed minimal dark toxicity. Further *in vivo* studies (CD-1 mice with HT29 and U87 xenografts) *via* PET/CT showed the accumulation of compound [^18^F]F-**61** (10 MBq, iv. *via* the tail vein) mainly in the liver but also in the tumors albeit to a smaller extent 2. Those results demonstrated the potential of theranostic agents that combine PET imaging and PDT treatment for the management of cancer.

Another commonly used radioisotope for PET-imaging is ^11^C. Due to the short physical half-life of ^11^C (t_½_ = 20.4 min), this radionuclide is less suited for theranostic approaches with PDT because PSs often need a significantly longer time to accumulate in tumors *in vivo*. To our knowledge, there is only one study reported in the literature that combines ^11^C with PDT, however, no *in vivo* data is available 136.

#### 4.1.2. PET/PDT with ^62^Zn

**Figure 26:**
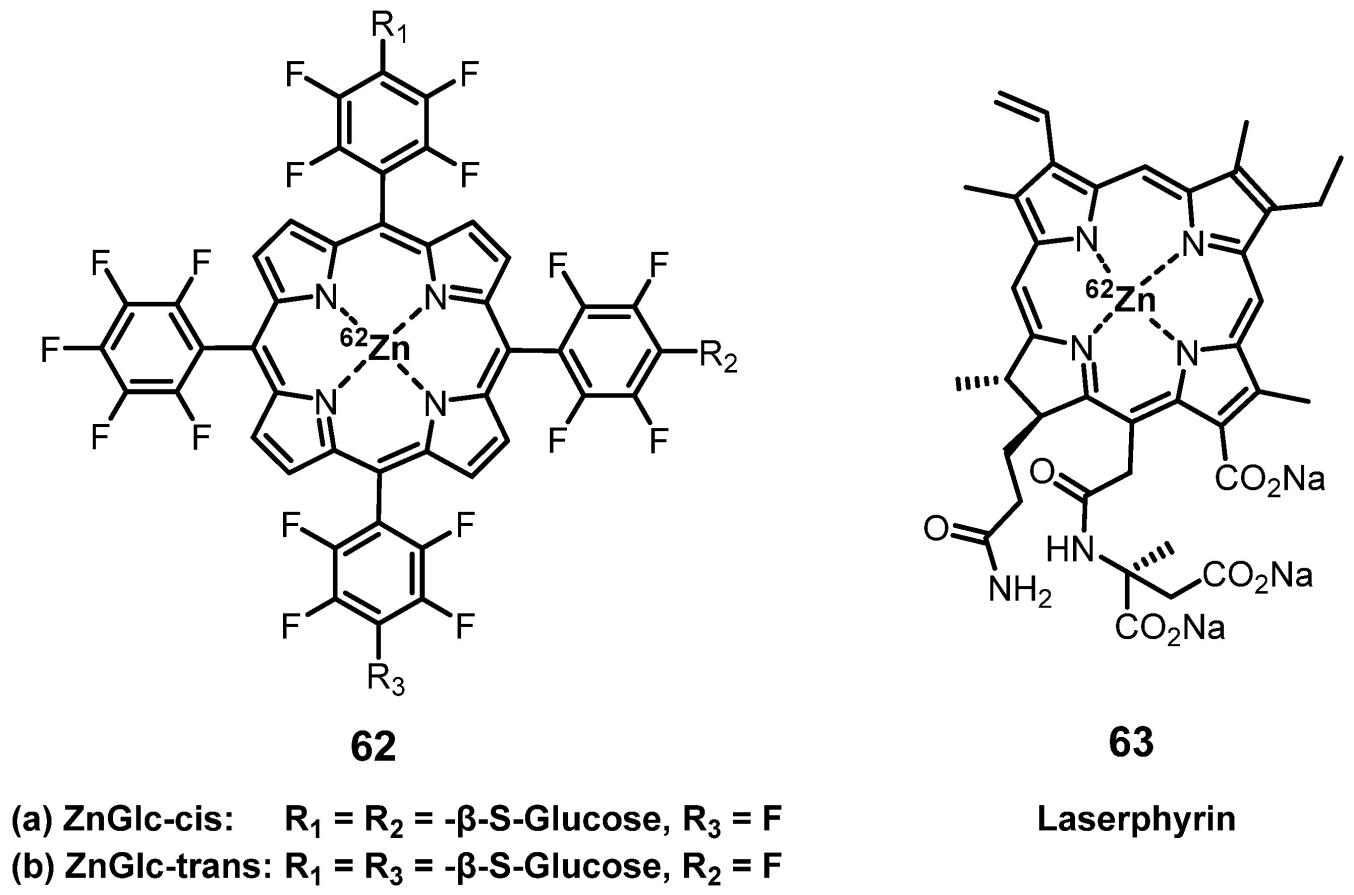
Structures of ^62^Zn-labeled porphyrin derivatives 137.

Another study with the less common PET radionuclide ^62^Zn was conducted by Tamura et al. in 2014. In this work, two isomeric glycosylated porphyrins ([^62^Zn]Zn**-62a/b**, Figure 26) as well as the approved PDT-agent Laserphyrin ([^62^Zn]Zn**-63**) were investigated in radiolabeled form. *In vitro*, the glycosylated compounds showed a higher PDT activity than Laserphyrin with the trans-isomer having the strongest toxic effect on RGM-1 cells upon photoirradiation (16 J/cm^2^, >500 nm). In animal experiments (tumor model mice implanted RGK cells), no fatal toxicity was observed and all compounds mainly accumulated in the liver as shown by PET imaging. It was also found that [^62^Zn]Zn-Laserphyrin accumulated in the brain to a greater extent than the ^62^Zn-labeled compounds [^62^Zn]Zn**-62a/b** whereas the latter showed a slightly increased uptake in the tumors. This indicates that ^62^Zn-labelled porphyrines might be suitable as PET/PDT theranostics, however, further optimization is necessary 137.

#### 4.1.3. PET/PDT with ^64^Cu

The PET radionuclide ^64^Cu has a significantly longer physical half-life (12.7 h) than ^11^C, which fits better for the purpose of combining it with molecules that exhibit slow pharamacokinetics 138. Different chelators have been investigated to develop ^64^Cu complexes that are stable* in vivo* 139, 140. However, developing a ^64^Cu-based PDT/PET theranostic agent is challenging due to the paramagnetic character of this radiometal, which upon integration into the porphyrin core can quench the therapeutic effect of the PS 141. On the other hand, it was already reported in the 1980s that the insertion of Cu into porphyrin-cores does not change the biodistribution and pharmacokinetics of the compounds 142. As a result, the option of administering a mixture of labeled and non-labeled porphyrins was investigated in recent years.

**Figure 27:**
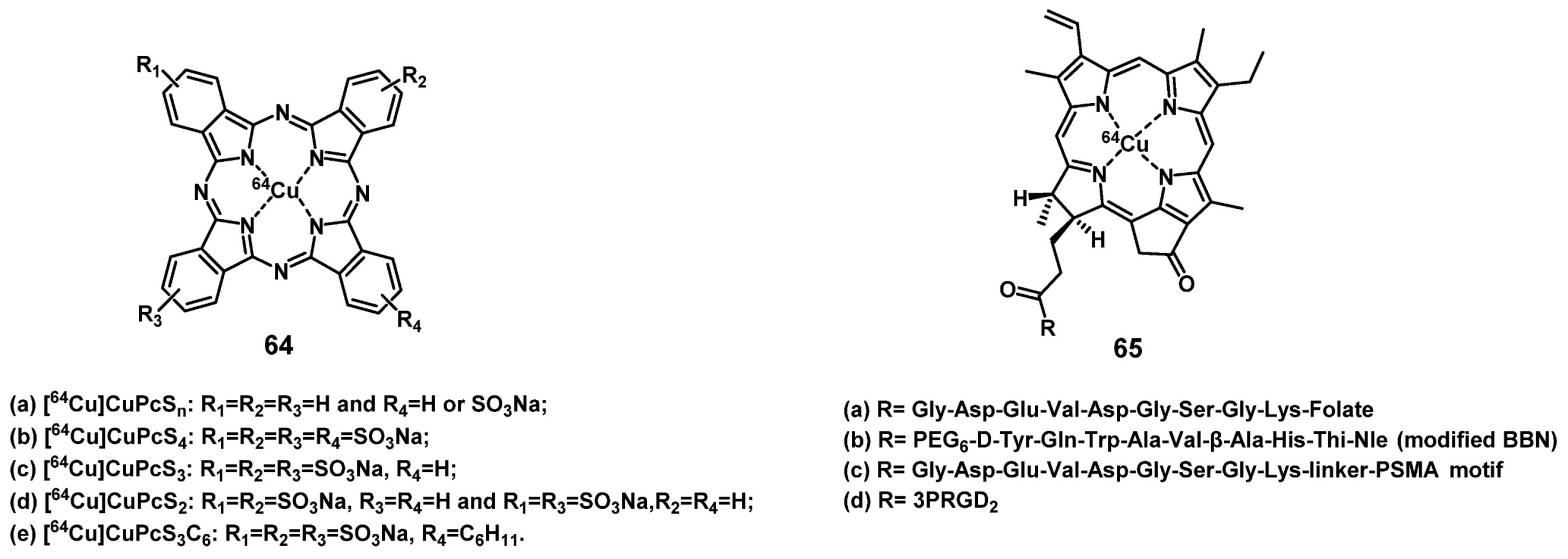
Chemical structures of tumor-targeting ^64^Cu-labeled sulfophthalocyanines 143, 144 and porphyrins 148-151 for combining PDT and PET.

In 2008, Soucy-Faulkner et al. described the preparation of a water soluble, ^64^Cu-labeled sulfophthalocyanine [^64^Cu]CuPcS ([^64^Cu]Cu**-64a**, Figure 27), a second generation PS, and evaluated its tumor uptake and biodistribution in a rat model (Fisher rats, MAC13762 tumors) using small animal PET 143. Most of the administered radioactivity (1-2 mCi iv.) was found accumulated in the liver and kidneys (12% ID/g and 20% ID/g, respectively), whereas tumor uptake remained very low (0.2% ID/g). Three years later, Ranyuk et al. prepared a series of similar compounds [^64^Cu]CuPcS_n_ ([^64^Cu]Cu**-64b-64e**, Figure 27) 144 and investigated their biodistribution in an EMT-6 tumor-bearing BALB/c mice by dynamic PET imaging. Of the differently sulfonated compounds investigated, only the amphiphilic derivatives [^64^Cu]CuPcS_2_ ([^64^Cu]Cu**-64d**, Figure 27) and [^64^Cu]CuPcS_3_C_6_ ([^64^Cu]Cu**-64e**, Figure 27) showed a slightly increased tumor uptake (1-1.5%ID/g). Nevertheless, this study demonstrated that PET imaging with [^64^Cu]CuPc can be used to study structure-PDT efficacy relationships for porphyrin-based PSs.

The combination of PDT with tumor-targeting (bio)molecules has been investigated by different research groups. Examples of targets of interest include the folate receptor-α (FRα), a membrane-bound protein with high affinity for binding folic acid and transporting it into cells 145 and the gastrin-releasing peptide receptor (GRPR) specific for bombesin (BBN) peptides that act as growth factors for many types of cancer 146. Also, the cyclic tripeptide Arg-Gly-Asp (RGD) is a high affinity ligand of integrin αvβ3 and has been widely used for the targeted delivery of RGD-conjugated molecular probes or nanoparticles to αvβ3-overexpressing tumors 147-152. In 2011, Shi et al. radiolabeled a previously investigated PS^153^ with ^64^Cu and studied its tumor-specificity and PDT activity *in vivo*. The designed ^64^Cu-labeled porphyrin-folate-conjugate connected *via* a peptidic linker ([^64^Cu]Cu**-65**, Figure 27) retained its tumor-selectivity and showed high uptake in folate-receptor positive tumors as well as a good tumor-to-background ratio *in vivo* (athymic nude mice, KB or MT-1 xenografts; ~500 µCi iv.) 148. Around the same time, Mukai et al. investigated independently the coupling of the PS PpIX to a BBN analogue, and labeled it with ^64^Cu for PET-imaging ([^64^Cu]Cu**-65b**, Figure 27). *In vivo* (BALB/c mice, PC3 xenografts) compound [^64^Cu]Cu**-63b** (5-20 MBq, iv.) showed rapid accumulation in the liver and kidneys, a prolonged blood circulation, but only limited tumor uptake (standardized uptake value of tumor < 0.2) 149.

A few years later, Harmatys et al. reported the synthesis of a radiolabeled pyropheophorbide-based PS coupled to the prostate-specific membrane antigen (PSMA) as a targeting vector ([^64^Cu]Cu**-65c**). *In vivo* (athymic nude mice, PC3-PIP or PC3-flu xenografts) the compound (~0.5 mCi, 25 nmol iv.) had a long plasma circulation time and showed increased tumor accumulation in the PSMA-positive tumor (9.74 ± 2.26 %ID/g), and upon irradiation (671 nm, 100 J/cm², 55 mW/cm², 30 or 50 nmol) induced tumor ablation, as well as tumor growth inhibition and in some cases no tumor-recurrence could be observed over 44 days post-PDT 150. Most recently, Fan et al. designed another pyropheophorbide-based, ^64^Cu-labelled probe containing an RGD motif (3P-RGD_2_), for multimodal imaging and PDT ([^64^Cu]Cu**-65d**) 151. The compound showed high specificity for α_v_β_3_ overexpressing tumors *in vivo* (mice with U87MG xenografts), which could be detected by both luminescence (λ_em_=740 nm) and PET-imaging (11.1 MBq iv.). Furthermore, rapid renal clearance, as well as a low accumulation in other organs and tissues was observed. These results indicate that the use of ^64^Cu-labeled porphyrins conjugated to tumor-targeting vectors has high potential for the design of new PET/PDT theranostic agents.

#### 4.1.4. PET/PDT with ^68^Ga

The PET radioisotope^ 68^Ga has attracted great interest in recent years due to its convenient availability by generators and a physical half-life of 68 min, which is suited the radiolabeling of tumor-targeting peptides or other biological vectors of fast pharmacokinetics through the use of appropriate chelators 138, 154, 155. For example, ^68^Ga-DOTATATE was approved by the FDA for PET imaging of neuroendocrine tumors in 2016, which highlights the potential of ^68^Ga for clinical applications 156.

**Figure 28:**
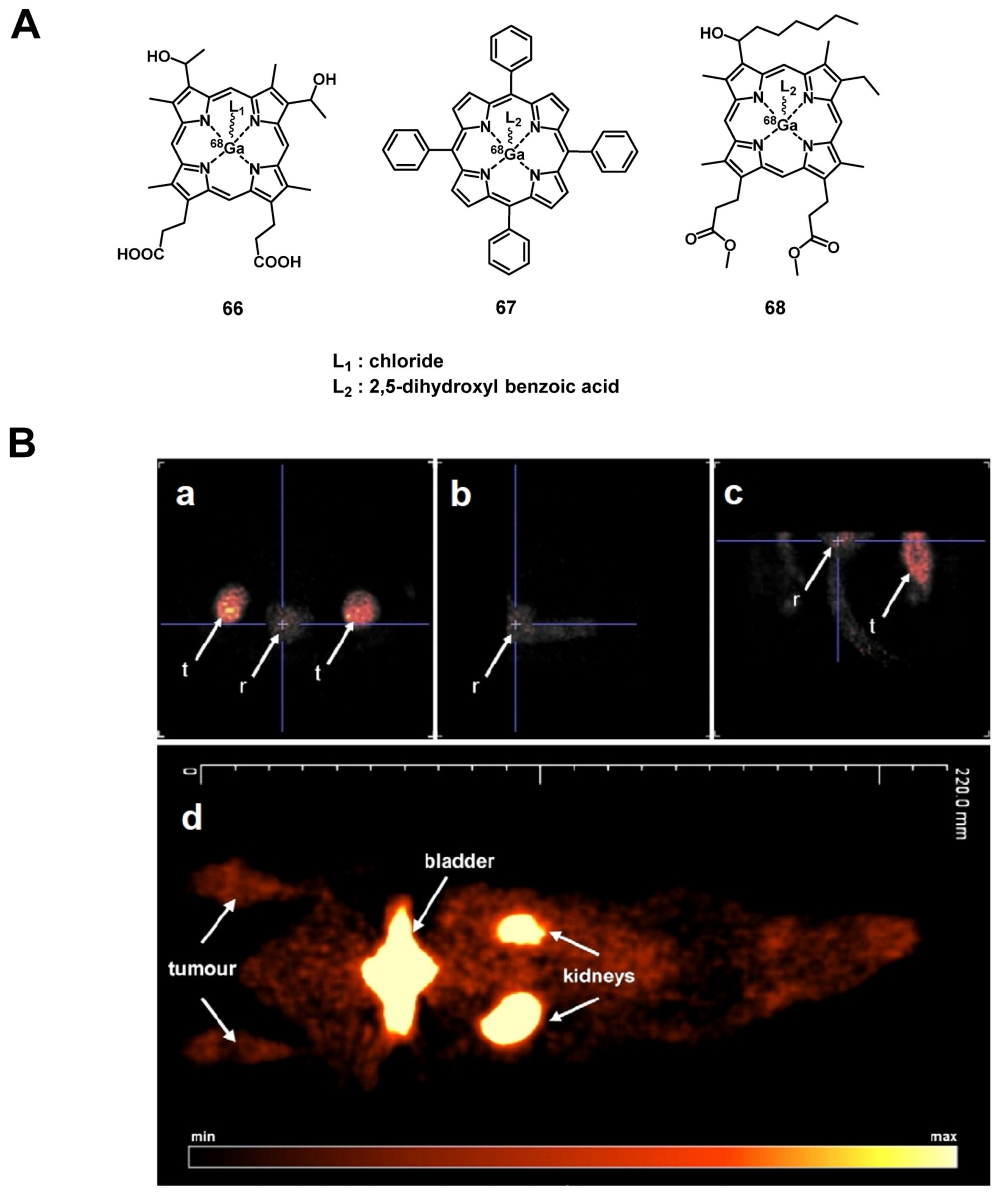
(A) Chemical structures of the ^68^Ga-labelled porphyrins derivatives [^68^Ga]Ga**-66-68**^158^; (B) PET/PET co-registration (10 min p.i) of the hind legs and testes after the injection of [^68^Ga]Ga**-66**. (a) Coronal, (b) sagittal and (c) transversal view. (t) denotes the tumor lesions, (r) denotes the reference region, (d) Rats were injected with 19 MBq [^68^Ga]Ga**-66**. Dynamic PET data were collected over 60 min. Representative static whole body PET image were obtained 60-80 min after tracer administration 158. Reproduced with permission from ref. 158. Copyright 2013 Elsevier B.V.

Another possibility to combine PET and PDT is represented by ^68^Ga-labelled porphyrins, since porphyrins are not only known good chelators for Ga but also one of the most promising ^1^O_2_ generators that can be applied *in vitro* and *in vivo*.^157^ In a first attempt to develop such theranostic agents, Zoller et al. development a series of ^68^Ga-labelled porphyrin derivatives ([^68^Ga]Ga-**66**-**68**, Figure 28) 158. *In vitro,* all compounds exhibited kinetic inertness and high thermodynamic stability over a period of 2 h in blood serum. Lipophilic compounds [^68^Ga]Ga**-67** and [^68^Ga]Ga**-68** showed up to 97% binding to human serum albumin (HSA) and >75% uptake by low-density lipoproteins (LDL) over a period of 120 min. For the more hydrophilic porphyrin [^68^Ga]Ga**-66** a HSA binding of only 26% and an insignificant binding to LDL was observed. *In vivo* PET imaging and biodistribution studies (Sparague Dawley rats bearing a DS sarcoma on each hind foot dorsum) revealed an increased uptake of [^68^Ga]Ga**-66** (19 MBq**,** 5-15 nmol, iv.) in both tumor lesions and tumor-bearing hind legs compared to non-tumor-inoculated forelegs (Figure 28 B). However, PDT data for the compounds have not been reported yet.

**Figure 29:**
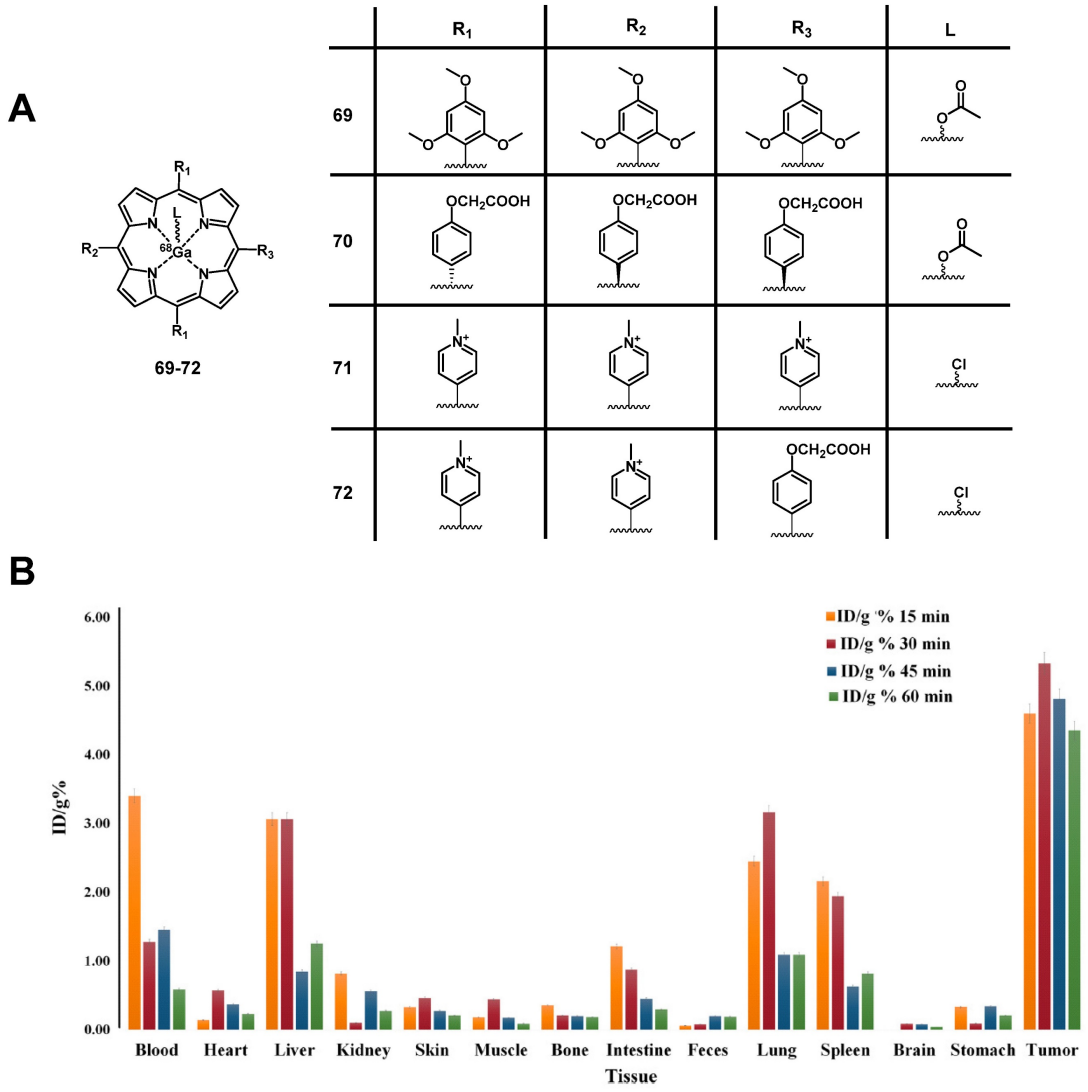
(A) Chemical structure of 68Ga-labelled porphyrin derivatives 159, 162, 163. (B) Biodistribution of [^68^Ga]Ga**-69** in mice bearing Fibrosarcoma tumors (n = 3) 159. Reproduced with permission from ref. 159. Copyright 2019 Springer Nature.

In 2018, Fazaeli et al. synthesized another water-soluble, ^68^Ga-labelled porphyrin [^68^Ga]Ga**-69**, which had significant PDT activity *in vitro* (IC_50_ value around 23 μM in HeLa and 18 μM in MCF-7 cells, yellow light, 1-4 J/cm^2^). In a subsequent *in vivo* biodistribution study (BALB/c mice bearing fibrosarcoma tumors), compound [^68^Ga]Ga**-69** showed a good and specific localization in the tumors (Figure 29), indicating its potential as a PET/PDT agent.^159^ Guleria et al. developed a different family of ^68^Ga-labelled porphyrin derivatives ([^68^Ga]Ga**-70-72**, Figure 29) 160-163. *In vitro,* the unlabeled form of porphyrin [^68^Ga]Ga**-70** showed a significant phototoxic effect on two different tumor cell lines (around 87 % HT1080 and 83 % A549 cell toxicity) upon light irradiation (405 and 418 nm, light doses of 0.02, 0.03 and 0.06 kJ/cm^2^). *In vivo* biodistribution studies (Swiss mice with fibrosarcoma tumors) revealed that the radiolabeled complex [^68^Ga]Ga**-70** (1.85 MBq, iv.) accumulated at the tumor site (2.49±0.16% ID/g at 1 h p.i) and was cleared from non-targeted organs over time 162. Recently, the same group reported two additional ^68^Ga-labelled porphyrin derivatives ([^68^Ga]Ga**-71**, [^68^Ga]Ga**-72**) 163. *In vitro* (A549 cells), only negligible dark toxicity was observed for both compounds, while the tetra-cationic porphyrin derivative [^68^Ga]Ga**-71** showed a significantly higher phototoxicity than its tri-cationic counterpart [^68^Ga]Ga**-72** (47.71 ± 0.71 and 43.98 ± 0.42 versus 77.67 ± 0.98 and 61.30 ± 4.59% cell proliferation at 0.01 and 0.04 kJ/cm^2^ light doses, respectively). *In vivo* (Swiss mice with fibrosarcoma tumors), both compounds showed significant tumor and liver uptake (3.47 ± 0.51% IA/g at 1 h p.i. for [^68^Ga]Ga**-71** and 9.41 ± 1.71% IA/g at 1 h p.i. for [^68^Ga]Ga**-72**) of the radiotracer (1.85 MBq, 50 µCi, iv.), whereas the tetra-cationic porphyrin derivative [^68^Ga]Ga**-71** exhibited a better retention in the tumor tissue (6.07 ± 0.03 %IA/g at 60 min p.i.) 163.

#### 4.1.5. PET/PDT with ^124^I

**Figure 30:**
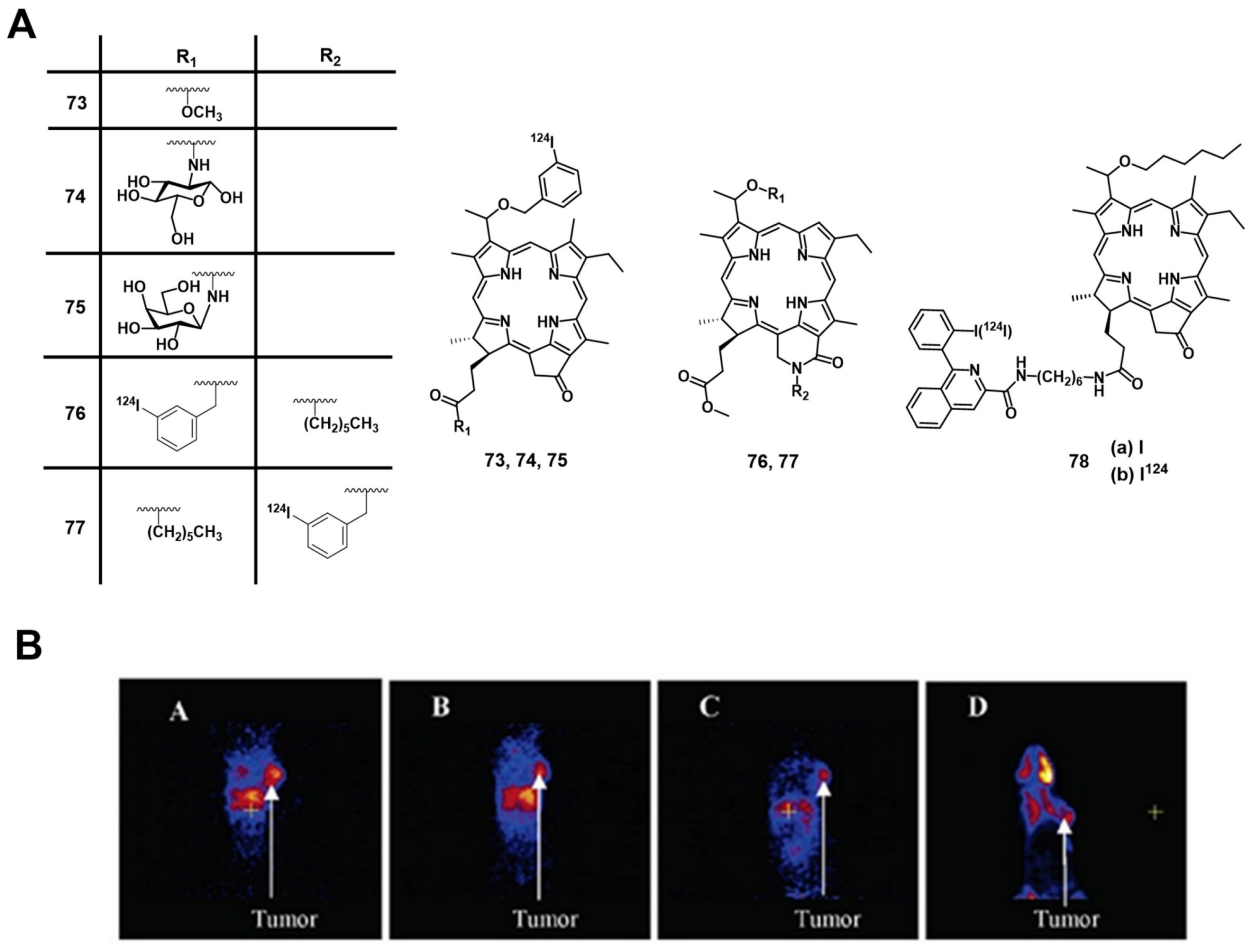
(A) Chemical structures of the ^124^I-labeled PSs **73** [166], **74-75** [167], **76-77** [168] and **78** [169] for the combination of PDT and PET. (B) Comparative PET images of mice bearing RIF tumors at 24 h (A), 48 h (B), and 72 h (C) post-injection of the pyropheophorbide lead-compound **73** and 90 min post-injection of [^18^F]FDG (D) 166. Reproduced with permission from ref. 166. Copyright 2005 American Chemical Society.

An important non-metallic radionuclide for PET imaging is ^124^I, which has one of the longest physical half-lives (t_½_ = 4.2 days) among all currently used PET nuclides. This allows to conduct complex, multi-step radiolabeling reactions and label large biomolecules like polypeptides or antibodies with slow pharmacokinetics 164, 165. For the purpose of combining PET with PDT, Pandey et al. developed a series of ^124^I-containing pyropheophorbide derivatives as potential multimodality agents based on on the structures of PSs used in clinical trials (Figure 30) 166-168. The first investigated radioiodinated compound **73** showed a strong PDT activity *in vitro* (RIF cells) comparable to the non-iodinated reference PS (HPPH, Figure 6). *In vivo* (C3H mice, RIF tumors) a significant curative effect could be observed upon irradiation (665 nm, 135 J/cm², 75 mW/cm², 24 h p.i.), which resulted not observable tumor-regrowth 60 days post-treatment in 80% of the mice (for 1.5 µmol/kg). When compared with the clinical PET agent [^18^F]FDG in imaging studies, complex **73** showed higher specific tumor accumulation and a good clearance from other tissue (Figure 30B) after intravenous injection of the tracer (0.56-1.85 MBq) 166. In follow-up experiments, the complex was modified with glucose and galactose moieties (compounds **74-75**, Figure 30), which increased the tumor uptake *in vivo* (C3H mice bearing RIF tumors and BALB/c mice with Colon-26 tumors). However, the addition of carbohydrates also resulted in a significantly higher uptake of the conjugate in the spleen and the liver resulting in a reduced contrast in the PET images compared to the corresponding non-glycosylated analogue **73**. Although the modified pyropheophorbides induced a curative effect *via* PDT (665 nm, 135 J/cm², 75 mW/cm²) in tumor bearing mice at lower doses than compound **73** (0.5 µmol/kg), they also showed a higher toxicity with increasing dose (20%, 60% and 100% death after drug administration (1.50 μmol/kg) for complexes **73**, **74**, and **75**, respectively).^167^ In an alternative approach, the compounds were structurally modified to generate purpurinimides (compounds **76-77**, Figure 30). *In vivo* experiments (C3H mice, RIF tumors) showed that the structure and position of the iodobenzyl substituents strongly influenced the biodistribution and tumor uptake of the conjugates. So far, none of the investigated multimodal compounds had better imaging capabilities or curative effects than the non-iodinated pyropheophorbide lead compound. Therefore further investigations in these directions are needed 168.

**Figure 31:**
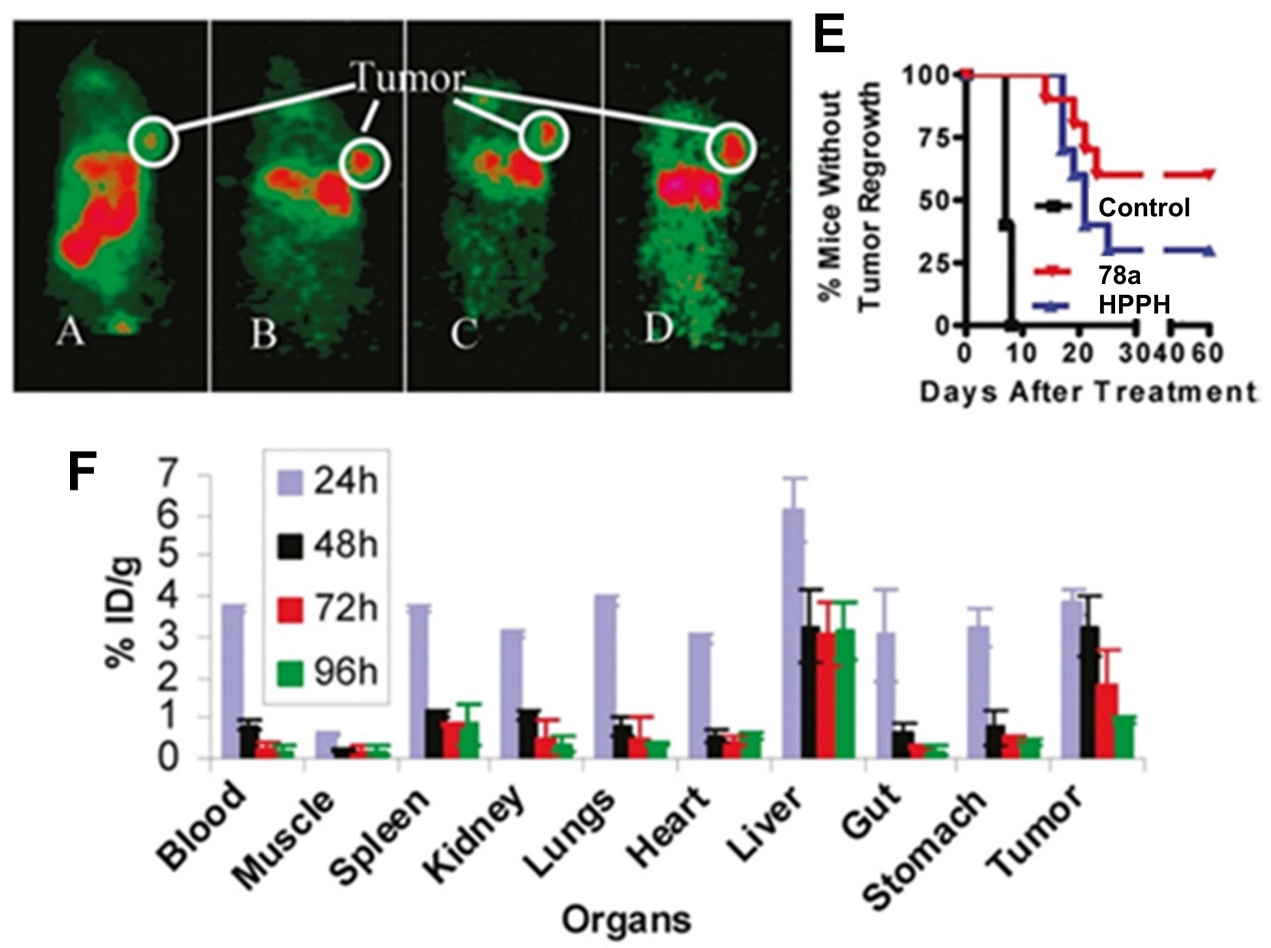
MicroPET imaging (coronal view) at 24 h (A), 48 h (B), 72 h (C), and 96 h (D); (E) Kaplan-Meier plot for the in vivo PDT efficacy of compounds HPPH and **78a**) at 0.4 μmol/kg dose. Light dose: 135 J/cm^2^, 75mW/cm^2^ (P<0.0001); (F) Biodistribution of compound **78a** for MDA-231 tumor bearing Scid mice at different time points p.i.^169^ Reproduced and adapted with permission from ref. 169. Copyright 2011 American Chemical Society.

To exploit the possibility to target the translocator protein (TSPO), which is overexpressed in a wide range of cancers, Chen et al. designed TSPO-specific PET/PDT theranostic agents by coupling a PS to the (radio)iodinated isoquinoline analogue PK 11195 (**78b**). During *in vivo* PDT experiments with Scid mice bearing MDA-231 tumors, the conjugate **78a-b** (Figure 30) showed an increased target specificity and PDT efficacy (135 J/cm², 60 % tumor cure rate at day 90) compared to HPPH, indicating its potential use for both imaging and therapy 169. As shown in Figure 31, the conjugate **78b** possessed strong imaging ability after p.i. (1.85 MBq), While biodistribution results also showed that the tumor has much higher **78a** uptake than any other organs except liver at 48, 72, and 96 h p.i. (Figure 31F).

**Figure 32:**
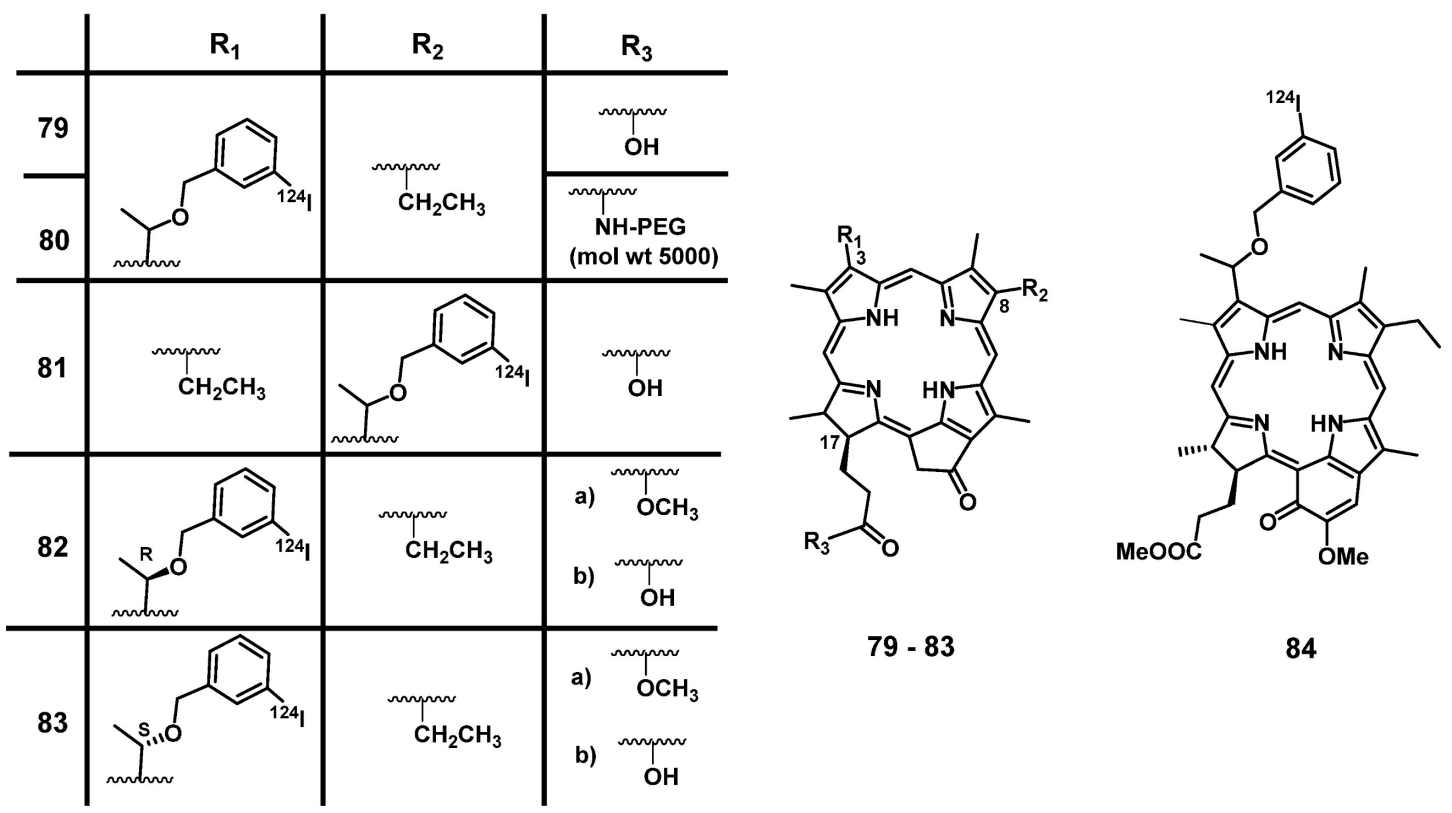
Chemical structures of the ^124^I-labeled PSs **79-81** [170],** 82-83** [171] and **84** [172] for the combination of PDT and PET.

In 2011, Srivatsan et al. synthesized a series of related pyropheophorbide analogues based on compound **73** and investigated the influence of the structural modifications on their biological properties both *in vitro* (Colon26 cells) and *in vivo* (BALB/c mice, Colon26 allografts) 170. Compounds with carboxylic acid groups in position 17^2^ (compound **79**, Figure 32) induced a significant increase in cellular uptake by diffusion and resulted in the deposition of the compound in the mitochondria, thereby increasing the PDT efficacy (660-674 nm, 128 J/cm², 14 mW/cm²). By linking a polyethylene-glycol (PEG) moiety to this position (compound **80**), the hydrophilicity could be increased further, which induced a cellular uptake by fluid phase pinocytosis and deposition in the endosomal/lysosomal compartment. It was also observed that changing the position of the *m*-iodo-benzyloxyethyl group (from position 3 to position 8) significantly reduced the phototherapeutic effect (compound **81**, Figure 32), while isomers with a different position of the reduced ring in the pyropheophorbide backbone were found equally effective.

Prior to entering Phase I human clinical trials, Srivatsan et al. further evaluated the activity of single enantiomers of the parent structure (compounds **82-83**, Figure 32) in 2015. The group investigated the impact of the chirality at position-3^1^ *in vitro* (Colon26 cells) and *in vivo* (BALB/c mice with Colon26 allografts and SCID mice with NSCLC xenografts). Parameters studied included cellular uptake, intracellular localization, epithelial tumor cell specific retention *in vitro,* and luminescence and PET imaging (1.84 MBq iv.) as well as photosensitization capabilities *in vivo* 171. The experiments revealed that the corresponding pure isomers had properties similar to the racemic mixture. However, the methyl ester analogues were preferentially retained in lung tumor epithelial cells. On the other hand, the carboxylic acid analogs showed a higher uptake in fibroblasts and epithelial cells, as well as a higher clearance rate since derivatives with carboxylic acids at this position are substrates for the translocation protein ABCG2.

Four years later, the group of Pandey modified the pyropheophorbide structure by including a fused methoxy-cyclohexenone in its cyclic backbone for a shift of absorbance to longer wave length (compound **84**, Figure 32) 172. *In vivo* (BALB/c mice, Colon-26 allografts)*,* the compound showed promising tumor uptake (9.8% ID/g at 24 h p.i. of 25 µCi iv.) as well as good tumor contrast for PET imaging (best contrast 72 h p.i.). Furthermore, it was observed that compound **84** exhibited lower luminescence and singlet oxygen yields than compound **73**, resulting in a limited PDT efficacy (665 or 789 nm, 135 J/cm², 75 mW/cm²). Therefore, compound **84** provides access to a novel PET imaging agent with limited PDT activity, and no skin phototoxicity. This compound could be used to study, for example, brain and kidney cancer as well as pancreatic carcinomas for which the application of [^18^F]FDG is restricted.

In 2020, the group further reported on the properties of a ^124^I-labeled pyropheophorbide (**82a/83a**, Figure 32), which by then had been named “PET-ONCO”. In various *in vivo* models (BALB/c and SCID mice, bearing Colon26, Lewis lung, 4T1, GL261, U87, UMUC3 or Panc-1 tumors), compound **82a/83a** showed high uptake in the different tumor tissues (e.g. primary tumor, lung metastases, brain tumor) after iv. injection (50 µCi). The highest tumor uptake was observed 24 h p.i. but improved PET-contrast was also achieved after 48 h and 72 h. Furthermore, in a toxicity study with BALB/c mice and Sprague-Dawley rats, no toxic effects could be observed. This demonstrated the potential of “PET-ONCO” for imaging-guided PDT 173. Based on these results, the group investigated the PDT efficacy and toxicity of the non-radioactive (^127^I) PET-ONCO *in vitro* and *in vivo*. No dark toxicity could be observed *in vitro* (A549/H460/MDA-MB-43 cell lines) even when higher than therapeutic doses (1 µmol/kg) were administered and upon light irradiation (665 nm, 135 J/cm²), a strong cytotoxic effect could be induced. In animal studies (SCID mice bearing PDX tumors (SCLC and NSCLC)), the PS **82a/83a** also showed no significant toxicity in the dark, while high tumor specificity and PDT efficiency (665 nm, 128-135 J/cm², 14 and 75 mW/cm²), were observed in two different formulations (Tween®-80 and Pluronic® F-127). Co-administration of PET-ONCO with doxorubicin also improved the long-term curative effect (60 days). Thus, PET-ONCO represents a promising candidate for new approaches in the diagnosis and therapy of different cancer 174.

### 4.2. Combination of PDT with SPECT

Like PET, SPECT is a nuclear imaging modality that uses metallic or non-metallic radionuclides of variable physical half-life (t_½_). SPECT is widely used in nuclear medicine for the same purpose as PET however, it is more affordable but has a lower spatial resolution. Compared to PET, the radionuclides for SPECT directly emit γ-photons during their decay as the result of stabilization processes in the atomic nucleus. The emitted photons can then be detected by highly sensitive SPECT cameras 175, 176.

The most common radionuclides used for SPECT are shown in Table 2. Among them, ^99m^Tc is the most prominent one since it is readily available *via* generator technology and allows for facile preparation of radiolabeled molecules through the use of convenient labelling kits. Importantly, ^99m^Tc has a physical half-life and γ-energy well suited for medical SPECT imaging 177. ^99m^Tc has a rich coordination chemistry and complexes of the radiometal in its oxidation state -3 to +7 with a large variety of structurally diverse ligand systems have been reported 178, some of which (e.g, ^99m^Tc^V^) have found routine application in the clinic.

#### 4.2.1. SPECT/PDT with ^99m^Tc

**Figure 33:**
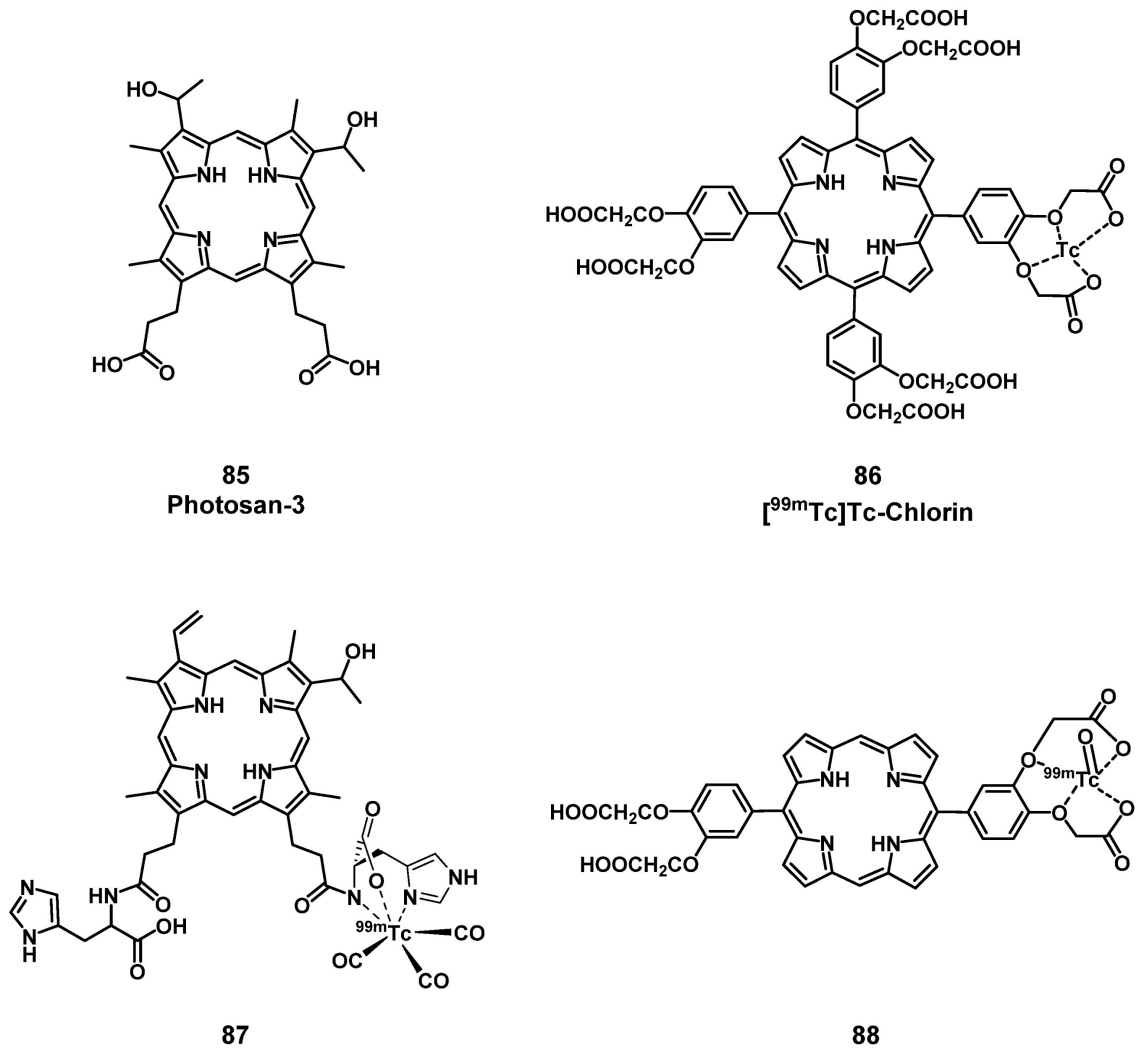
Chemical structures of the photosensitizer Photosan-3, and porphyrin derivatives which were radiolabeled with ^99m^Tc(I)-tricarbonyl (for **87**) and ^99m^Tc(V)-oxo cores (for **86**, **88**) for the combination of PDT and SPECT 179-184.

In 2000, Babbar et al. developed theranostic agents combining PDT and SPECT through the radiolabeling of the clinically used PS **85** (Photosan-3, Figure 33) with ^99m^Tc 179. The radiolabeled compound [^99m^Tc]Tc-Photoscan-**85** was shown to be stable *in vitro* and *in vivo* and was preferably taken up by neoplastic tissue (BALB/c mice, EAT tumors, 4 MBq, iv.) to an extent comparable to that of the unlabeled PS 179. About the same time, Murugesan et al. independently synthesized chlorin **86**, labelled it with ^99m^Tc and investigated the resulting compound [^99m^Tc]Tc-Chlorin **86** *in vivo* using Wistar-rats with N-nitroso-methylurea (NMU)-induced mammary tumors. Both ^99m^Tc-labeled compounds (porphyrin and chlorin) showed significant tumor accumulation determined by SPECT imaging, as well as fast renal clearance demonstrating their potential as tumor imaging agents. The chlorin derivative was additionally investigated as a PS for PDT (40 mg/kg p.i., 650 nm, varying light doses, 10-100 mW for 1-15 min) in fibrosarcoma-bearing Swiss mice. After accumulation of the compound in the tumors and light irradiation, up to 80% reduction of the tumor volume was achieved 180-182.

In 2012, Liu et al. coupled histidine to an inseparable mixture of three different porphyrin derivatives (e.g., compound **87**, Figure 33), which were identified as main components of an investigational PS in clinical trials hematoporphyrin and labelled them with [^99m^Tc(CO)_3_]^+^. After injection of ^99m^Tc-**87** in mice bearing S180 tumor (50 μg/ mL, 0.7 MBq), it was found that the compounds had a similar biodistribution as the unlabeled PS, resulting in a significant accumulation in the tumors (tumor-to-muscle (T/M) ratio was around 5.27±0.12 at 24 h p.i.). The results of this study indicated that compound ^99m^Tc-**87** might be suitable for the *in vivo* dose-finding studies during PDT 183.

Santos et al. reported the synthesis of meso-bis[3,4-bis-(carboxymethyleneoxy)phyenyl]-porphyrin **88** and its radiolabeling with the ^99m^Tc(V)-oxo core. Compound ^99m^Tc-**88** showed a high stability *in vitro* as well as *in vivo*. However, a fast clearance *via* liver and kidneys in a mouse model (BALB/c mice, WiDr and H1299 xenografts) caused a low uptake in cancer cells (the maximum uptake of WiDr and H1299 cells was 2.44 ± 0.60 % and 2.63 ± 0.99 %, respectively), which indicates a reduced PDT activity. Nevertheless, the compound showed potential as a tumor imaging agent due to a sufficient tumor-to-muscle ratio (3.33 ± 1.22 and 3.55 ± 1.29 for WiDr-bearing mice and H1299-bearing mice at 360 min p.i., respectively) 184.

We note that Yap et al. and the Alessio group developed several promising porphyrin derivatives with a chelating system for the radiolabeling with ^99m^Tc and investigated the compounds as novel theranostic PDT/SPECT agents. However, these examples will not be discussed here in detail due to the lack of *in vivo* data 185-189.

#### 4.2.2. SPECT/PDT with ^111^In

**Figure 34:**
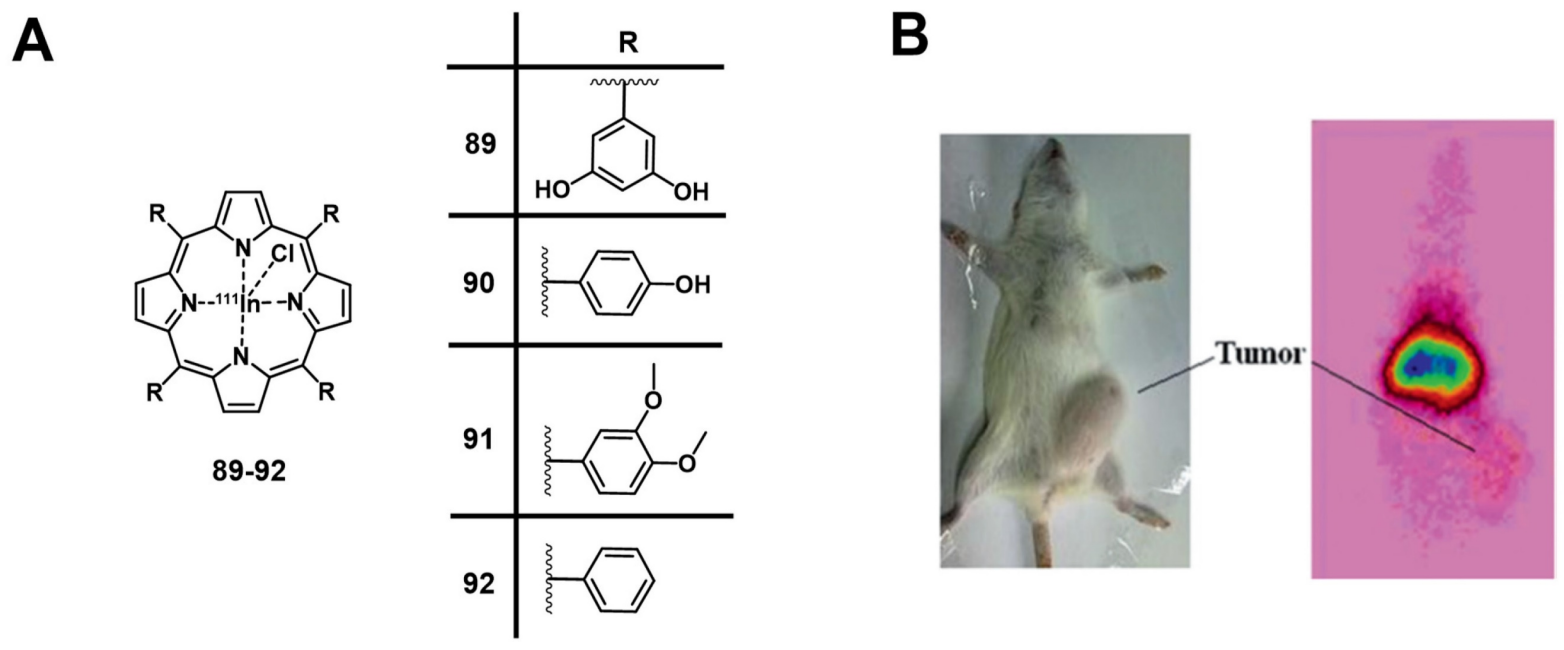
(A) Chemical structure of an [^111^In]In-porphyrins for PDT and SPECT 191, 192; (B) Planar SPECT image of a rat bearing a breast tumor (induced by injection of polyaromatic hydrocarbon) 24 h after i.v. injection of radiotracer **92** (1.85MBq) via the tail vein 192. Reproduced with permission from ref. 192, Copyright 2016 Radiochimica acta.

Another important commercial radionuclide is the cyclotron-produced ^111^In (Table 2). The longer physical half-life of ^111^In (t_½_ = 2.8 d) makes it especially suitable for the SPECT imaging of biological molecules with longer biological half-life (e.g., antibodies) that results in a slower accumulation at the target as well as a reduced clearance rate 190. In 2014, Sadeghi et al. developed three different ^111^In-labelled porphyrins ([^111^In]In-TDHPP **89**, [^111^In]In-THPP **90** and [^111^In]In-TDMPP **91,** Figure 34) as tumor seeking PSs and SPECT probes. The compounds were stable in human serum *in vitro* and biodistribution data from experiments with wild-type rats indicated their accumulation in the liver and the kidneys. The higher kidney uptake of [^111^In]In-TDMPP **90** compared to the other two compounds indicates fast renal excretion, a desired general characteristic for imaging probes. This makes compound [^111^In]In-**90** a promising candidate for SPECT imaging during PDT 191.

Similarly, Fazaeli et al. also developed an ^111^In-labelled tetra-phenyl porphyrin complex **92** (Figure 34) as a potential PDT/SEPCT theranostic agent. Results from biodistribution experiments in Sprague Dawley® rats showed that [^111^In]In-**92** (7-7.4 MBq) was metabolically stable for at least 24 h p.i. and excreted mainly *via* the gastrointestinal tract (likely metabolites). Biodistribution studies confirmed a high skin uptake of [^111^In]In-**92** (3.8 % ID/g at 24 p.i.), SPECT image showed a distinct tumor accumulation 192.

Most PCas overexpresses prostate-specific membrane antigen (PSMA), an essential membrane glycoprotein. PSMA has been recognized as an ideal target for PCa imaging and therapy as it is specifically overexpressed in 90-100% of localized PCa lesions, malignant lymph nodes, and bone metastases 193-195. Lütje et al. coupled the anti-PSMA antibody D2B with the commercial PS IRDye700DX and labelled the immune-conjugate with ^111^In. The immunoconjugate showed significant accumulation in PSMA-positive tumors *in vivo* (40.3 ± 2.6% of the added activity of ^111^In-DTPA-D2B-IRDye700DX after 48 h p.i. in BALB/c mice with xenografts of LS174T-PSMA or LS174T-wildtype cells), which could be visualized with both SPECT (5-8 MBq iv.) and NIR luminescence imaging. Furthermore, inhibition of tumor growth could be achieved upon light irradiation (670-710 nm; 50-150 J/cm^2^) and the survival rate of the treated mice improved considerably (median survival after treatment = 73 days / median survival without treatment = 16 days). It should be noted, that the study also revealed a high uptake of the labeled antibody in the liver and spleen. Due to the selective light irradiation of tumor regions, no or only reversible toxic effects could be observed in non-targeted organs and tissues 196.

**Figure 35:**
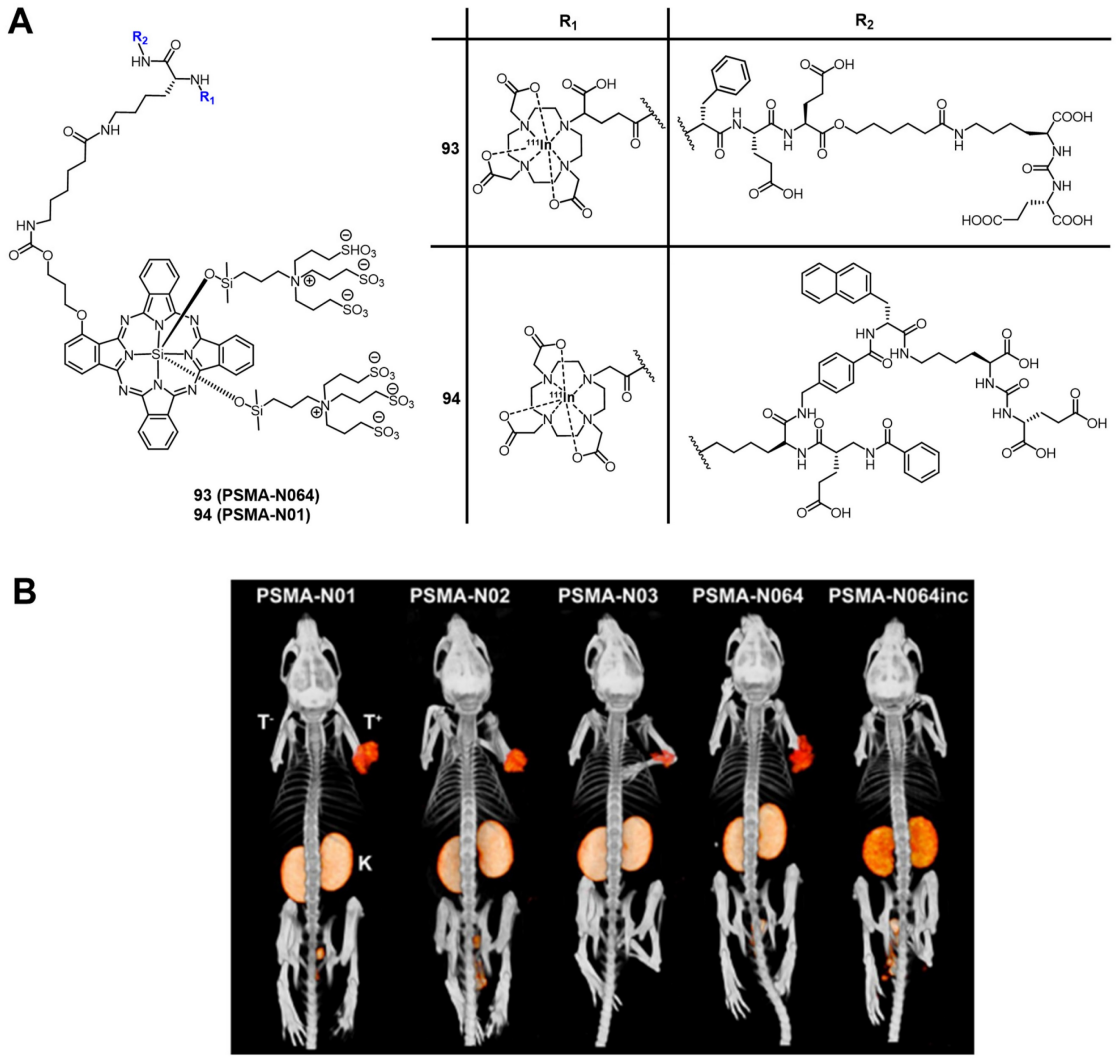
(A) Example structure of PSMA-targeting, multimodality agents for SPECT/PDT, which can be labelled with ^111^In 197. (B) SPECT/CT imaging of dual-labeled PSMA-ligands in mice with LS174T-PSMA (right shoulder) and wildtype LS174T (left shoulder) tumors 198. Reproduced with permission from ref. 198. Copyright 2022 Springer Nature.

Derks et al. recently developed a range of structurally different PSMA ligands and coupled them to [^111^In]In-DOTA and IRDye700DX (e.g., compound **93**, Figure 35) 197, 198. In a first study, a series of PSMA ligands incorporating various linkers between the different moieties were characterized *in vitro* (LS174T-PSMA and LS174T-wildtype cells). The *in vitro* results showed that additional negative charges in the linker improved the specificity of the conjugates towards PSMA and the cellular binding and internalization. Upon NIR light irradiation, PDT (670-710 nm, 100 J/cm²) could be induced resulting in 26-34% cell viability. The three best ligands were further investigated *in vivo* (BALB/c mice with LS174T-PSMA and LS174T tumors). Hereby, PSMA-specific tumor uptake of the compounds could be observed *via* luminescence and SPECT imaging (10 MBq iv.), however, a high unspecific uptake in several organs like spleen and kidneys was also observed 197.

In a follow-up study, the same group designed three more ^111^In-labeled, photoactive PSMA ligands with modified linkers (e.g., [^111^In]In-N01 **94**, Figure 35). *In vitro* (LS174T-PSMA cells), the compounds showed no cytotoxicity in the dark (> 80 % cell viability), but upon light irradiation (690 nm, 100 J/cm²) a strong cytotoxic effect could be induced (20-25 % viability). In BALB/c mice bearing two different tumors (LS174T-PSMA and LS174T-wildtype as control) the compounds (10 MBq, 0.3 nmol, iv.) showed a significantly improved tumor targeting for PSMA-positive tumors (20-23% uptake) in comparison to the previously studied PSMA-ligand **93** (14% uptake), while the uptake in PSMA-negative tumors remained negligible (0.6-1,1 %) 198. No *in vivo* data on the use of the compounds for PDT has yet been reported.

In another recent study, Renard et al. conjugated the chelator DTPA for complexation of ^111^In and IRDye700DX to the variable domain heavy chain only antibody (VHH) 7D12, using a dichlorotetrazine platform to achieve a site-specific conjugation. The modified VHH was stable under physiological conditions (PBS, blood serum) and induced cell death *in vitro* (A431 cells) upon irradiation (690 nm, 30-90 J/cm², 50-200 mW/cm²). *In vivo* (mice bearing A431 xenografts), an accumulation of the radioconjugate in tumors, liver, and kidneys, and a rapid clearance could be observed *via* SPECT (10 MBq, 3 µg, iv.) and luminescence imaging. Furthermore, it could be shown that the VHH retained its specificity towards its target the anti-epidermal growth factor receptor (EGFR) by blocking experiments with unlabeled cetuximab, which reduced the tumor uptake by nearly half. This indicates that the compound might be suitable for targeted ablation of tumors *via* PDT, and shows that the combination of EGFR targeting antibodies, which are currently investigated in clinical trials, with radiotracers and PSs is a viable strategy 199.

#### 4.2.3. SPECT/PDT with ^131^I

**Figure 36:**
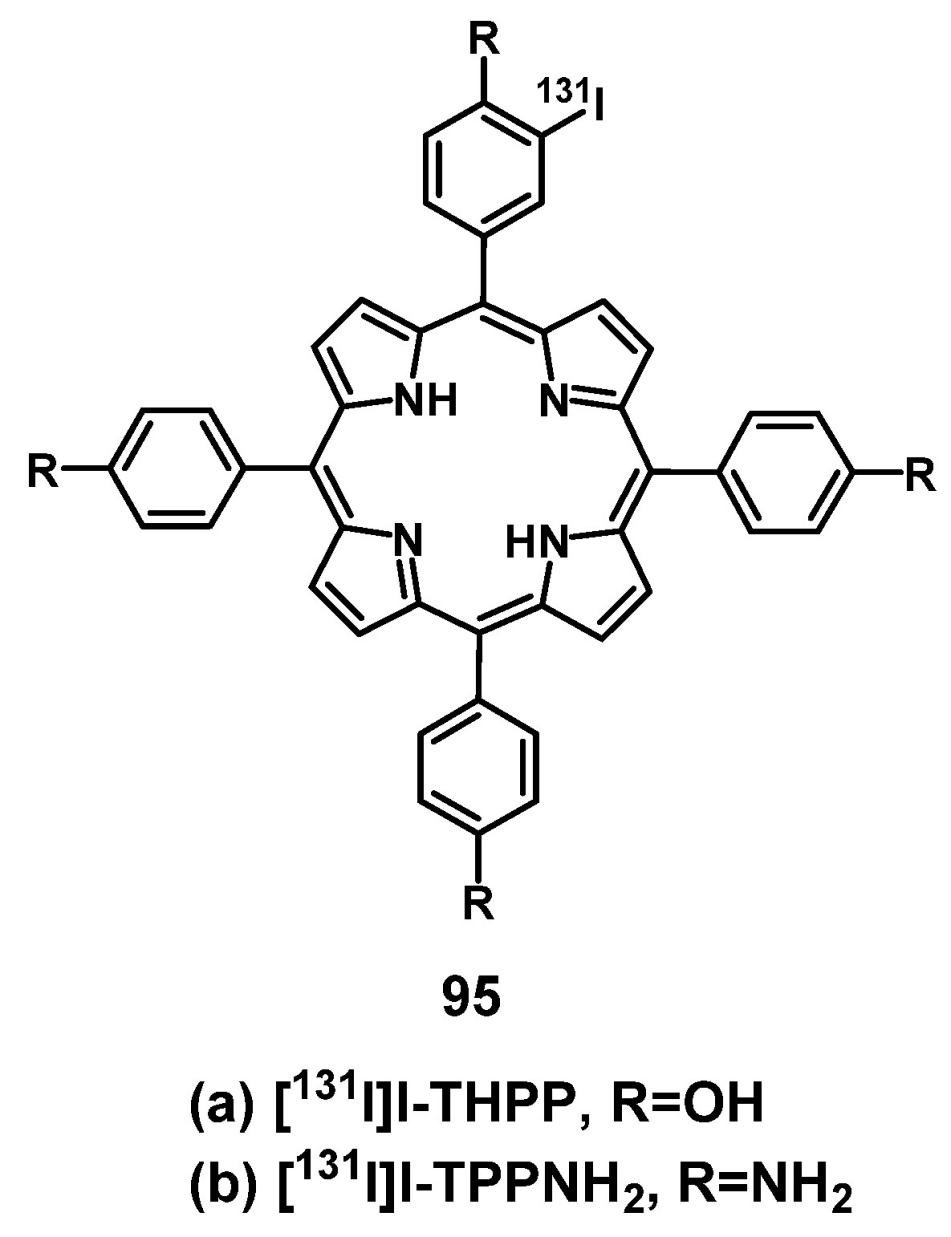
Chemical structures of the ^131^I-labeled THPP and TPPNH_2_ for the combination of PDT with endoradiotherapy and SPECT 200.

Besides pure γ-emitters, it is also possible to combine therapeutic, particle-emitting radionuclides that also have a concomitant γ-radiation with PSs in order to create agents for PDT, endoradiotherapy, and SPECT imaging. Song et al. reported the synthesis of ^131^I-labeled THPP **95a** as well as 5,10,15,20-tetrakis(4-aminophenyl)porphyrin (TPPNH_2_, **95b**) for a combination treatment using PDT and endoradiotherapy (Figure 36) 200.* In vitro* (SMMC-7721 and LO2 cells), radiolabeled THPP and TPPNH_2_ showed good stability and low lipophilicity. *In vivo* (BALB/c mice with SMMC-7722 xenografts), both compounds (37 MBq per mouse, injection into tumors in situ) exhibited an increased uptake in the tumors as observed by SPECT imaging. The volume of tumors treated with compounds **95a** and **95b** in combination with light irradiation (400-600 nm, 50 mW/cm², 15 min, at 5 min and 24 h p.i.), were reduced by 64±11% and 54±10% respectively, 14 days post-treatment. The combination of PDT with endoradiotherapy is an appealing approach, however, whether or not it results in an improved treatment *via* additive or synergistic effects of the individual therapies remains to be shown.

## 5. Conclusions

PDT is a rapidly advancing field in modern medicine. PSs can function as both sensitizing agents and as primary therapeutics upon light irradiation for a variety of diseases - ranging from cancer and inflammations to bacterial infections. However, the limited penetration depth of visible light in biological tissue may restrict PDT applications to easily reachable and accessible targets while also making the visualization of the agents by imaging more challenging. As a result, various methods to circumvent these limitations are currently being investigated.

In this review, we highlighted different designs of molecular theranostic agents containing both photosensitizers and different imaging probes in an effort to overcome current limitations of PDT imaging modalities. By shifting the inherent absorbance of PSs into the NIR-range through modifications of their structure or linking them to other NIR-luminescent molecules, the wave length and therefore tissue penetration range of the emitted light can be improved significantly. This in turn facilitates the imaging of the PSs *in vivo* and enhances the PDT treatment. However, it must also be noted that the combination of PDT and optical imaging still has a relatively short tissue-penetration in comparison to other imaging modalities covered in this review and thus, the capabilities for monitoring the biodistribution of the respective drugs by optical imaging remains limited.

Another promising group of theranostic compounds are molecules, which contain both a PS and a contrast agent for MRI. This combination links the high resolution and deeper penetration range of the purely diagnostic MRI with the light-induced treatment *via* PDT. Such theranostic agents have shown an increased tumor uptake and stronger anti-tumor effects than unmodified PS. The designed compounds exhibit a boosted relaxivity compared to traditional contrast agents, which would allow for a reduction of the administrated dose and therefore potentially reduce side-effects. In addition, such multimodal agents would significantly expand possible medical applications.

The third combination covered in this review are radiolabeled PSs for either PET or SPECT imaging, which couples the near unlimited penetration and high sensitivity of nuclear imaging with selective therapeutic applications of PDT. This strategy counters issues of optical imaging with PDT agents in terms of the usually very limited tissue penetration range. So far, various radionuclides - like ^99m^Tc and ^111^In for SPECT or ^18^F and ^68^Ga for PET - were already successfully used preclinically in (bio)conjugates together with PSs for the non-invasive and real-time monitoring of the accumulation of the multimodal agents by nuclear imaging and treatment *via* PDT *in vivo*. Hereby, this combination enabled the targeted treatment of tumor tissue after sufficient accumulation, the identification of drugs of suboptimal pharmacokinetics based in imaging data or biodistribution experiments, as well as the online monitoring by imaging during the PDT treatment with great efficiencies and in deeper tissues. Currently, all these novel theranostic approaches with PDT are still in the preclinical phase but show great promise for medical applications and translation into the clinic.

Overall, theranostic agents combining PDT with imaging modalities hold immense potential for targeted and image-guided treatment of various diseases, by offering additional information, identifying insufficient therapeutic options prematurely and reducing the rate of incorrect diagnoses. Ongoing challenges include the limited specificity of the drug delivery of several PSs, their low photostability and poor solubility, the negative impact added functional moieties can have on their photophysical properties, as well as the poor light penetration rate in biological tissues. In recent years, the development of new types of PSs with increased stability and PDT efficacy, as well as the conjugation of targeting, stabilizing or solubilizing moieties to sidechains of the PSs, and *via* longer chemically inert or cleavable linkers have shown encouraging results. Furthermore, modifications to shift the absorption and emission of the PSs further into the NIR range - e.g. compounds capable of multiphoton-excitation - to improve the light penetration, have proven promising, but further research in those fields remains highly necessary. Addressing these challenges, along with fostering interdisciplinary collaboration and investing in research and clinical trials, will contribute to improving current systems and ultimately benefiting patients.

## Figures and Tables

**Figure 18:**
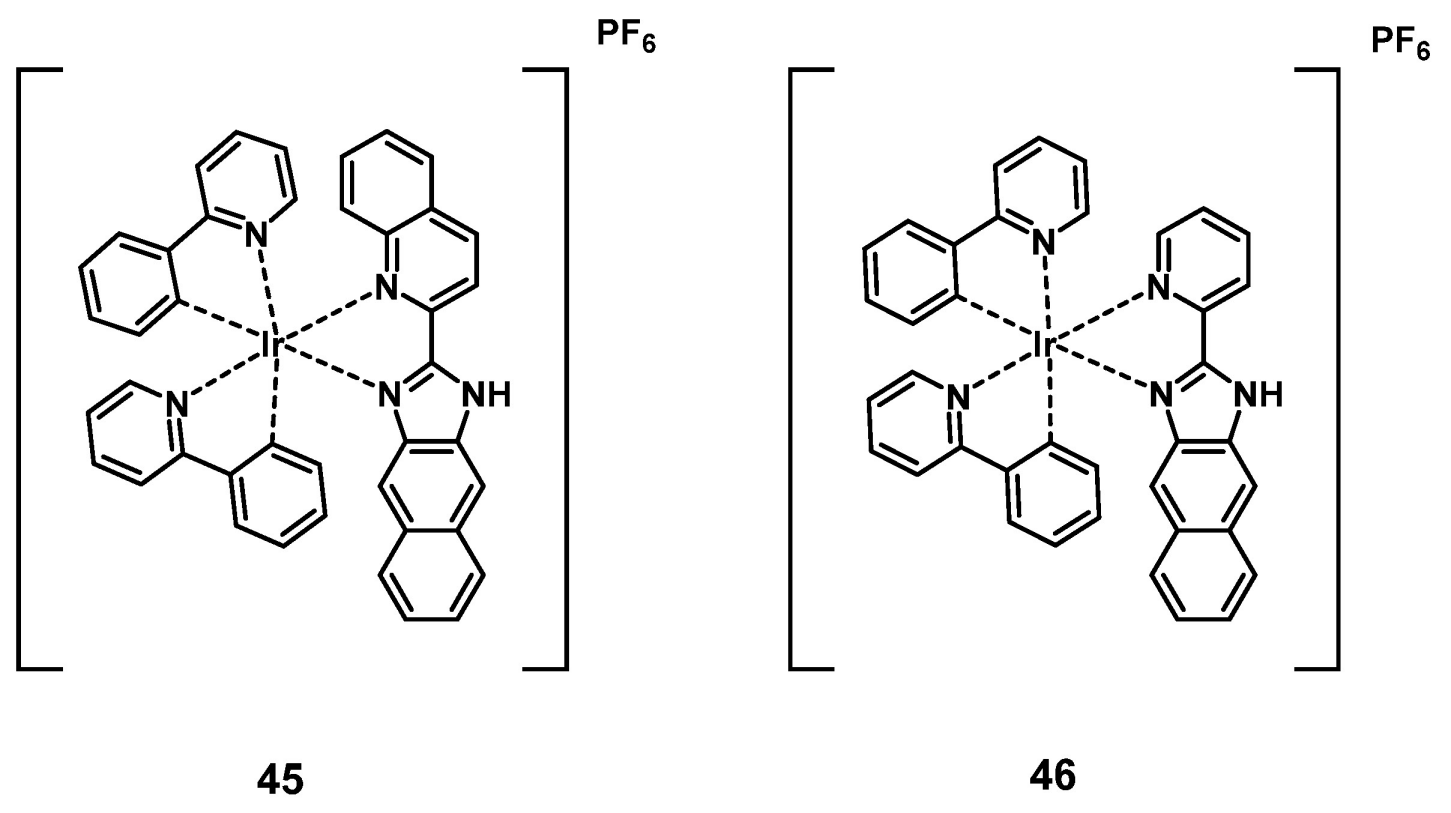
Structures of two kinase-targeting Ir-complexes with red-to-NIR luminescence and PDT activity 101.

**Figure 22:**
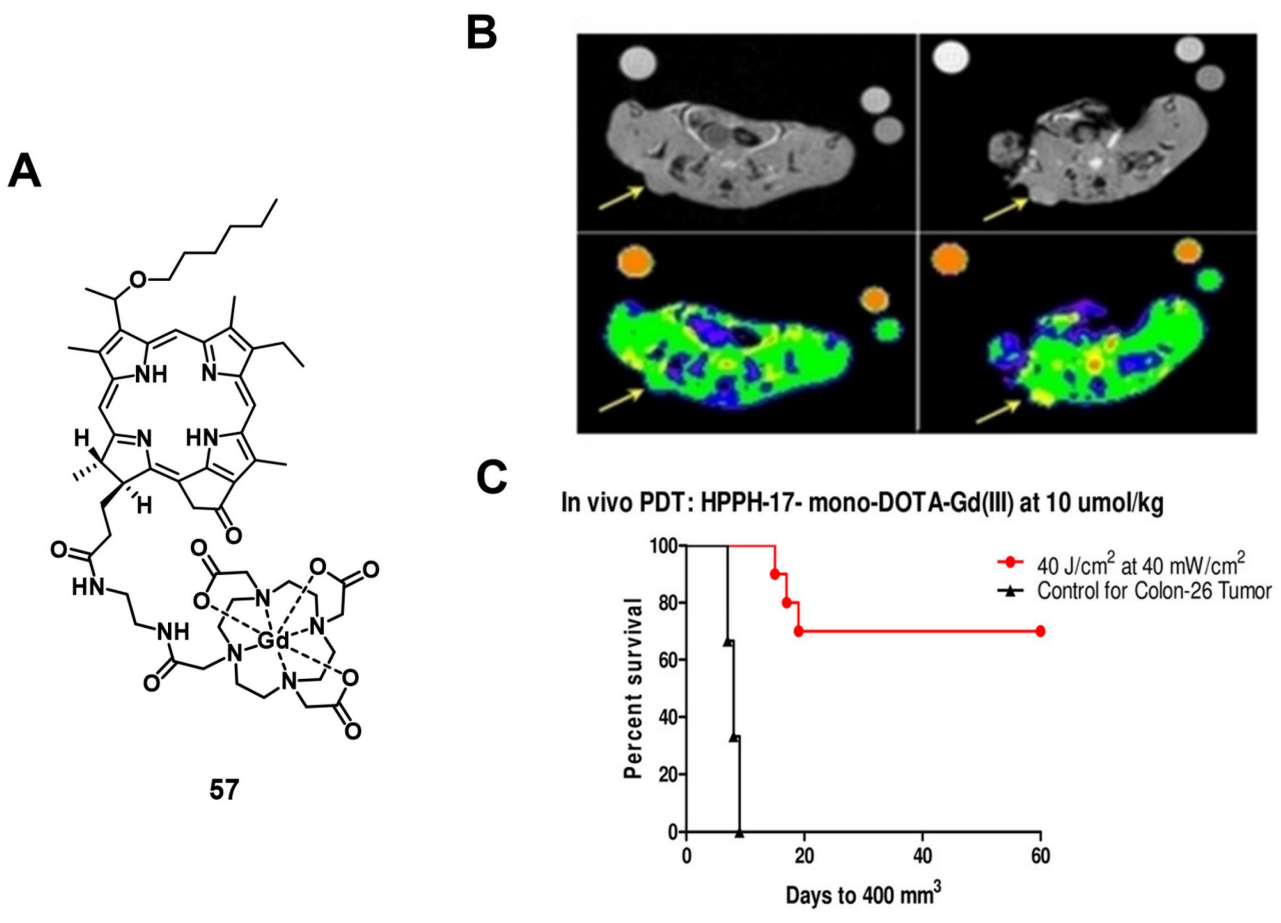
(A) Chemical structure of a theranostic compound combining PDT and MRI *via* conjugating Gd(III)-DOTA with HPPH. (B) Increase in MRI signal intensity after treatment with compound **57** formulated in PBS (left: baseline, right: 4h p.i, arrows indicating tumor location). Bottom figures overlaid with false color representing the change. (C) In vivo PDT efficacy of **57** on mice bearing Colon-26 tumors. Figures reproduced with permission from Ref. 117 Copyright 2020 Wiley-VCH GmbH.

**Figure 24:**
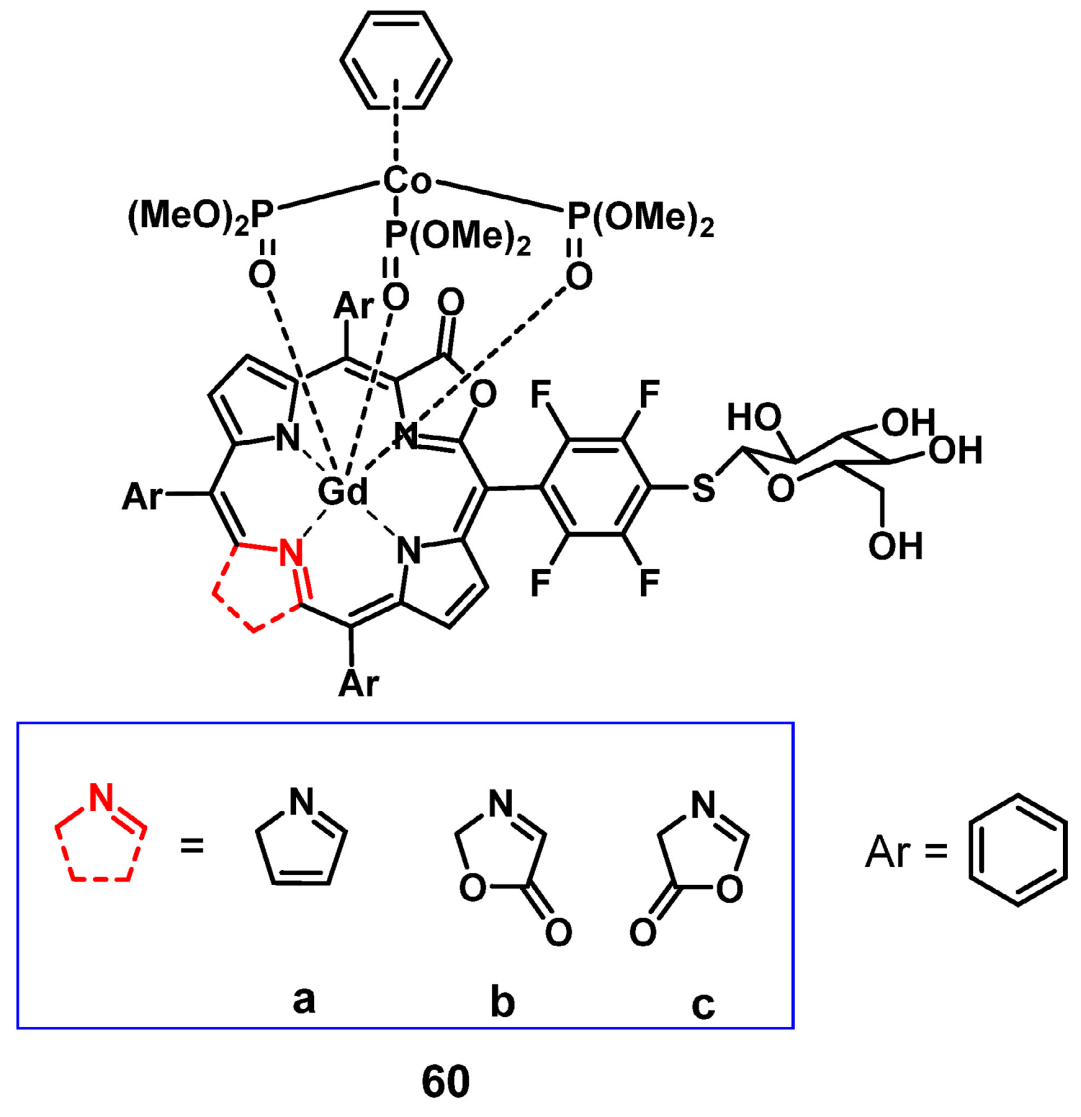
Gadolinium porphyrinoids as phototheranostic compounds using PDT and MRI 119.

**Table 1:** Commonly used radionuclides in PET imaging 133, 134

	^11^C	^18^F	^64^Cu	^68^Ga	^124^I
Half-life	20.3 min	110 min	12.7 h	68.1 min	4.2 days
Max. β^+^ energy (MeV)	0.97	0.64	0.66	1.90	2.14

**Table 2:** Commonly used radionuclides in SPECT imaging 133

	^67^Ga	^99m^Tc	^111^In	^123^I	^201^Tl
Half-life	78.3 h	6.0 h	67.9 h	13.2 h	73.1 h
Photon Emission Energy (MeV)	0.09 / 0.19 / 0.3	0.14	0.17 / 0.25	0.16	0.17

**Table 3:** Cell lines mentioned in the review

16HBE	Human bronchial cells (epithelial)
3T3	Murine embryotic carcinoma (fibroblastic)
3T3-HER2	• 3T3 transfected with HER2
4T1	Murine mammary carcinoma (epithelial)
A4	Murine, immortalized fibroblast cells • 3T3 transfected with human EGFR-2
A431	Human epidermoid carcinoma (epithelial)
A549	Human lung carcinoma (epithelial)
A549R	• Cisplatin resistant sub-strain
B16-F10	Murine melanoma
BT20	Human mammary carcinoma (epithelial)
BxPC3	Human pancreatic adenocarcinoma (epithelial)
CT26	Murine colon adenocarcinoma (fibroblastic)
Colon 26	Murine colon adenocarcinoma (epithelial-like)
DU145	Human prostate carcinoma (epithelial)
DS sarcoma	Human lymphoma (hematopoietic-like)
EAT	Murine mammary adenocarcinoma (epithelial-like)
EMT-6	Murine mammary carcinoma (epithelial)
GIST-T1	Human stomach carcinoma
GL261	Murine glioblastoma (fibroblastic)
H1299	Human lung carcinoma (epithelial)
HeLa	Human cervical adenocarcinoma (epithelial)
HepG2	Human hepatocellular carcinoma
HT1080	Human fibrosarcoma (epithelial)
HT29	Human colon adenocarcinoma (epithelial)
HUVEC	Human umbilical vein (endothelial)
H460	Human non-small cell lung cancer
KB	Human endocervical adenocarcinoma (epithelial-like)
LLC	Murine lewis lung carcinoma
LO2	Human endocervical adenocarcinoma - papillomavirus-related
LS174T	Human colorectal adenocarcinoma (epithelial)
LS174T-PSMA	Transfected with pcDNA3 (encoding PSMA)
MAC13762	Human mammary mucinous adenocarcinoma
MCF-7	Human mammary adenocarcinoma (epithelial)
MDA-231	Human mammary adenocarcinoma (epithelial)
MDA-MB-231-GFP	• Gene for GFP expression introduced *via* lentivirus
MDA-MB-231-LUC	• Gene for firefly luciferase expression and Neomycin resistance introduced
MDA-MB-43	Human breast cancer
MRC5	Human lung (fibroblastic)
MT-1	Human leukemia
NCI-H460	Human lung carcinoma (epithelial)
NSCLC	Human non-small cell lung carcinoma (patient derived)
OVCAR-3	Human ovary adenocarcinoma (epithelial-like)
Panc-1	Human pancreatic carcinoma (epithelial)
PC3	Human prostate adenocarcinoma (epithelial)
PC3-pip	• Transfected with PSMA
PC3-flu	• Transfected with flu-peptide
QBC-939	Human cholangio carcinoma
RIF-1	Murine radiation-induced fibrosarcoma
RGM-1	Murine gastritic cells (epithelial)
SCLC	Human small cell lung carcinoma (patient derived)
SKOV-3	Human ovary adenocarcinoma (epithelial)
SMMC-7721	Human endocervical adenocarcinoma - papillomavirus related
SW620	Human colorectal adenocarcinoma (epithelial)
U87	Human glioma - likely glioblastoma (epithelial)
UMUC3	Human bladder transitional carcinoma (epithelial)
WiDr	Human colorectal adenocarcinoma (epithelial)

**Table 4:** Animals mentioned in the review

ATHYM-Foxn1	Outbred athymic nude immunodeficient mice
BALB/c	Inbred, immunodeficient, albino mice
BALB/cAnNCr	• 2 specific quantitative trait loci (Uvbi1 and Uvbi2)
BALB/cAJcI-nu/nu	• Autosomal recessive nude gene (sub-strain)
BALB/c nu/nu	• Autosomal recessive nude gene (sub-strain)
C3H	Inbred mice with endotoxin resistance
C57BL/6	Inbred black mice
CD-1	Outbred albino mice
NCRNU-M	Outbred athymic nude immunodeficient mice
SCID	Albino mice with severe combined immunodeficiency disease
Sparague Dawley	Outbred albino rats

## Abbreviations

5-ALA: 5-aminolevulinic acid
α_V_β_3_: “vibronectin receptor”, formed by integrin α_V_ and integrin β_3_
ABCG2: ATP binding cassette subfamily G member 2
AIE: aggregation induced emission
ATP: adenosine triphosphate
BBN: bombesin
BODIPY: thieno-pyrrole fused boron-dipyrromethene dye
BRET: bioluminescence resonance energy transfer
CA: contrast agents
CPA: cyclophosphamide
CT: computed tomography
EGFR: epidermal growth factor receptor
Epi: Epirubicin
EPR: enhanced permeability and retention
ER: endoplasmatic reticulum
[^18^F]FDG: 2-fluorodeoxyglucose radiolabeled with^ 18^F
FRα: folate receptor-α
FRET: Förster resonance energy transfer
GFP: green fluorescent protein
GGT: γ-glutamyl-transpeptidase
GIST: gastrointestinal stromal tumor
GRP: gastrin-releasing peptide
H: hour
HCPT: 10-hydroxycamptothecin
HPPH: Photochlor
IA: injected activity
IC_50_: half maximal inhibitory concentration
ICG: Indocyanine green
ID: injected dose
ISC: intersystem crossing
iv.: intravenous
LD_90_: lethal dose, 90%
LDL: low-density lipoprotein
min: minute
MMP7: matrix metallo-proteinase 7
MRI: magnetic resonance imaging
NIR: near infrared
NIR-I: first near-infrared window
NIR-II: second near-infrared window
NIR-III: third near-infrared window
NHS: N-hydroxysuccinimide
NMU: N-nitroso-methylurea
^1^O_2_: singlet oxygen
O_2_^•-^: superoxide anion radical
PCa: prostate cancer
PcS: sulfophthalocyanine
PDT: photodynamic therapy
PEG: polyethylene glycol
PET: positron emission tomography
p.i.: post injection
PDX: patient derived xenograft
PS: photosensitizer
PSMA: prostate specific membrane antigen
PTT: photothermal therapy
RGD: tripeptide of arginine-glycine-aspartic acid
ROS: reactive oxygen species
RT: radiation therapy
S_0_: ground state
S_1_: excited singlet state
SPECT: single photon emission computed tomography
t_(½)_: half-life
T_1_: excited triplet state
T1: longitudinal relaxation time
T2: transverse relaxation time
TDHPP: 5,10,15,20-tetrakis(3,5-dihydroxyphenyl)porphyrin)
THPP: 5,10,15,20-tetrakis(4-hydroxyphenyl)porphyrin
TDMPP: 5,10,15,20-tetrakis(3,4-dimethoxyphenyl)porphyrin)
TPPNH_2_: 5,10,15,20-tetrakis(4-aminophenyl)porphyrin
TSPO: translocator protein
UV/Vis: ultraviolet and visible
VHH: variable domain heavy chain only antibody

## Biographies

** Youchao WANG** obtained her MSc in organic chemistry (2020) at the Technical Institute of Physics and Chemistry, Chinese Academy of Sciences in Beijing. In 2020, she was awarded a China Scholarship Council to carry out her PhD studies under the supervision of Prof. Gilles Gasser at Chimie ParisTech, PSL University. She is currently working on the development of organometallic-based theranostic drugs, which combine photodynamic therapy, nuclear imaging and radiotherapy.

** Johannes Nikodemus STAUDINGER** obtained his MSc in chemistry (2021) at the University of Vienna. In 2022 he started his PhD studies under the supervision of Prof. Thomas Mindt at the University of Vienna. Currently he is working on the development of new theranostic compounds, which combine nuclear imaging, radiotherapy and photodynamic therapy.

** Thomas L. Mindt** became Professor in radiopharmaceutical chemistry at the University of Basel (Switzerland) in 2009. In 2016, he moved to Vienna (Austria) as a co-founder of the Ludwig Boltzmann Institute Applied Diagnostics and became Professor in Bioinorganic Radiochemistry at the University of Vienna in 2020. Tom received several fellowships and awards including the Young Investigator Award of the Society of Radiopharmaceutical Sciences and the Max Lüthi Honor of the Swiss Chemical Society. Tom´s research focusses on the use of metallic radioisotopes for diagnostic and therapeutic applications in nuclear medicine.

** Gilles Gasser** started his independent scientific career at the University of Zurich (Switzerland) in 2010 before moving to Chimie ParisTech, PSL University (Paris, France) in 2016 to take a PSL Chair of Excellence. Gilles was the recipient of several fellowships and awards including the Alfred Werner Award from the Swiss Chemical Society, an ERC Consolidator Grant and Proof of Concept, the European BioInorganic Chemistry (EuroBIC) medal and the Pierre Fabre Award for therapeutic innovation from the French Société de Chimie Thérapeutique. Gilles' research interests lay in the use of metal complexes in different areas of medicinal and biological chemistry.
